# A review of Optical Point-of-Care devices to Estimate the Technology Transfer of These Cutting-Edge Technologies

**DOI:** 10.3390/bios12121091

**Published:** 2022-11-29

**Authors:** María Jesús Pioz, Rocío L. Espinosa, María Fe Laguna, Beatriz Santamaria, Ana María M. Murillo, Álvaro Lavín Hueros, Sergio Quintero, Luca Tramarin, Luis G Valle, Pedro Herreros, Alberto Bellido, Rafael Casquel, Miguel Holgado

**Affiliations:** 1 Optics, Photonics and Biophotonics Group, Center for Biomedical Technology, Optics, Universidad Politécnica de Madrid, Campus Montegancedo, Pozuelo de Alarcón, 28223 Madrid, Spain; 2 University of Nebrija, C/del Hostal, Campus Berzosa, 28248 Madrid, Spain; 3 Group of Organ and Tissue on-a-Chip and In-Vitro Detection, Health Research Institute of the Hospital Clínico San Carlos IdISSC, C/Profesor Martín Lagos s/n, 4ª _Planta Sur, 28040 Madrid, Spain; 4 Department of Applied Physics and Materials Engineering, Escuela Técnica Superior de Ingenieros Industriales, Universidad Politécnica de Madrid, C/José Gutiérrez Abascal 2, 28006 Madrid, Spain; 5 Metch, Chem & Industrial Design Engineering Department, Escuela Técnica Superior de Ingeniería y Diseño Industrial, Universidad Politécnica de Madrid, Ronda de Valencia 3, 28012 Madrid, Spain; 6 Multiplex Molecular Diagnostics S.L. C/ Munner 10, 08022 Barcelona, Spain

**Keywords:** optical Point-of-Care (PoC), patent, biosensor, medical device, optic

## Abstract

Despite the remarkable development related to Point-of-Care devices based on optical technology, their difficulties when used outside of research laboratories are notable. In this sense, it would be interesting to ask ourselves what the degree of transferability of the research work to the market is, for example, by analysing the relation between the scientific work developed and the registered one, through patent. In this work, we provide an overview of the state-of-the-art in the sector of optical Point-of-Care devices, not only in the research area but also regarding their transfer to market. To this end, we explored a methodology for searching articles and patents to obtain an indicator that relates to both. This figure of merit to estimate this transfer is based on classifying the relevant research articles in the area and the patents that have been generated from these ones. To delimit the scope of this study, we researched the results of a large enough number of publications in the period from 2015 to 2020, by using keywords “biosensor”, “optic”, and “device” to obtain the most representative articles from Web of Science and Scopus. Then, we classified them according to a particular classification of the optical PoC devices. Once we had this sampling frame, we defined a patent search strategy to cross-link the article with a registered patent (by surfing Google Patents) and classified them accordingly to the categories described. Finally, we proposed a relative figure called Index of Technology Transference (IoTT), which estimates to what extent our findings in science materialized in published articles are protected by patent.

## 1. Introduction

Scientific studies define a Point-of-Care (PoC) device as a test performed near the patient to support clinical decision making. Such a test must be carried out during or very close to the time of consultation, and on any part of the patient’s body [1,2,3,4,5,6]. The qualifications of the staff, in principle, should be lower than in the case of complex diagnostic systems. These systems offer an accurate, rapid, and low-cost approach to diagnosing diseases noninvasively. The technological basis for the read-out-platforms classifies PoCs into different types. Concretely, this work is focused on Optical PoCs devices, which can be defined as portable devices based on the use of light to determine specifically the recognition of target biomolecules or biomarkers (biosensing response) into a sensitive variable of detection [7].

Several works have been published related to PoC devices [8,9,10,11]. However, as far as we know, the relationship between scientific research and registered patents seems to be a missing link which still needs addressing. This work attempts to cover this gap by inferring how the generation of knowledge embodied in the publication of scientific papers is reflected upon registered patents in the area of optical PoC devices delimited by the key words: “biosensor”, “optic”, and “device”. Thus, a relative figure of merit to evaluate this can be the percentage of patents coming from the research articles analysed. We call to this figure the Index of Technology Transference (IoTT) of this area.

We firstly classified the optical devices that are mostly discussed in the literature, according to their read-out techniques. The technical analysis and classification are introduced in Section 2.1. Regarding the main characteristics and advantages of a PoC, from the engineering perspective, the miniaturization and simplification of the optical device and the high performance of the sensor design makes possible the achievement of a sensitivity comparable to that from a laboratory [12].

Concerning the read-out platform, to read the information of the biosensor, PoC devices usually have a user-friendly interface to allow relatively inexperienced personnel to operate the device. Furthermore, many of them can send the results to the medical institutions through a wireless network of intelligent equipment, facilitating end-user connectivity with a minimal manufacturing cost.

This kind of cost-effective device versus the bulky diagnosis instruments has benefits such as portability, affordability [13,14], and connectivity with the patients, making the PoC devices ideal for widespread deployment in developing countries. Traditional diagnostic devices such as PCR, ELISA, and microscopic instruments are not always available in developing countries due to economic and technical limitations [3,15,16,17,18,19,20,21,22,23,24]. From the economic perspective, the reduction in costs is not only reflected upon the final price of the PoC itself, but also in the decrement of collateral costs derived from the use of this technology (i.e., staff, facilities, and equipment).

**Figure 1:**
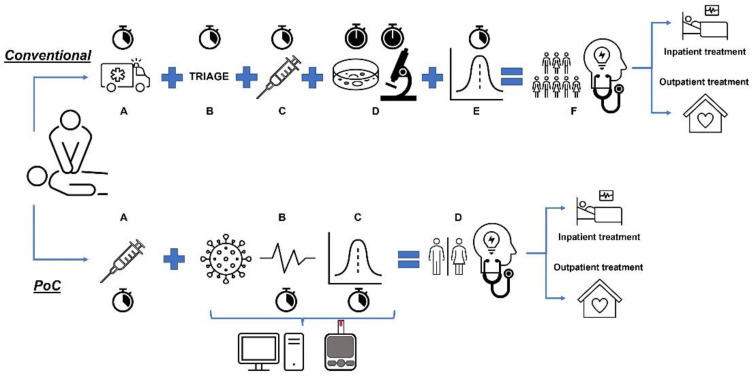
A schematic representation of the resources needed to achieve a diagnosis: PoC versus Conventional Procedure. On the top, the Conventional option shows the process until getting a diagnostic in the conventional procedure (i.e., Transfer hospital [A] + Triage [B] + Sample Collection [C] + Sample processing in the lab [D] + Results [E]) to make the diagnosis, and to indicate the treatment at home, or in the hospital (i.e., Analysis medical staff [F]). At the bottom, the PoC option confirms the reduction of the process: Sample Collection (A) + Analyze results PoC (B)+ Results (C: in a few minutes), to get a diagnostic with the use of PoC (D).

The time reduction to obtain a diagnosis is a cornerstone of the PoC tests, being relevant for both the health personnel involved in the diagnosis and for the patient. PoC tests are normally based on high-tech devices which can yield results in a short time, just by taking a sample of someone’s fluid (e.g., blood, urine, saliva, tears, and semen) and detecting different targets molecules (i.e., proteins, nucleic acids, metabolites, and drugs) [25]. For descriptive purposes, Figure 1 compares the conventional procedure with the use of the PoC. On the top, the Conventional option shows the process until obtainment of a diagnostic in the conventional procedure (i.e., Transfer hospital [A] + Triage [B] + Sample Collection [C] + Sample processing in the lab [D] + Results [E]) to make the diagnosis, and to indicate the treatment at home, or in the hospital (i.e., Analysis medical staff [F]). At the bottom, the PoC option confirms the reduction in the process: Sample Collection (A) + Analyse results PoC (B)+ Results (C: in a few minutes), to obtain a diagnostic with the use of PoC (D). It shows the advantages of the PoC option, related to the reduction in time, staff, and required technology, to obtain a diagnosis and to implement an early treatment.

Concerning detection, there are three types of identified measurements: (i) qualitative, to detect the presence or absence of a marker in a specimen; (ii) quantitative, to determine the amount or concentration of a marker in a specimen; and (iii) semiquantitative, to screen specimens on certain markers in a certain concentration range. Despite the type of measurement, the Optical PoC technology allows extremely parallelized (i.e., multiplexed) and fine-tuned measurements, surpassing on many occasions the precision of conventional analysis [26,27].

Considering the versatility of the PoC devices already summarized, it is noteworthy that such versatility is reflected on a wide range of targets which can be achieved [28].

**Figure 2:**
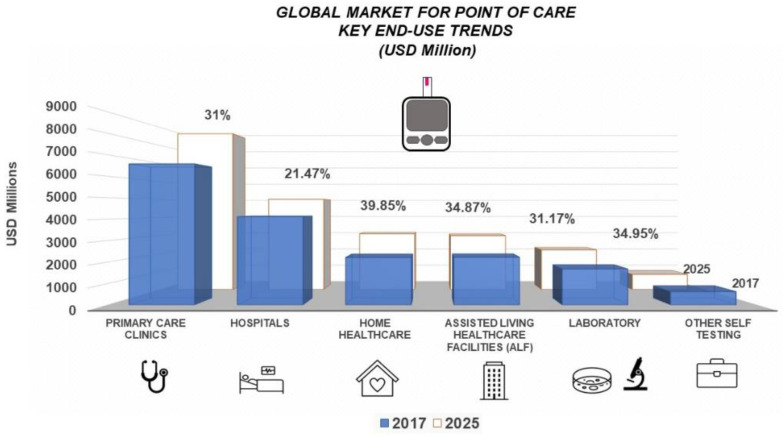
A schematic forecast of the Global market for PoC 2017–2025. This implies that PoC Technology can be performed in the following environments: Primary Care Clinics, Hospitals, Home Healthcare, Assisted Living Healthcare Facilities, Laboratory, and other Self Testing Areas. (Data extracted by Grand View Research´s report—See [29]).

**Figure 3:**
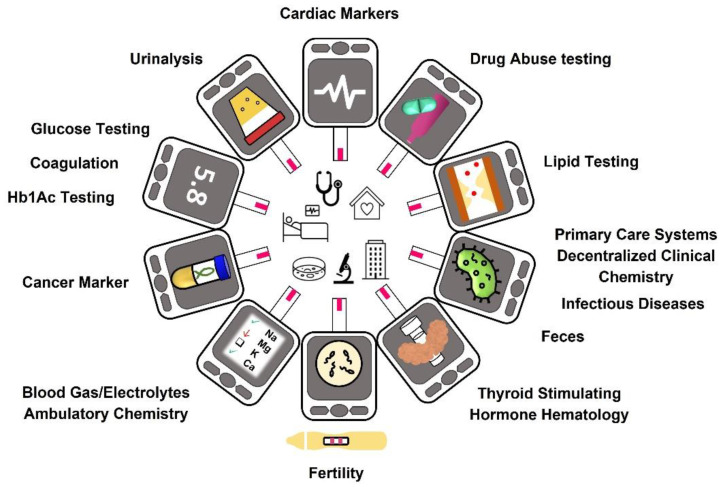
PoC Diagnostics Market and its segmentation attending to the target analysis and end-use: The market is classified in 14 different products according to pathology diagnostic, and in five kinds of products attending to the end use. (Data extracted from Grand View Research´s report [29]).

Figure 2 represents the Global market for PoC from 2017 to 2025, and Figure 3 illustrates this diversity focused on the type of product marketed (Glucose Testing, Hb1Ac Testing, Coagulation, Pregnancy and Fertility, Infectious Diseases, Cardiac Markers, Thyroid Stimulating Hormone, Haematology, and Primary Care Systems). The trend is expected to grow in each of the shown areas, where the average growth amounts to 32.25%.

The most attractive feature of PoC is that the detection can be performed on-site and in real-time, without the need for additional peripheral equipment, and the success of PoC diagnostics heavily depends on the continued development of alternative medical technologies that are cost-effective, sensitive, and sufficiently accurate. As has been mentioned, there are many advantages of the PoC devices; however, the challenge to translate researched technologies to the market for global health diseases is well known to both the scientific and business sectors. After the development of the laboratory prototype, several years are required to manufacture a product capable of entering the market because it requires a remarkable technical effort and significant investment to reach a truly marketable product in response to market needs.

The bridging of this gap needs very close collaboration between the scientists, who develop the device coming from the different disciplines (e.g., Chemistry, Biochemistry, Optics, Engineering…), and the practitioners and professionals of health working together from the beginning from the conception of the initial idea of the final device. This covers a need to avoid the development of devices which have never had the possibility of operating in a real clinical setting [30].

This review aims to research a relevant scope such as optical PoC device through the bounded analysis and classification of the knowledge derived from the selected articles. We limited our study to the chosen keywords, and we performed an exhaustive analysis of published articles in this area and the registered patents generated from these, to study the infer the transfer.

To deepen our understanding, we proposed a methodology to estimate the transfer of technology by obtaining the number of articles published versus related patents. Finally, we obtained, for the first time, a figure of merit as a technology transfer indicator, which we have termed IoTT. In this review, we placed a special emphasis on the role of one of the requirements for an invention to be patentable, and that is the element of novelty. Understanding novelty as some new characteristic which is not known in the body of existing knowledge in its technical field. This body of existing knowledge is called “prior art” [31] which implies first to protect the invention and later, once the novelty is guaranteed, the scientific article can be published.

**Figure 4:**
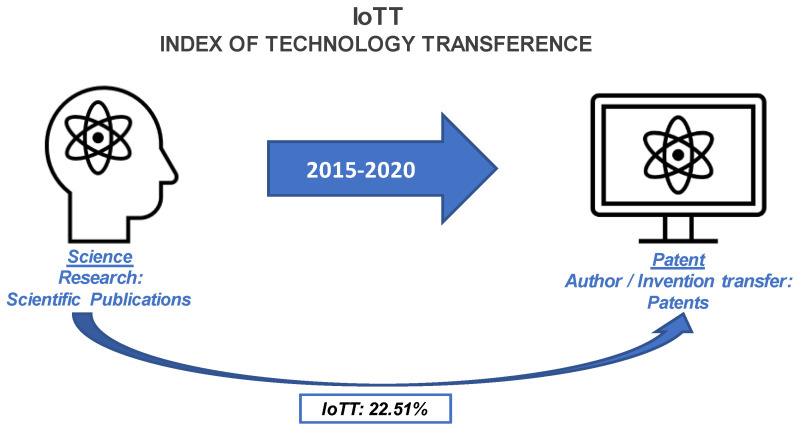
Index of Technology Transference (IoTT): Percentage of transfer between Science and Patent between the Period: 2015–2020.

Considering this, the developed methodology implies the link of articles in which the research has been registered previously in a patent. The results obtained with our methodology revealed the IoTT for the Optical PoC devices (see Figure 4).

The methodology will be explained in detail later, in Section 2, and it is summarized in Figure 4, but briefly, we initially identified 744 publications as potentially eligible for inclusion. The inclusion criteria comprised the search of publications including the keywords biosensor, optic, and device. After excluding duplicates and non-eligible documents, we studied 151 scientific articles and sorted them according to different criteria. The review of their quantitative and qualitative aspects was performed following the established classification. Concretely, we established the following criteria: (i) the detecting techniques (i.e., label-free vs. labelled detection); (ii) the excitation monitoring (i.e., wavelength λ vs. angle of incidence ϕ); (iii) the optical interrogation technique (i.e., vertical vs. horizontal); and (iv) the variation of the complex refractive index (i.e., the real vs. imaginary part, and emission and scattering). Afterwards, the authors were crossed with inventors to crosslink article and the registered patent. This relation was considered only when the patents claimed for at least a part of the PoC device referred in the included scientific publication. It is worth mentioning the difficulty of assuring this link, because, although the authors are the same as the inventors, and the topic of the article and the patent are similar, it is exceptional to find a patent with the exact theme of the article. Notwithstanding this limitation, we classified the patents following the same methodology as for the article, being able to link registered patents from articles, leading to the IoTT.

## 2. Methods

Given that the gap in crosslinking scientific publications and patented devices on the research field related to Optical PoC devices exists, we carried out this review with a clear target of covering this missing link. Therefore, the current search strategy was designed to combine conventional systematic review principles and methods, with others more oriented to the search and study of patents, which allowed us to ensure the quality of this study.

**Figure 5:**
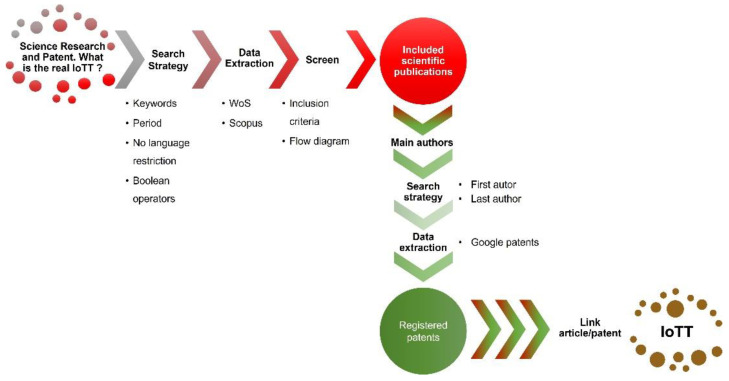
Structure of the used methodology, from the question to answer, to the calculation of the IoTT.

Concretely, this review comes from a structured question and a prior research protocol with an explicit method, mainly designed to answer a concrete question: Optical Point-of-care: Scientific Research and Patent. What is the real index of technology transfer of these technologies? The developed methodology (summarized in Figure 5) was applied in both sectors of research, that is articles and patents.

The underlying techniques of the Optical PoC devices are relevant to classify them into different categories. Hence, a deep study of such techniques was mandatory to establish a set of categories which included the research articles found. Therefore, the first part of this section summarizes the considered optical features and the description of the consequent classes.

Afterwards, the following subsection will describe in detail the methodology developed to find the articles, the criteria to classify them and to find the link between articles and patents.

### 2.1. Classification of Optical Read-Out Devices—Overview of the Principle and Applications

Optical PoC can be defined as a device based on light incidence, interaction with matter, and change in response used to detect or diagnose.

Notwithstanding the numerous methods in classifying optical PoCs devices, we set a particularized classification with the aim to perform a targeted assessment. Each of the criteria above mentioned will be explained below, and the selected publications will be included in their corresponding classifications.

#### 2.1.1. Classification Based on the Detecting Techniques

A feature usually used to classify optical biosensing is the requirement of a labelled molecule to measure the interaction between the bioreceptors and the target molecules. Hence, the first classification was set up by sorting PoC devices into label-free and label-based methods.

The labelled technology requires chemical amplification by means of fluorescent tags attached to molecules which determine the presence of the target molecules or the interaction between target molecules and bioreceptors. The need of several steps, labelled reagents, and specific laboratory equipment are some disadvantages for this technique [32].

Alternatively, the label-free optical technology directly measures the biomolecules and the interaction between them without any modification or labelling. Thus, the obtained signal is directly proportional to the concentration of biomolecules in their natural forms or to the biorecognition event [33,34].

#### 2.1.2. Classification Based on the Excitation Monitoring

Considering the variety of parameters to monitor the excitation of the sensors, we focused on the parameters to control the reflectivity and the intensity under a fixed angle of incidence, that is, angular or wavelength interrogation.

#### 2.1.3. Classification Based on the Optical Interrogation

Among the optical techniques developed to obtain the detectable signal, this classification is focused on the setup scheme for interrogating the sensor. Given the incidence of light, the interrogation technique can be performed vertically, horizontally or with other configurations.

**Figure 6:**
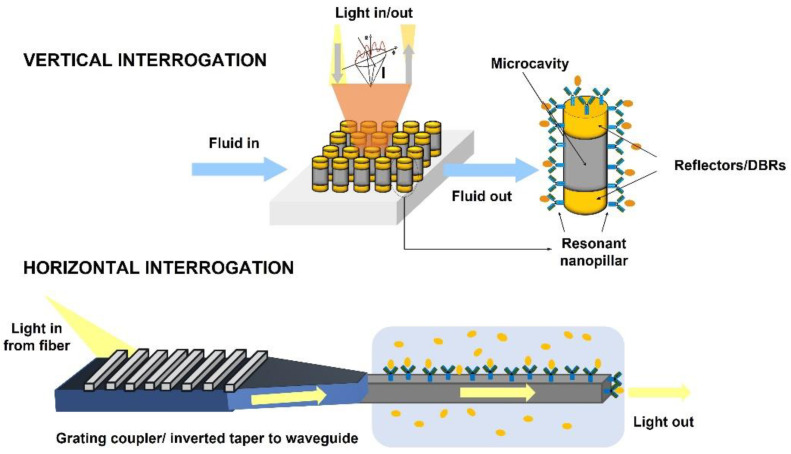
Schematic diagram of optical Interrogation, vertical (**top**) and horizontal (**bottom**). Depiction of the vertically characterized sensor based on an array of resonant nanopillars (**top**, from Casquel. R, Doctoral Thesis (2012) [35]). Depiction of the horizontally interrogated sensor (**bottom**). The light is coupled from the fibre to the waveguide by using a combination of a grating coupler and an inverted taper.

Vertical sensors are defined as optical devices interrogated at normal incidence, using generally a non-complex optical setup, with a light source, a focusing system, and a light collector, which can measure intensity (photodetector) or analyse the normally reflected or transmitted light (spectrometer, CCD camera). Figure 6 top shows an example of a vertically interrogated sensor. On the contrary, the horizontal interrogation is based on the coupling of the light through a waveguide to detect the signals after the interaction with the material (see Figure 6 bottom). The principle of detection of these planar devices is the change in the evanescent field of the light traveling through the waveguide before and after the bio-recognition. Configurations different from vertical or horizontal are more complex and setup-specific, thus, they will not be described here.

#### 2.1.4. Classification Based on the Variation of the Complex Refractive Index

The current classification is based on the properties of the light as an electromagnetic wave, and the effect of measuring it after having travelled through a material from one medium to another. The complex refractive index of the medium is a complex number (***n*** in Equation (1)) with a real part (***n***) which depends on the phase speed of the light in the medium, and an imaginary part which represents the coefficient of extinction (K) of the light in the medium (Santamaría, B. Doctoral Thesis (2020) [36]).

(1) n=n+i

Following the proposal of using the complex refractive index as a classifier, four subclasses were determined.

**Figure 7:**
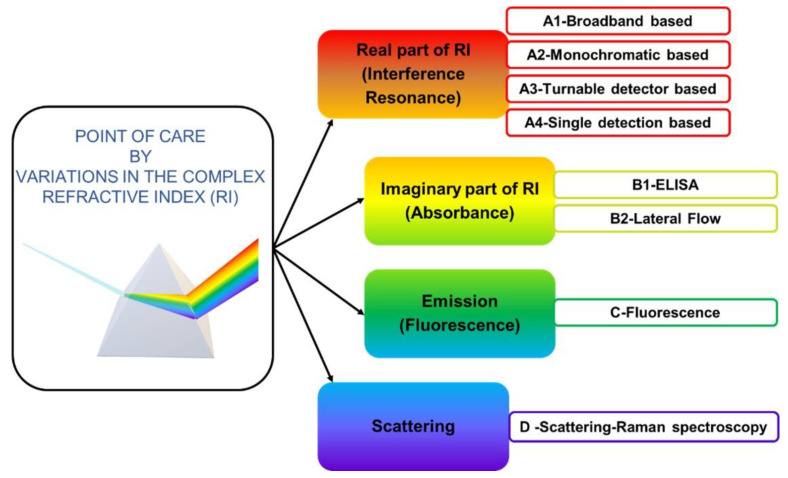
A schematic representation of the classification of PoC by optical detection method classified into four categories: Real part of RI (interference–resonance), Imaginary part of RI (absorbance), emission–fluorescence, and scattering.

Figure 7 summarizes the classification. First, the techniques based on measuring the variations of the real part were categorized as Real Part and Interference and subdivided into 4 subclasses, as it will be explained below. Second, techniques considering the imaginary part were stated as imaginary part and absorbance and were subclassified. Third, Emission focused on Fluorescence, and fourth Raman technique focused on measuring scattered light.

##### Real Part of RI—Interference/Resonance (A)

**Figure 8:**
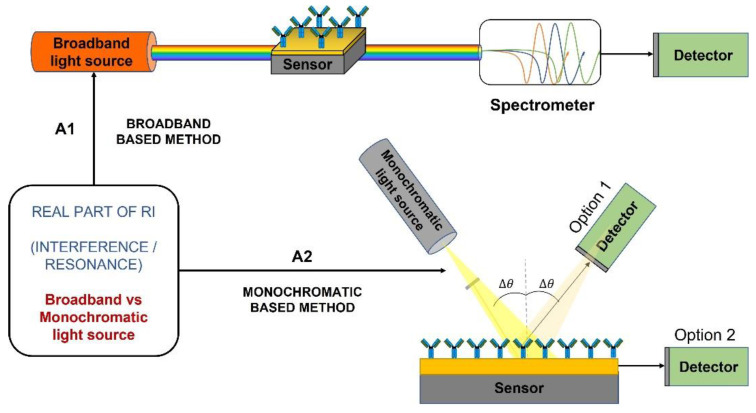
Classification based on the monitored changes in the real part of the refractive index: A1—Broadband Based/A2—Monochromatic Based or broadband source plus a monochromator.

**Figure 9:**
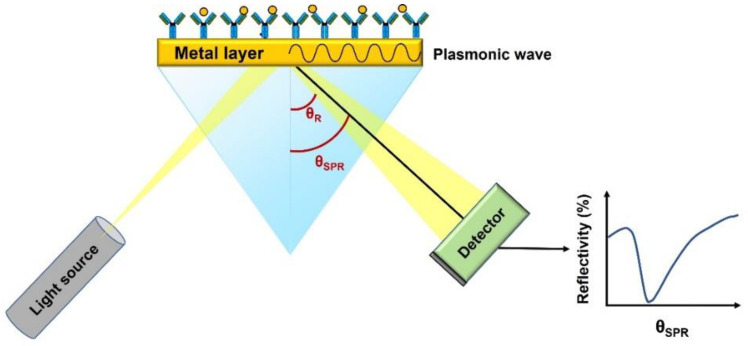
Sketch diagram of Surface plasmon resonance (SPR). Excitation of collective oscillations of electrons by the transverse magnetic polarized light at the metal interface.

Based on the monitored changes in the real part of the refractive index, we sorted devices considering the features of their sources of light by distinguishing between spectral band or monochromatic light (i.e., A1—Broadband Based and A2—Monochromatic Based in Figure 8). However, we also studied the capability of the detectors for multiple detection by means of turnability (A3—Turnable Detector Based in Figure 9) or for single detection (A4—Single Detection Based in Figure 9). In all the approaches the interference pattern is the cornerstone of the measurement techniques.

Briefly, in the Broadband-based devices, each frequency component from the source produces a different fringe velocity when it passes through the sensor, and thus, it has a different resonance peak associated with the detector, as it is shown in Figure 8 A1. The detection can be made by means of a monochromator. Interesting examples can be also observed avoiding the use of monochromators, interferometers, or spectrometers an innovative way for obtaining the read-out signals [7].

Conversely, the monochromatic light devices comprise a single-frequency light source and a photodetector with two preferred configurations. The detector can be placed at the same angle as the light source (option 1 in Figure 8 A1) or it can be placed normal to the waveguide (option 2 in Figure 8 A2).

Within the methods based on the variation of the real part of the RI, the Surface Plasmon Resonance (SPR) technique must be specially mentioned and described. SPR is an optical technique for detecting the interaction of two different molecules in which one is mobile, and one is fixed on a thin gold film. Indeed, it is an interference effect that exists at the interface of two media, a metal and a dielectric surface. At a specific angle of incidence, the resonance occurs, the electromagnetic wave partially couples to the metal surface and the field decays evanescently [37] (see Figure 9). The electrons from the metal moves by excitation leading to the plasmon. Such plasmon propagates directly dependent on the refractive index of the sensing medium in contact with the metal. The SPR devices are then based on the measurement of the variations of the reflected light [38].

SPR is widely found as the underlying optical technique in many scientific publications related to optical PoC devices in combination with other techniques. Concretely, it is mainly found related to the A1—Broadband-based methods, but also it can be found related to the A2—Monochromatic-based methods and to the A4—Single detection methods.

**Figure 10:**
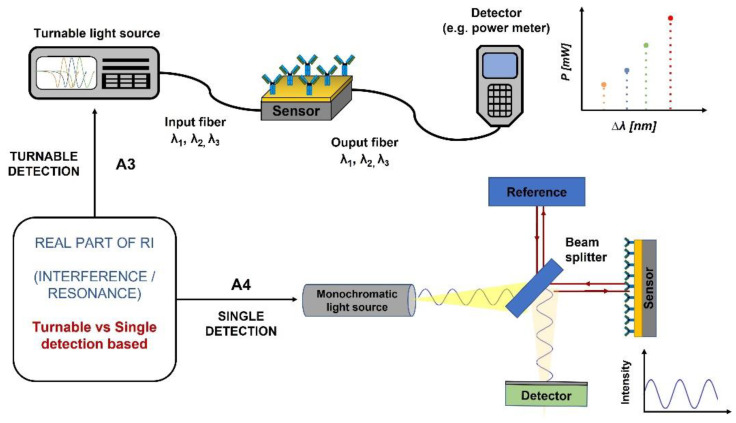
Classification based on the monitored changes in the real part of the refractive index. A3—Turnable Detector Based by using a turnable laser; and A4—Single Detection Based by a Michelson Interferometer.

Referring to turnable vs. single detection-based methods, devices based on turning systems select the wavelength at the light source and measure this wavelength at the detector. On the contrary, single detection methods are based on emitting and measuring only one wavelength. Both systems are represented in Figure 10. The turnable method is represented by an example of a turnable laser source of light (Figure 10 A3), while the single detection system is represented by a Michelson Interferometer scheme (Figure 10 A4).

##### Imaginary Part of RI—Absorbance (B)

**Figure 11:**
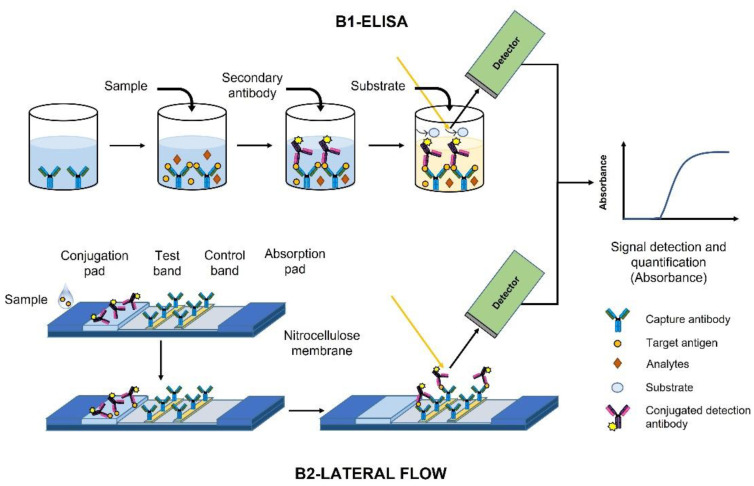
Classification based on the monitored changes in the imaginary part of the refractive index. B1—ELISA (**top**) and B2—Lateral Flow (**bottom**).

Based on the monitored changes in the imaginary part of the refractive index, we classified the devices according to the assay technique performed. The basis of these devices is the absorbance of the light by a sample, and the measurements at a specific wavelength [39]. On the one hand, the enzyme linked immunosorbent assay (ELISA) led to the B1—ELISA category. The ELISA technique is a plate-based assay technique designed for detecting and quantifying the target molecules in soluble substances (see Figure 11 B1). In an ELISA, the bioreceptor is immobilized on a solid surface (an antibody capture in Figure 11), then, the sample comprising the target molecules is added to promote the biorecognition event. Afterwards, a secondary antibody (i.e., a conjugated complex antibody linked to a reporter enzyme) is incubated. Finally, detection is accomplished by measuring the activity of the reporter enzyme via incubation with the appropriate substrate to produce a measurable product at a desired wavelength.

On the other hand, the paper-based technique, together with the platforms for the detection and quantification of analytes in complex mixtures, represents the B2—Lateral flow category. The lateral flow assays (LFA) are performed by immobilizing onto the paper the conjugated pad, the bioreceptors and the control molecules (see Figure 11 B2). The sample flows through the sensor until the detection place, where the absorbance of the light is measured.

Despite having similar characteristics with PoC, as its simplicity, fast process, and low cost, the lateral flow assay is considered an effective test, but not a PoC device.

##### Emission–Fluorescence (C)

**Figure 12:**
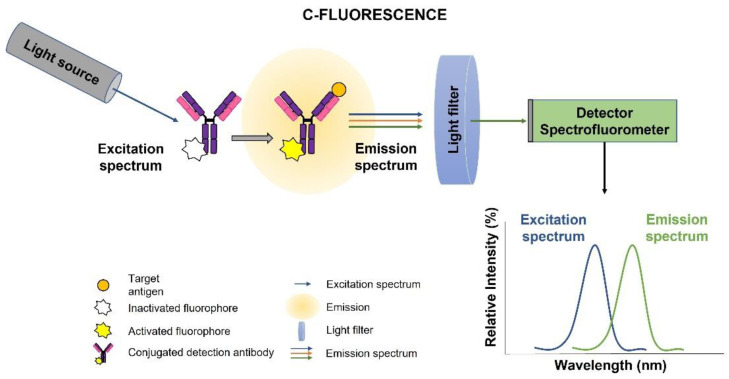
Fluorescence technique scheme to detect an antigen. The optical setup comprises a light source, a light filter and a spectrofluorometer.

Fluorescence PoCs were classified separately because these devices measure the emitted fluorescence light after the excitation at the desired wavelength. Fluorescence is an event in which a molecule is excited by a light source, after which this molecule will relax to a lower energy state while emitting a photon with different wavelength [39]. This emission can be detected using a spectrofluorometer with a light filter (see Figure 12). The fluorescence biosensor detects the concentration, location, and dynamics of biomolecules by the fluorescent phenomenon that occurs when electromagnetic radiation is absorbed by fluorophores or fluorescently labelled molecules so that the energy is converted into fluorescence emission [1,40]. The method of fluorescence detection in POCT has several advantages: good reproducibility, fast reaction time, low cost, on-site monitoring, and strong applicability [41].

##### Scattering-Raman Spectroscopy (D)

The light scattering is an optical phenomenon resultant from the incidence of the ray of light with an obstacle. The effect of such interaction is the deviation of the ray light, and depending on the changeability of the wavelength, it can be classified as elastic (i.e., unchanged wavelength) or inelastic (i.e., changed wavelength) [42]. Concretely, the Raman Scattering is an inelastic process where the kinetic energy of the incident photons is increased or decreased by interacting with molecular vibrations or rotations, phonons, or other excitations [1]. The Raman spectrum is unique for a material. Hence, the Raman is a label-free and non-invasive method which let to obtain both qualitative and quantitative information from any sample uniquely.

**Figure 13:**
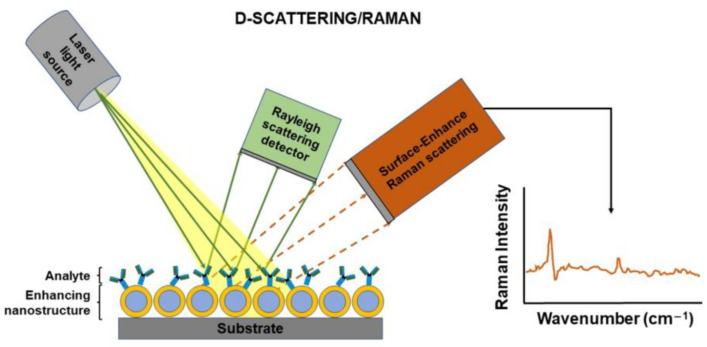
Schematic diagram of Raman spectroscopy (SERS).

There are more than 25 different types of known Raman spectroscopy techniques [43] among them, the surface-enhanced Raman scattering (SERS). Figure 13 illustrates the SERS technique, where the Raman signal obtained after interacting with the analytes is enhanced by means of the modification of the surface where such analytes are located.

### 2.2. IoTT Methodology

In order to answer our question and to obtain the IoTT, we collated all empirical evidence that fits pre-specified inclusion criteria, and we developed a comprehensive literature search by accessing databases including Web of Science (WoS) and Scopus and crossing them with Google Patents (January 2015 to December 2020).

Each of the platforms provides high-quality databases and the union of the results obtained using both WoS and Scopus for the search for papers, and the merging of Google Patents for the patent search, ensure the creation of a more complete database attending to our keywords.

As it is shown in Figure 5, the IoTT methodology was divided into two phases, the scientific publication search strategy, and the patent search strategy. Both will be described now, and then, the obtention of the IoTT.

#### 2.2.1. Scientific Publications Search Strategy

**Figure 14:**
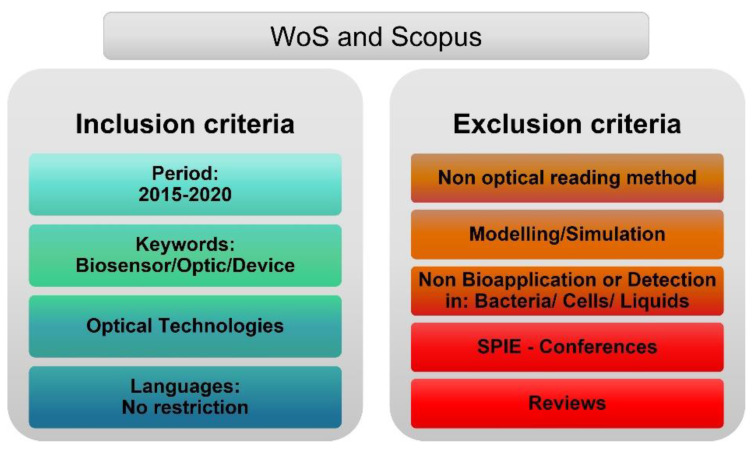
Inclusion–Exclusion Criteria (Web of Science and Scopus).

With the aim of gathering all the scientific articles related to Optical PoC devices, we carefully designed the search strategy by defining a control guideline with the keywords and the inclusion–exclusion criteria. The inclusion criteria (shown in Figure 14) consisted of studies comprising the following: (i) articles published between 2015 and 2020; (ii) including keywords “biosensor”, “optic”, and “device” (search made by Boolean operator “AND”); (iii) related to optical technologies. We did not exclude any article by language restriction.

On this review, we focused on the main optical technologies above described, hence, the exclusion criteria consisted of scientific studies about other technologies non specified in the methodology already described (i.e., variations on the Real or imaginary part of the RI, emission, scattering, and their sub-fields). Modelling and simulation articles were discarded. Moreover, non-bioapplication articles were excluded (i.e., articles based on detection of cells or bacteria or based on detection in liquids [14]). Finally, conferences publications and reviews were in the exclusion criteria.

We defined a control guideline before beginning the review and excluded all studies which did not fulfil such guideline. Concerning data selection and data extraction, it was a rigorous and explicit process of selection and evaluation of studies where two researchers (MJP, MFL) screened all included studies by titles and abstracts according to the eligibility criteria. Any disagreement in the study selection was resolved by consulting a third researcher (MHB).

Articles were included after the literature research and then were imported to an excel file in order to screen them. Screening was made according to inclusion and exclusion criteria specified in the Figure 14. To have a better judgment, the screening was developed in two phases. First, only the title and abstract were covered. Subsequently, full text screening took place for those studies that had not been excluded in the initial phase.

Eligible studies were then reviewed by all researchers and data were extracted independently using a pre-designed table sheet including title, sensor, read-out techniques (i.e., the real vs. imaginary part, and emission and scattering), optical interrogation technique (i.e., vertical vs. horizontal), excitation monitoring (i.e., wavelength λ vs. angle of incidence ϕ), detecting techniques (i.e., label-free vs. labelled detection), and bio application. After cross-check, any inconformity in data extraction was resolved by discussing or consulting with a third researcher (MHB). Once we had the selected scientific reports the patent search began.

#### 2.2.2. Patent Search Strategy

Despite the effort in applying the same methodology already described, we found a limitation related to the keywords that were selected for scientific articles. The reason is that the main intention of the patent ‘s inventors is to obtain the widest possible scope of patent protection. Therefore, the language used in patents is characterized by describing concepts in a very general way. Instead of using common terms to define the concept, the inventor uses as many words as possible in the description to allow a broader interpretation. In addition, sometimes patent applicants do not want others to find their patent applications and they avoid the use of intuitive words.

As consequence of trying to obtain more information and to find out the real Index of Technological Transfer among Science, Technology, and Market, we decided to perform two different searches. Firstly, we conducted an initial search with the same keywords as scientific publications, and despite the meticulous analysis of the patent database, the results of this study did not show any significant IoTT. The language used in the titles and abstracts of the patents did not allow links to the scientific articles, mainly due to the very different styles of writing of both types of documents.

To avoid the mentioned problem, we were forced to apply an alternative searching methodology. As a preliminary step, we proposed the link between the affiliations reflected in the scientific studies and the entities applicants for patents. We tested this proposal by means of a customized Freedom to Operate Opinion (FTO) requested for a specific optical detection system for high sensitivity label free bioassay. However, only one match was found between paper and patent. In our understanding, this FTO was biased by not including other technologies or the same inclusion criteria, detailed in this review. Hence, we discarded this suggestion.

Taking into consideration this bias, we performed a second search on Google Patents, following the meticulous methodology already used in other studies [44], which involved investigating the main authors of the selected papers (more than 350 authors). At least the first author and the last author were screened to the linked article and registered patent. In the case of not matching, other authors were studied trying to find coincidences between authors and inventors.

Once we had gathered all the potentially linked patents, two researchers (MJP and RLE) reviewed them to ensure that the methodologies, devices, or systems described in the patents were directly related to the PoC devices from the scientific publications. Such relation was considered only when the patents claimed for at least a part of the PoC device (i.e., the biosensor, the read-out methodology, the optical setup…). After cross-check, any disagreement in the study selection was resolved by consulting a third researcher (MHB).

Then, we classified the patents following the same methodology than for the scientific publications. We tried to locate in the claims of the patents the read-out techniques, the optical interrogation technique, the excitation monitoring, the detecting techniques, and the bio application.

Valuing individually all the patents registered by each author enabled us to ascertain a relationship between the patent and the published paper, and henceforth, to calculate the IoTT. Pursuing the quality of methodology, and hence of the results obtained, we conducted strategies to limit biases and random errors. These strategies can be summarized as: an exhaustive search of all relevant articles and patents, reproducible and explicit selection criteria, evaluation of the design and characteristics of the studies, and synthesis and interpretation of the results.

The last step before the calculation of the IoTT was the analysis of the information. This step involved the analysis and mapping out of all the information procured from the previous step.

#### 2.2.3. IoTT Calculation

We defined the index of Transference technology as the relationship between the patent and the published paper. Therefore, the sample frame construction is vital for addressing the issue [45]. On this review, the result of the article search strategy (i.e., the list of included articles) was considered the sampling frame. In addition, the *IoTT* index was calculated with the number of linked patents, according to the following Equation (2):

(2) $$IoTT=100\;\times \;\frac{Registered\;involved\;patents}{Scientific\;publications\;\left(sample\;frame\right)}\left(\%\right)$$

## 3. Results

We performed the already described methodology with an explicit method mainly designed to answer a concrete question. As result, this methodology offered us a technology transfer indicator for the Optical PoC.

**Figure 15:**
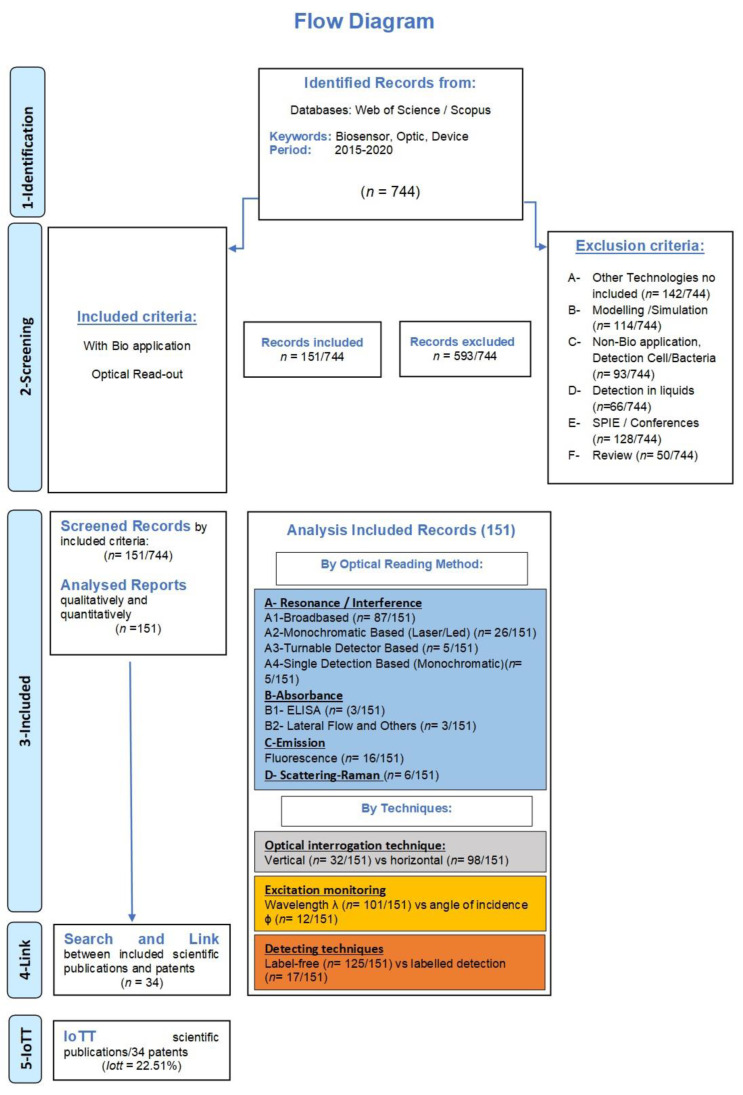
Flow diagram of the literature search illustrates the meticulous screening work in four steps, from the identification of 744 records to the study in depth of 151 scientific articles and the link to the registered patent. (Flow diagram developed from the one described in [46] and particularized for the current review).

For descriptive purposes, we present Figure 15 which summarizes the results of the methodologic process in the different phases from the structured question and prior research protocol to the estimation of the IoTT. These results will be described now.

### 3.1. Results of Scientific Publications

As mentioned above, we applied a methodology that implied the use of explicit methods to identify, select and exclude articles to extract and analyse data from the studies that were included in the review. To facilitate the understanding of the results, the list of publications are gathered in the Supplementary Information organized as follows: (i) the related articles found after applying the article search strategy are listed in Table S1 in Supplementary Materials (list of included and excluded articles are 744); (ii) records excluded for different reasons are listed in Table S2 (593/744); and (iii) the included articles in this review are listed in Table S3 (151/744).

As shown in Figure 15, a total of 744 publications were extracted from databases (Web of Science/Scopus) by considering the keywords and the inclusion criteria. These publications were identified as potentially eligible for inclusion during the first phase of identification by applying the search strategy described. After non considering duplicates, the list of 744 publications was screened and the exclusion criteria were applied. A total of 593 studies were excluded (79.70%). These articles were rejected according to the following: 142 publications were out of our scope as they used other technologies not specified in our inclusion criteria; 114 were theoretical studies; 93 studies had no bio application; 66 papers were about liquids, gas, or detection cells or bacteria, 128 papers were conferences or SPIE publications, and finally, 50 papers were reviews.

Considering that sample frame construction is vital for addressing the issue on this review [47], 151 articles were included for analyses (Table S3) of the total of 744 (20.29%). Understanding the 151 scientific articles as a 100% (sampling frame), we classified them into the already described categories prior proceeding with the next steps of the methodology, as it will be described below.

#### Classification of the Scientific Articles

It is noteworthy that the striking reduction in usable articles from 744 to 151 is because, in the search, most of the registries did not meet all the eligibility criteria we had previously defined.

The quality of the studies included in our review is satisfactory. The main shortcomings to classify the devices into categories were the omission in the description of the techniques for sensors excitation (Wavelength interrogation/Angle of incidence) and omission in the description of the optical interrogation (Vertical/Horizontal). However, as articles somehow specified the optical detection method (interference–resonance, absorbance, emission–fluorescence, and scattering-Raman–SERS), we could extrapolate and ascertain some of the omitted data. Moreover, it is important to mention that a high number of articles could be included in several categories, as they combined different techniques to perform the detecting device. Nevertheless, we classified them into the main described category.

The publications used to specify the improvements or advances that their device suggests, and almost all of them detailed the bio application, or pathology in which they are focused.

**Figure 16:**
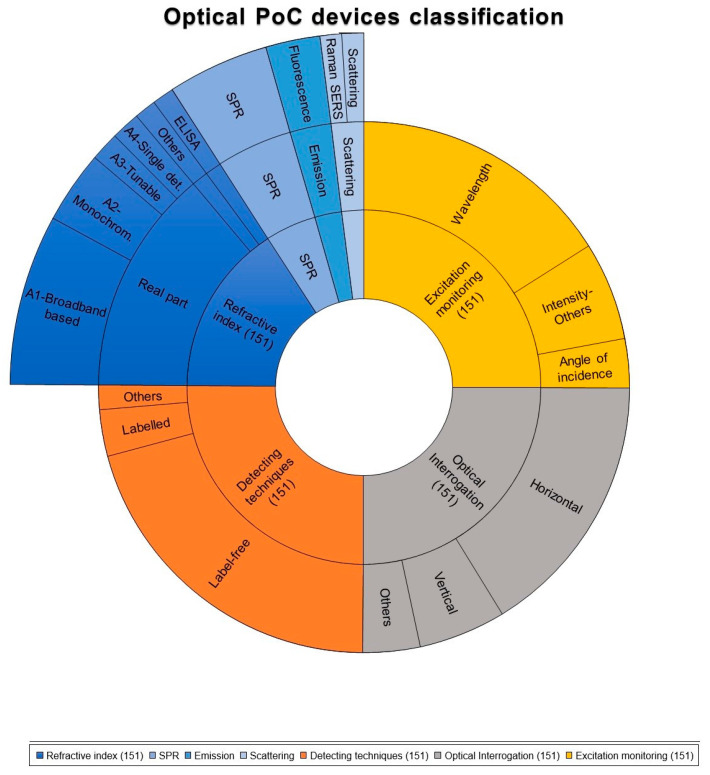
Classification of articles by the categories specified: variations on the refractive index (Real part, Imaginary Part, Emission and Scattering), the optical interrogation technique (Vertical/Horizontal), the excitation monitoring (Wavelength/Angel of incidence) and detecting techniques of molecular interactions (Label-free/Labelled).

Figure 16 shows the classification of our sampling frame according to the detecting techniques (i.e., label-free vs. labelled detection); the excitation monitoring (i.e., wavelength λ vs. angle of incidence ϕ); the optical interrogation technique (i.e., vertical vs. horizontal); and the variation of the RI.

Following the described methodology, the first category in which we classified the Optical PoC devices reported were the detecting techniques (i.e., labelled vs. label-free techniques). Significantly, the advantages of the label-free systems in terms of reducing costs, time for results, and the complexity of the diagnostic were reflected in the higher number of PoC devices based on label-free methods. The label-free PoC devices studied reported the simplification of the process by reducing the washing steps and additions of reagents [48]. Apart from that, they claimed for higher quality and resolution detectability, with more information content and fewer false negatives, as compared to labelled biosensors [49]. In this context, the number of PoC devices based on label-free methods represented the 83% of the publications studied (125/151) while the labelled detection PoCs were described in a 11% of the screened articles (17/151). Apart from that, nine publications did not clearly specify whether the method was labelled or label-free, and the 6% (9/151) was classified as “others”. See Figure 16 and references from Table 1.

Considering the excitation monitoring, we classified the 67% of the publications into the wavelength interrogation category (101/151), the 8% corresponded to the angle of incidence interrogation (12/151), and the 25% were included into the “others or not specified” techniques (38/151). See Figure 16 and references from Table 1.

Referring to the optical interrogation technique, the use of vertically interrogated devices may imply less complex optical coupling and can also have advantages from the fluidic and biofunctionalization point of view, as it was reported in the review related to interferometric devices [50]. The vertical optical interrogation covers the 21% of the papers studied in this review (32/151), while the 65% referred to horizontal optical interrogation methods (98/151). Other optical interrogation configurations or not detailed technique represents the 14% of the papers studied on this review (21/151). See Figure 16 and references from Table 1.

**Table 1:** Classification of the Scientific Publications included in the study according to their categories and their references.

Classification	Sub-Field		List of References
**Detecting techniques**	**Labelled** **(11%)**		**[51,52,53,54,55,56,57,58,59,60,61,62,63,64,65,66,67]**
	**Label-free** **(83%)**		**[40,44,68,69,70,71,72,73,74,75,76,77,78,79,80,81,82,83,84,85,86,87,88,89,90,91,92,93,94,95,96,97,98,99,100,101,102,103,104,105,106,107,108,109,110,111,112,113,114,115,116,117,118,119,120,121,122,123,124,125,126,127,128,129,130,131,132,133,134,135,136,137,138,139,140,141,142,143,144,145,146,147,148,149,150,151,152,153,154,155,156,157,158,159,160,161,162,163,164,165,166,167,168,169,170,171,172,173,174,175,176,177,178,179,180,181,182,183,184,185,186,187,188,189,190,191,192,193]**
	**Others** **(6%)**		**[194,195,196,197,198,199,200,201,202]**
**Excitation monitoring** [138]	**Angle of incidence** **(8%)**		**[54,57,69,73,81,99,109,119,120,150,169,203]**
	**Wavelength** **(67%)**		**[40,44,55,58,59,60,61,62,67,70,71,72,74,75,76,78,79,80,82,84,85,88,89,90,92,93,94,95,96,97,99,101,102,103,105,106,108,111,112,114,115,116,117,118,122,123,124,125,127,128,129,133,134,135,136,137,138,140,141,142,143,145,146,147,149,150,152,153,154,155,156,157,158,159,160,164,167,168,172,175,176,177,178,179,181,182,184,185,186,187,191,192,196,199,200,202,204,205,206]**
	**Intensity—Others or** **not specified** **(25%)**		**[51,52,53,56,63,65,68,83,86,87,91,104,107,110,121,130,131,132,144,148,161,162,163,165,170,171,174,180,183,190,194,195,197,198,201]**
**Optical Interrogation**	**Vertical** **(21%)**		**[51,54,55,57,63,65,68,83,87,89,96,98,107,109,113,119,122,125,128,139,143,145,146,148,150,163,171,174,185]**
	**Horizontal** **(65%)**		**[42,45,53,60,61,63,68,70,71,72,73,74,75,76,77,78,79,80,81,82,83,85,86,87,89,90,91,93,94,95,96,98,100,101,102,103,104,105,107,109,111,112,113,115,116,117,118,119,121,122,124,125,128,130,131,132,134,135,136,137,138,139,141,142,143,145,148,150,152,153,154,158,159,160,161,162,163,165,166,168,169,170,171,173,176,177,178,179,183,185,187,188,191,192,193,194]**
	**Others** **(14%)**		**[53,56,58,91,105,132,154,155,156,179,180,183,194,195,196,197,198,199,200,201,202]**
**Refractive index**	**Real part**	**A1—Broadband-based** **(58%)**	**[40,44,57,68,69,70,71,72,73,74,75,76,78,79,80,81,82,85,88,89,90,91,92,93,94,95,98,99,101,102,103,105,106,109,110,111,112,113,115,116,119,122,123,124,125,127,129,130,133,134,136,137,138,140,141,142,145,146,147,149,150,152,153,154,155,157,159,161,162,164,167,168,172,175,181,183,185,186,191,192,194,195,196,197,200,205]**
		**A2—Monochromatic-based (17%)**	**[59,77,84,86,87,96,100,107,114,120,128,132,135,151,156,165,169,174,176,178,179,180,183,187,190,198]**
		**A3—Turnable (3%)**	**[53,117,160,177,184]**
		**A4—Single detection method (3%)**	**[83,104,108,131,171]**
	**Imaginary part**	**ELISA** **(2%)**	**[52,55]**
	**Others**	**Ellipsometry… (2%)**	**[121,148,170]**
**SPR** **(28%)**	**SPR**	**A1—Broadband-based**	**[70,71,73,76,79,80,81,83,84,85,88,90,91,95,101,106,116,120,123,127,129,130,134,135,138,140,145,146,160,164,172,174,175,180,182,185,190,193,199,201,205]**
**Emission**	**Fluorescence (11%)**		**[54,56,58,60,61,62,63,65,139,143,158,163,201,202]**
**Scattering**	**Scattering (2%)**		**[51,193,199]**
**Scattering**	**Raman SERS (4%)**		**[67,97,118]**

A more detailed attention required the classification according to the variation in the RI, as sometimes it was not very clearly described in the publications. First, based on the monitored changes in the real part of the refractive index, the deep study performed with the publications related to Optical PoCs led to the inclusion of many of them in the A1 category, as many of them used a broadband source of light, and they embody 58% (87/151). Monochromatic technology was widely researched and makes up 17% (26/151) of the included articles. Concerning the detector features, A3—Turnable Detector Based devices represent 3% (5/151). Conversely, the A4—Single Detection Based was found only in five of the scientific publications studied (5/151), being 3% of the total. The SPR technique was found in 42 articles (42/151), corresponding to 28% of the total. Concretely, the SPR was significant in the A1 category, thus, it is depicted only within this category (see Figure 16 and references from Table 1).

Second, based on the imaginary part of the RI, we considered B1—ELISA and B2—Lateral Flow as the most representative techniques. Contrary to expectations, the scientific works based on those techniques only represented 2% (3/151). Some articles claimed for the use of the absorbance technique, but it was not specified whether they used ELISA or Lateral Flow techniques, thus, these articles were classified as “Others” and they represent 2% (3/151). See Figure 16 and references from Table 1.

Third, concerning to fluorescence the 11% (16/151) of the reports were included in this category. See Figure 16 and references from Table 1.

Finally, among the PoCs devices related to the Raman technology, they were reported as SERS-based technology by providing a label-free and non-invasive method to measure the inelastic scattered light. Concisely, the devices described in Table 1, referred to the vibration eigenmodes of the excited molecules. We classified the 4% (6/151) of the publications into the Raman/Scattering technology.

As it can be seen in Figure 16 and Table 1, the most prevalent technical characteristic of the Optical PoC devices described in the literature is the label-free detecting technique. The predominant optical interrogation methodology is the horizontal setup. Moreover, the wavelength seems to be the mainly excitation monitoring used. Finally, considering the particular classification based on the variation on the RI, the A1 category, that is, the Broadband-based method is the most widely used in the articles reviewed.

### 3.2. Results of Patents

As detailed in the methodology section, our research entails the procurement of optical PoC devices via different sources. Initially, we searched registered patents between 2015 and 2020, using the keywords in Google Patents. After the difficulties discussed in the methodology section in detail, which obstructed knowing the transfer index between scientific articles and registered patents, we were forced to implement a new methodology that involved investigating the main authors of the 151 included studies on Google patents to analyse all the patents registered by each author. The final list of patents linked to the scientific publications comprised 34 registered patents.

Regarding the technology detailed in the patents, as we mentioned in the Materials and Methods Section, the language used in the patents is characterized by describing the concepts in a very general way to obtain the widest possible scope of patent protection. Considering the intention of the inventors to protect the technology, the main shortcomings found was the lack of constriction on the description of the techniques patented.

**Figure 17:**
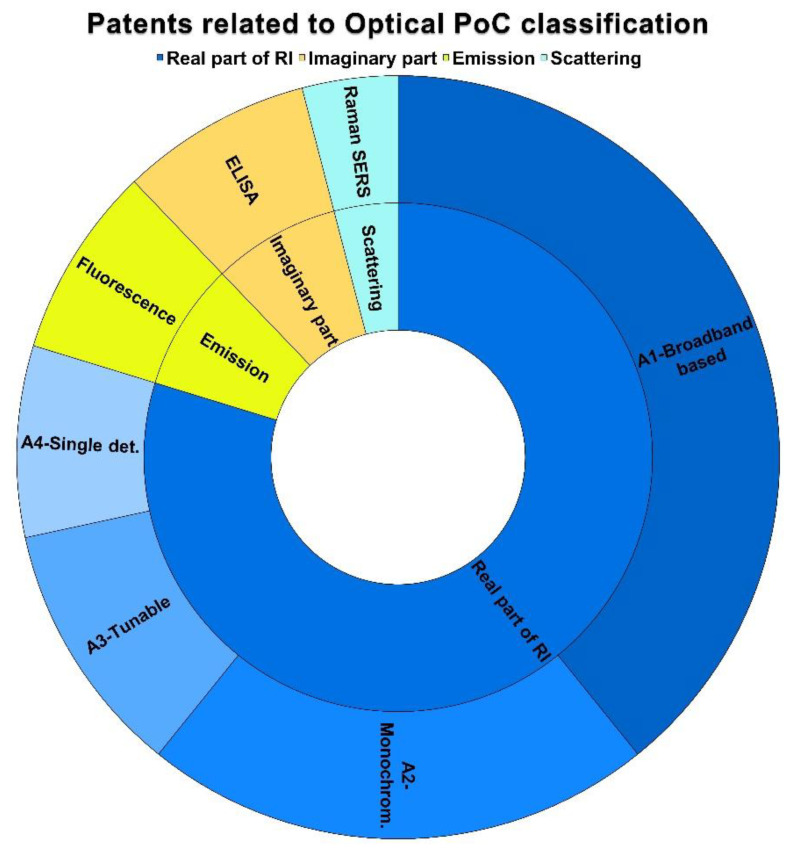
Classification of the linked patents.

Therefore, we were only able to classify them into the categories related to the variation of the RI. The patents related to the variation of the real part of the RI represents 78% of the linked patents. They were subclassified as can be seen in Figure 17 and Table 1 and Table 2, that is the A1—Broadband-based category represents 38% (13/34), the A2—Monochromatic-based corresponds to 22% (8/34), 11% (4/34) is embodied by the A3—Turnable Detector category, and finally, the A4—Single Detection method represents 3% (1/34).

The category based on the variation of the imaginary part of the RI was found in 8% of the linked patents (3/34), restricted to the B1—ELISA technique. Meanwhile, the Emission–Fluorescence, and the Scattering–Raman categories were found in 8 and 6%, respectively (i.e., in [3/34] and [2/34] of the linked patents).

### 3.3. IoTT Result

Our search methodology applied for scientific publications provided 151 reports fulfilling the inclusion–exclusion criteria. Then, the finally applied patent search strategy allowed us to cross-link authors with inventors, and 34 patents were related to the scientific works. Therefore, following the IoTT calculation formula (Equation (1)), we obtained the indicator which relates scientific work with the already patented one, that is, the IoTT, with a value of 22.51%. The Table S4 gathers the connection between the DOI of the scientific publications and the EP Register.

What is interesting in these data is that we were also able to calculate the IoTT of the categories related to the variation of the RI. A1—Broadband-based category represents 15% (13/87), the A2—Monochromatic-based corresponds to 21% (8/26), 60% (3/5) is embodied by the A3—Turnable Detector category, and finally, the A4—Single Detection method represents 20% (1/5).

The category based on the variation of the imaginary part of the RI was found in 100% of the linked patents (3/3), restricted to the B1—ELISA technique. Meanwhile, the Emission–Fluorescence, and the Scattering–Raman categories were found in 19% and 33%, respectively (i.e., in [3/16] and [2/6] of the linked patents).

These results supported the fact that there is an enormous unmet potential that has not been exploited in the market (77.49%). This is proven by a long list of laboratory prototypes described in the literature which have never been previously patented, neither transferred into an authentic clinical scenario. Our results revealed the lack of transferability of the knowledge to the industrial and technological environment.

Likewise, the number of patents not coming from scientific published articles showed the existence of another environment where industrial developments are made. Hence, the leap to the market without a prior patent is also a possibility, although it is not a common practice for a researcher.

## 4. Discussion

To the best of our knowledge, there are no reports for the relative comparison of the technological advances described in the scientific works to registered patents. Therefore, the current review set the baseline to assess the impact of evaluating such relationship by developing a methodology to obtain an indicator to quantify it, our IoTT.

Our aim was to cover gap found on that field. A reasonable approach to tackle this issue was to identify only those scientific articles related to Optical Reading Devices as PoC systems. We performed a classification according to some predefined categories and were able to have a representative sample of scientific articles concretely classified.

Once we had such a bounded sample to estimate the IoTT, we strike a balance between the advances in the scientific literature and the patented technology. However, the intrinsic protective intention of the industrial property protection led to the reformulation of the methodology initially proposed to find the desired match between science and technology transference. Finally, we crosslinked science and technology transfer, we checked the underlying technology and evaluated the result obtained.

## 5. Conclusions

Despite the ubiquitous knowledge of the gap between the laboratory prototype and a marketable product, no published work was found to relate scientific reports and registered patents. The methodology reported in this article is valid to estimate a transference index in areas of interest and the definition of the areas of interest must be filtered with exquisite care to obtain a representative and precise indicator. Although considering that the sample size of each study is not infinite, and therefore there may be sampling error, and the observed effects do not have to be exact to the effect if we studied the population, not the sample.

Despite this limitation, and although we could have chosen other keywords or even other search tools, due to the relative nature of the study in the observed scientific field as defined by the chosen keywords, this reported methodology guarantees to obtain a reliable indicator of technology transfer in this field of optical PoC devices and it paves the way for future study, which is highly recommended for any other technology in any sector where it is relevant to know the transfer rate between Science and the Market.

As an important aspect of this review, the interaction among Science, Technology, and Patent have been studied to estimate the IoTT. After laborious filtering of sources, we focused on 151 reports and found 34 related patents. Therefore, the conclusion was that considering the 151 scientific articles as the sampling frame of 100%, the interaction between science and registered technology could be quantified with an IoTT of 22.51% for the 34 patents.

Finally, this methodology and the results obtained from the bounded analysis of this study led us to the conclusion that technology researched in the laboratory and registered by a patent is the type A1—Broadband-based, representing 58% and 42%, respectively. Likewise, findings from this review can help target current issues and identify future specific research needs, and it will be the subject of development for further articles. These studies will allow us to describe the landscape of patented PoCs and their impact on the market through their commercialization.

## Figures and Tables

**Table 2:** Classification of the Patents included in the study according to their categories and their references.

Classification	List of References	
**Refractive** **index**	A1—Broadband-based (38%)	[207,208,209,210,211,212,213,214,215,216,217,218,219,220,221,222,223]
	A2—Monochromatic-based (22%)	[224,225,226,227,228,229,230,231]
	A3—Turnable detector-based (11%)	[232,233,234,235,236]
	A4—Single detection method (3%)	[237]
	ELISA (8%)	[238,239,240]
	Fluorescence (8%)	[241,242]
	Scattering (3%)	[243]
	Raman SERS (3%)	[244]

## Data Availability

Datasets generated during and/or analyzed during the current study are available in the Supplementary Materials or available from the corresponding author on reasonable request.

## Supplementary Materials

The following supporting information can be downloaded at https://www.mdpi.com/article/10.3390/bios12121091/s1 Table S1: List of articles included in the first search (744); Table S2: List of excluded articles (593); Table S3: List of included articles (151); Table S4: List that links DOI and EP Register (34).
