# Supplementary material for: A review of Optical Point-of-Care devices to Estimate the Technology Transfer of These Cutting-Edge Technologies
Source: Biosensors (Basel). 2022 Nov 29;12(12):1091. doi: 10.3390/bios12121091 (PMC9776401; doi:10.3390/bios12121091)
Supplement: Supplementary file 1 [file biosensors-12-01091-s001.zip › Table S1 Suppl_File.pdf]

APPENDIX A_ LIST OF ARTICLES INCLUDED		
№	TITLE_SCIENTIFIC PUBLICATION	DIGITAL OBJECT IDENTIFIER (DOI)
1	Optical fiber bio-sensor for phospholipase using liquid crystal	10.1016/j.bios.2020.112547
2	Real-time interrogation of fiber optic biosensor using TiO2 coated etched long-period grating	10.1063/5.0020571
3	The potential of terahertz sensing for cancer diagnosis	10.1016/j.heliyon.2020.e05623
4	Recent Advances in the Fabrication and Application of Graphene Microfluidic Sensors	10.3390/mi11121059
5	Design and analysis of electro-optic modulators based on high contrast gratings in AlGaN/GaN heterostructures	10.1088/1361-6641/abbc8d
6	Modelling and simulation of novel liquid-infiltrated PCF biosensor in Terahertz frequencies	10.1049/iet-opt.2020.0069
7	Etched multicore fiber sensor using copper oxide and gold nanoparticles decorated graphene oxide structure for cancer cells detection	10.1016/j.bios.2020.112557
8	A switchable split ring resonator nanoantenna design with organic material composite as a refractive index sensor	10.1016/j.optcom.2020.126211
9	Sensitivity enhancement of WS2-coated SPR-based optical fiber biosensor for detecting glucose concentration	10.1117/12.2026061
10	Detection of cardiac troponin-I by optic biosensors with immobilized anti-cardiac troponin-I monoclonal antibody	10.1016/j.talanta.2020.121259
11	Recent advance in biosensing applications based on two-dimensional transition metal oxide nanomaterials	10.1016/j.talanta.2020.121308
12	Label-free biosensor array comprised of Vernier microring resonator and 3x3 optical coupler	10.1140/epjp/s13360-020-00888-6
13	Numerical analysis of Bragg grating-based slot-micro-ring coupling resonator system for electromagnetically-induced transparency-like effect	10.1007/s11082-020-02555-7
14	The Measurement of Nanoparticle Concentrations by the Method of Microcavity Mode Broadening Rate	10.3390/s20205950
15	All fiber, highly sensitive sensor based on gold nanoparticle-coated macrobent single mode fiber for human temperature monitoring	10.1117/1.JNP.14.046013
16	Design of D-Shaped PCF-SPR sensor with dual coating of ITO and ZnO conducting metal oxide	10.1016/j.ijleo.2020.165135
17	CMOS-Compatible Silicon Photonic Sensor for Refractive Index Sensing Using Local Back-Side Release	10.1109/LPT.2020.3019114
18	Polymer-Coated Fiber Optic Sensor as a Process Analytical Tool for Biopharmaceutical Impurity Detection	10.1109/TIM.2020.2981982
19	Plasmonic Sensors for Monitoring Biological and Chemical Threat Agents	10.3390/bios10100142
20	Detection of L-Cysteine Using Silver Nanoparticles and Graphene Oxide Immobilized Tapered SMS Optical Fiber Structure	10.1109/JSEN.2020.2997690
21	Reflector-less nanoparticles doped optical fiber biosensor for the detection of Case thrombin	10.1016/j.bios.2020.112365
22	Numerical analysis of gold coating based quasi D-shape dual core PCF SPR sensor	10.1007/s11082-020-02555-7
23	Nanomaterial-Enhanced Fiber Optofluidic Laser Biosensor for Sensitive Enzyme Detection	10.1109/JLT.2020.2997993
24	Behaviour of Poynting vector for dielectric-metal-dielectric optical waveguides and applications	10.1007/s11082-020-02519-x
25	D-type photonic crystal fiber sensor based on metal nanowire array	10.1016/j.ijleo.2020.165010

№	TITLE_SCIENTIFIC PUBLICATION	DIGITAL OBJECT IDENTIFIER (DOI)
26	Highly Sensitive D-Shaped Optical Fiber Surface Plasmon Resonance Refractive Index Sensor Based on Ag-alpha-Fe2O3 Grating	10.1109/JSEN.2020.2992854
27	Optically Tunable Triple-Band Perfect Absorber for Nonlinear Optical Liquids Sensing	10.1109/JSEN.2020.2989742
28	3-D Printed Instrumentation for Point-of-Use Leaky Waveguide Biochemical Sensor	10.1109/TIM.2020.2969036
29	All-Dielectric Metasurface Fluorescence Biosensors for High-Sensitivity Antibody/Antigen Detection	10.1021/acsnano.0c07722
30	Fiber optic particle plasmon resonance immunosensor for rapid and sensitive detection of methamphetamine based on competitive inhibition	10.1016/j.microc.2020.105026
31	A Plasmonic Nano-Biosensor Based on Two Consecutive Disk Resonators and Unidirectional Reflectionless Propagation Effect	10.1109/JSEN.2020.2987319
32	Theoretical Analysis of a Simultaneous Graphene-Based Circular Plasmonic Refractive Index and Thickness Bio-Sensor	10.1109/JSEN.2020.2987696
33	Ultrasensitive optical biosensors based on microresonators with bent waveguides	10.1016/j.ijleo.2020.164906
34	An Integrated, Optofluidic System With Aligned Optical Waveguides, Microlenses, and Coupling Prisms for Fluorescence Sensing	10.1109/JMEMS.2020.3004374
35	Two-photon fluorescence-assisted laser ablation of non-planar metal surfaces: fabrication of optical apertures on tapered fibers for optical neural interfaces	10.1364/OE.395187
36	Polarization Selectivity of the Thin-Metal-Film Plasmon-Assisted Fiber-Optic Polarizer	10.1021/acsami.0c08274
37	Numerical analysis of effective refractive index bio-sensor based on graphene-embedded slot-based dual-micro-ring resonator	10.1142/S0217979220501453
38	Hollow-core graded index optical fiber refractive index sensor based on surface plasmon resonance	10.1007/s11082-020-02461-y
39	Hollow core photonic crystal fiber-assisted Raman spectroscopy as a tool for the detection of Alzheimer's disease biomarkers	10.1117/1.JBO.25.7.077001
40	Design and Realization of a Novel Poly-Silicon Light-Emitting Device Based on Standard CMOS Technology	10.3788/CJL202047.0701027
41	Highly sensitive fiber optic surface plasmon resonance sensor employing 2D nanomaterials	10.1007/s00339-020-03712-1
42	Enhancement of ultrathin localized surface plasmon resonance sensitivity using sequential temperature treatment for liquids sensing	10.1016/j.sna.2020.112006
43	An Analysis of a Compact Label-Free Guiding-Wave Biosensor Based on a Semiconductor-Clad Dielectric Strip Waveguide	10.3390/s20123368
44	Two Modes Excited SPR Sensor Employing Gold-Coated Photonic Crystal Fiber Based on Three-Layers Air-Holes	10.1109/JSEN.2020.2972031
45	Recent advances in fiber-optic evanescent wave sensors for monitoring organic and inorganic pollutants in water	10.1016/j.trac.2020.115892
46	A novel surface plasmon based photonic crystal fiber sensor	10.1007/s11082-020-02403-8
47	Biophotonic sensor for rapid detection of brain lesions using 1D photonic crystal	10.1007/s11082-020-02409-2
48	Urea detection using bio-synthesized gold nanoparticles: an SPR/LSPR based sensing approach realized on optical fiber	10.1007/s11082-020-02405-6
49	A D-Shaped Fiber Long-Range Surface Plasmon Resonance Sensor With High Q-Factor and Temperature Self-Compensation	10.1109/TIM.2019.2920187

№	TITLE_SCIENTIFIC PUBLICATION	DIGITAL OBJECT IDENTIFIER (DOI)
50	Critical assessment of relevant methods in the field of biosensors with direct optical detection based on fibers and waveguides using plasmonic, resonance, and interference effects	10.1109/JLT.2020.2969016
51	Microcapillary-Based Integrated LSPR Device for Refractive Index Detection and Biosensing	10.1109/JLT.2020.2969016
52	Simultaneous optical and electrochemical label-free biosensing with ITO-coated lossy-mode resonance sensor	10.1016/j.bios.2020.112050
53	Au nanoparticles as label-free competitive reporters for sensitivity enhanced fiber-optic SPR heparin sensor	10.1016/j.bios.2020.112039
54	Designing a dual steering wheel microstructured blood components sensor in terahertz wave band	10.1117/1.OE.59.4.047104
55	Development of Uric Acid Biosensor Using Gold Nanoparticles and Graphene Oxide Functionalized Micro-Ball Fiber Sensor Probe	10.1109/TNB.2019.2958891
56	Measurement of MIPs Responses Deposited on Two SPR-POF Sensors Realized by Different Photoresist Buffer Layers	10.1109/TIM.2020.2967864
57	Thermally Stable Optical Filtering Using Silicon-Based Comb-Like Asymmetric Grating for Sensing Applications	10.1109/JSEN.2019.2960604
58	Advancements in SPR biosensing technology: An overview of recent trends in smart layers design, multiplexing concepts, continuous monitoring and in vivo sensing	10.1016/j.aca.2019.12.067
59	Coupling between surface plasmon polariton and planar waveguide modes in the biosensor based on metal-insulator-metal/planar waveguide structure	10.1016/j.optcom.2019.124928
60	The Antibody-Free Recognition of Cancer Cells Using Plasmonic Biosensor Platforms with the Anisotropic Resonant Metasurfaces	10.1016/j.optlastec.2019.105922
61	Highly sensitive sensor based on D-shaped microstructure fiber with hollow core	10.1016/j.optlastec.2019.105922
62	Fiber optic nanogold-linked immunosorbent assay for rapid detection of procalcitonin at femtomolar concentration level	10.1016/j.bios.2019.111871
63	A high figure of merit refractive index sensor based on Fano resonance in all-dielectric metasurface	10.1016/j.rinp.2019.102833
64	Low-Cost and Highly Sensitive Liquid Refractive Index Sensor Based on Polymer Horizontal Slot Waveguide	10.1007/s13320-019-0560-y
65	An Integrated Biological Analysis and Flow Rate Sensing for the Real-Time Detection of Carcinogen in Water Based on Co2+-Doped Optical Fibers	10.1109/JSEN.2019.2948964
66	Study on the sensing characteristics of Fano resonance based on a coupled streamlined resonance cavity	10.1364/OSAC.377612
67	⍰ Performance analysis of graphene-based surface plasmon resonance biosensor for blood glucose and gas detection	10.1007/s00339-020-3328-8
68	Introducing waveguide loss: Another way to realize a high-sensitivity microring biosensor	10.1016/j.ijleo.2019.163339
69	Lossy mode resonance sensors based on nanocoated multimode-coreless-multimode fibre	10.1016/j.snb.2019.126955
70	S-shaped long period fiber grating glucose concentration biosensor based on immobilized glucose oxidase	10.1016/j.ijleo.2019.163960
71	Multi-Channel Photonic Crystal Fiber Based Surface Plasmon Resonance Sensor for Multi-Analyte Sensing	10.1109/JPHOT.2019.2961110

№	TITLE_SCIENTIFIC PUBLICATION	DIGITAL OBJECT IDENTIFIER (DOI)
72	Diphenylalanine Nanotube Coated Fiber Bragg Grating for Methanol Vapor Detection	10.1109/JSEN.2019.2946123
73	?<br>Dual-polarized optical sensing of microstructure fiber with pentagonal-lattice based on surface plasmon resonance in the near-IR spectrum	10.1016/j.ijleo.2019.163671
74	?<br>Optical properties of fluidic defect states in one-dimensional graphene-based photonic crystal biosensors: visible and infrared Hall regime sensing	10.1140/epjp/s13360-019-00056-5
75	General formulae of designing novel photonic digital sensors with arbitrary levels of directional couplers for testing liquids	10.1007/s11082-019-2177-1
76	Reflectance aptasensor based on metal salphen label for rapid and facile determination of insulin	10.1016/j.talanta.2019.120321
77	The interaction mechanism between bovine serum albumin and single-walled carbon nanotubes depending on their diameter and concentration in solid bionanocomposites	10.1117/12.2554935
78	Fiber Optic Biosensors: Types, Optical Parameters, Applications and Future Scope	10.1063/5.0001791
79	Recent Advancements In Fiber-Optics Biosensors	10.1063/5.0001245
80	Self-assembled glass-based Fano resonant metasurfaces	10.1117/12.2546112
81	?<br>Synthesis of TiO2 nanoparticles decorated with gold nanoclusters using pulsed laser ablation in liquid	10.1070/QEL17318
82	Optimally designed narrowband guided-mode resonance transmittance filters for label-free optical biosensor	10.1117/12.2545941
83	Versatile Bio-Organism Detection using Microspheres for Future Biodegradation and Bioremediation Studies	10.1117/12.2543800
84	Fabrication of multichannel Bloch long range surface plasmon biosensors	10.1117/12.2545106
85	Low-cost glucose biosensor fabricated by a photosensitive resin that features nanoparticles	10.1117/12.2546938
86	Bioengineered Polymer/Composites as Advanced 'Biological Detection Sorbitol: An Application in Healthcare Sector	10.2174/1568026620666200306131416
87	Sensitivity Enhancement in Long Period Gratings by Mode Transition in Uncoated Double Cladding Fibers	10.1016/j.sna.2013.01.012
88	Multiplexed Remote SPR Detection of Biological Interactions through Optical Fiber Bundles	10.3390/s20020511
89	MoSe2-Au Based Sensitivity Enhanced Optical Fiber Surface Plasmon Resonance Biosensor for Detection of Goat-Anti-Rabbit IgG	10.1109/ACCESS.2019.2961751
90	Modulation of localized surface plasmon resonance of the titanium nitride nanoparticle array based on graphene	10.1016/j.ijleo.2019.163420
91	A Compact Biosensor for Binding Kinetics Analysis of Protein-Protein Interaction	10.1109/JSEN.2019.2938655
92	Functionalized etched tilted fiber Bragg grating aptasensor for label-free protein detection	10.1016/j.bios.2019.111765
93	A novel optical fiber glucose biosensor based on carbon quantum dots-glucose oxidase/cellulose acetate complex sensitive film	10.1016/j.bios.2019.111760

Nº	TITLE_SCIENTIFIC PUBLICATION	DIGITAL OBJECT IDENTIFIER (DOI)
94	Quantitative remote and on-site Hg2+ detection using the handheld smartphone based optical fiber fluorescence sensor (SOFFS)	10.1016/j.snb.2019.127168
95	? Dual-polarized ultrahigh sensitive gold/MoS2/graphene based D-shaped PCF refractive index sensor in visible to near-IR region	10.1007/s11082-019-2122-3
96	Highly sensitive and selective localized surface plasmon resonance biosensor for detecting glutamate realized on optical fiber substrate using gold nanoparticles	10.1016/j.photonics.2019.100730
97	Design and investigation of PCF-based blood components sensor in terahertz regime	10.1007/s00339-019-3164-x
98	Ultra-sensitive photonic crystal cancer cells sensor with a high-quality factor	10.1016/j.cryogenics.2019.102991
99	Ultrasensitive biosensor based on magnetic microspheres enhanced microfiber interferometer	/10.1016/j.bios.2019.111563
100	Ultrasensitive biosensor based on magnetic microspheres enhanced microfiber interferometer	10.1016/j.bios.2019.111563
101	Size dependent plasmonic properties of Ga@Ag & Cs@Ag liquid-metal nanospheres	10.1016/j.optcom.2019.07.049
102	Design of a High-Resolution Metal-Insulator-Metal Plasmonic Refractive Index Sensor Based on a Ring-Shaped Si Resonator	10.1007/s11468-019-00926-9
103	Design and Performance of a Multipurpose 2-D Photonic Crystal Device Based on Y Couplers	10.1007/s11468-019-00934-9
104	Circular-Pattern Photonic Crystal Fiber for Different Liquids with High Effective Area and Sensitivity	10.1007/s11468-019-00977-y
105	Metal Nanowire Assisted Hollow Core Fiber Sensor for an Efficient Detection of Small Refractive Index Change of Measurand Liquid	10.1007/s11468-019-00969-y
106	Development of an enzyme-based fiber optic biosensor for detection of haloalkanes	10.1108/SR-01-2019-0001]
107	Demonstration of an optical biosensor for the detection of faecal indicator bacteria in freshwater and coastal bathing areas	10.1007/s00216-019-02182-6
108	Plasmonic Diffraction Field Pattern Imaging Could Resolve Ultrasensitive Bioinformation	10.1021/acsphotonics.9b01076
109	Surface-enhanced Raman scattering-active photonic crystal fiber probe: Towards next generation liquid biopsy sensor with ultra high sensitivity	10.1002/jbio.201900027
110	A highly selective LSPR biosensor for the detection of taurine realized on optical fiber substrate and gold nanoparticles	10.1016/j.yofte.2019.101962
111	Highly sensitive label-free in vitro detection of aflatoxin B1 in an aptamer assay using optical planar waveguide operating as a polarization interferometer	10.1007/s00216-019-02033-4
112	A Review: Evolution and Diversity of Optical Fibre Plasmonic Sensors	10.3390/s19224874
113	Optical biosensors based on refractometric sensing schemes: A review	10.1016/j.bios.2019.111693

№	TITLE_SCIENTIFIC PUBLICATION	DIGITAL OBJECT IDENTIFIER (DOI)
114	The application of porous Si photonic crystals for metal-resonance enhanced fluorescence	10.1007/s11801-019-9035-z
115	Vertical silicon nanowire-based racetrack resonator optical sensor	10.1007/s00339-019-2942-9
116	An Analytical Approach to Predict Maximal Sensitivity of Microring Resonators for Absorption Spectroscopy	10.1109/JLT.2019.2938040
117	Slow light enhanced bio sensing properties of silicon sensors	10.1007/s11082-019-2067-6
118	Biosensors for early diagnosis of pancreatic cancer: a review	10.1016/j.trsl.2019.08.002
119	Sensitivity enhanced D-type large-core fiber SPR sensor based on Gold nanoparticle/Au film co-modification	10.1016/j.optcom.2019.06.026
120	Fiber optic sensor based on ZnO nanowires decorated by Au nanoparticles for improved plasmonic biosensor	10.1038/s41598-019-52056-1
121	Smart-phone, paper-based fluorescent sensor for ultra-low inorganic phosphate detection in environmental samples	10.1038/s41378-019-0096-8
122	Full integration of photonic nanoimmunosensors in portable platforms for on-line monitoring of ocean pollutants	10.1016/j.snb.2019.126758
123	Plasmonic-3D photonic crystals microchip for surface enhanced Raman spectroscopy	10.1016/j.bios.2019.111596
124	Polariton nanophotonics using phase-change materials	10.1038/s41467-019-12439-4
125	High-Performance Label-Free Near-Infrared SPR Sensor for Wide Range of Gases and Biomolecules Based on Graphene-Gold Grating	10.1007/s11468-019-00906-z
126	Tetra-core surface plasmon resonance based biosensor for alcohol sensing	10.1016/j.physb.2019.05.047
127	Enhancing the evanescent field in TiO2/Au hybrid thin films creates a highly sensitive room-temperature formaldehyde gas biosensor	10.1016/j.colsurfb.2019.06.033
128	Wearable flexible sweat sensors for healthcare monitoring: a review	10.1098/rsif.2019.0217
129	Surface plasmon resonance (SPR) biosensors for food allergen detection in food matrices	10.1016/j.bios.2019.111449
130	Photonic crystal slab biosensors fabricated with helium ion lithography (HIL)	10.1016/j.sna.2019.07.017
131	Optical Interrogation Techniques for Nanophotonic Biochemical Sensors	10.3390/s19194287
132	Non-enzymatic D-glucose plasmonic optical fiber grating biosensor	10.1016/j.bios.2019.111506
133	Current status of optical fiber biosensor based on surface plasmon resonance	10.1016/j.bios.2019.111505
134	Graphene Nanoribbon Assisted Refractometer Based Biosensor for Mid-Infrared Label-Free Analysis	10.1007/s11468-019-00909-w
135	Highly Sensitive Dual-Core PCF Based Plasmonic Refractive Index Sensor for Low Refractive Index Detection	10.1109/JPHOT.2019.2931713
136	Water-Induced Fused Silica Glass Surface Alterations Monitored Using Long-Period Fiber Gratings	10.1109/JLT.2019.2909947

№	TITLE_SCIENTIFIC PUBLICATION	DIGITAL OBJECT IDENTIFIER (DOI)
137	High-sensitivity ultra-quality factor and remarkable compact blood components biomedical sensor based on nanocavity coupled photonic crystal	10.1016/j.rinp.2019.102478
138	Sensitivity comparison of surface plasmon resonance (SPR) and magneto-optic SPR biosensors	10.1140/epjp/i2019-12819-3
139	Refractive index sensitivity enhancement of optical fiber SPR sensor utilizing layer of MWCNT/PtNPs composite	10.1016/j.yofte.2019.05.007
140	Fast fluorometric enumeration of E. coli using passive chip	10.1016/j.mimet.2019.105680
141	Computational Study of Photonic Crystal Resonator for Biosensor Application	10.1515/freq-2019-0025
142	A Bimetallic-Coated, Low Propagation Loss, Photonic Crystal Fiber Based Plasmonic Refractive Index Sensor	10.3390/s19173794
143	Optimizing the Limit of Detection of Waveguide-Based Interferometric Biosensor Devices	10.3390/s19173671
144	Bio-Recognition in Spectroscopy-Based Biosensors for *Heavy Metals-Water and Waterborne Contamination Analysis	10.3390/bios9030096
145	A New Device Based on Interferometric Optical Detection Method for Label-Free Screening of C-Reactive Protein	10.1109/TIM.2018.2876073
146	Suspended 3D AgNPs/CNT nanohybrids for the SERS application	10.1016/j.apsusc.2019.05.179
147	LSPR-Based Cholesterol Biosensor Using Hollow Core Fiber Structure	10.1109/JSEN.2019.2916818
148	Highly Sensitive SPR Biosensor Based on Graphene Oxide and Staphylococcal Protein A Co-Modified TFBG for Human IgG Detection	10.1109/TIM.2018.2875961
149	Fiber optic surface plasmon resonance biosensor for detection of PDGF-BB in serum based on self-assembled aptamer and antifouling peptide monolayer	10.1016/j.bios.2019.111350
150	Use of an electro-optical sensor and phage antibodies for immunodetection of Herbaspirillum	10.1016/j.talanta.2019.04.086
151	Fano resonances based on plasmonic square resonator with high figure of merits and its application in glucose concentrations sensing	10.1007/s11082-019-2007-5
152	Plasmonic Multiplexing Extraordinary Optical Transmission Sensor With Enhanced Sensitivity Through Grooves in Metal Film	10.1109/JSEN.2019.2914962
153	Graphene-based nanocomposites for sensitivity enhancement of surface plasmon resonance sensor for biological and chemical sensing: A review	10.1016/j.bios.2019.111324
154	Preparation of Graphene/ITO Nanorod Metamaterial/U-Bent-Annealing Fiber Sensor and DNA Biomolecule Detection	10.3390/nano9081154
155	Fabrication of a Bare Optical Fiber-Based Biosensor	10.3390/mi10080522
156	MyoRobot 2.0: An advanced biomechatronics platform for automated, environmentally controlled skeletal muscle single fiber biomechanics assessment employing inbuilt real-time optical imaging	10.1016/j.bios.2019.04.052
157	Optical Slot Waveguide With Grating-Loaded Cladding of Silicon and Titanium Dioxide for Label-Free Bio-Sensing	10.1109/JSEN.2019.2910278
158	Lighting Up Biosensors: Now and the Decade To Come	10.1021/acs.analchem.9b00793

№	TITLE_SCIENTIFIC PUBLICATION	DIGITAL OBJECT IDENTIFIER (DOI)
159	Cu/ITO-Coated Uncladded Fiber-Optic Biosensor Based on Surface Plasmon Resonance	10.1109/LPT.2019.2908288
160	Fiber Optic Sensor Based on Vernier Microwave Frequency Comb	10.1109/JLT.2019.2917397
161	Hydrogel optical fibers for continuous glucose monitoring	10.1016/j.bios.2019.05.002
162	Black phosphorus based fiber optic biosensor for ultrasensitive cancer diagnosis	10.1016/j.bios.2019.04.044
163	An Optical Sensor Design Using Surface Modes of Low-Symmetric Photonic Crystals	10.1109/JSEN.2019.2907508
164	Development of an optical biosensor for the detection of Trypanosoma evansi and Plasmodium berghei	. 10.1016/j.saa.2019.04.008
165	Electrical Impedance Characterization of Erythrocyte Response to Cyclic Hypoxia in Sickle Cell Disease	10.1021/acssensors.9b00263
166	Diaphragm-based optical fiber sensor for pulse wave monitoring and cardiovascular diseases diagnosis	10.1002/jbio.201900084
167	Simple and Low-Cost Plasmonic Fiber-Optic Probe as SERS and Biosensing Platform	10.1002/adom.201900337
168	Surface plasmon resonance photonic crystal fiber biosensor based on gold-graphene layers	10.1016/j.yofte.2019.03.018
169	Surface plasmon resonance refractive index sensor based on photonic crystal fiber covering nano-ring gold film	10.1016/j.yofte.2019.03.018
170	Performance Improvement of Refractometric Sensors Through Hybrid Plasmonic-Fano Resonances	10.1109/JLT.2019.2906933
171	Adaptive and sensitive fibre-optic fluorimetric transducer for air- and water-borne	10.1016/j.talanta.2019.02.055
172	An ultra-sensitive aptasensor on optical fibre for the direct detection of bisphenol A	10.1016/j.bios.2019.02.043
173	Iontronic control of GaInAsP photonic crystal nanolaser	10.1063/1.5098119
174	LSPR based optical fiber sensor with chitosan capped gold nanoparticles on BSA for trace detection of Hg (II) in water, soil and food samples	10.1016/j.bios.2019.03.046
175	Improved stability of gold nanoparticles on the optical fiber and their application to refractive index sensor based on localized surface plasmon resonance	10.1016/j.optlastec.2019.02.002
176	Acoustofluidic Micromixing Enabled Hybrid Integrated Colorimetric Sensing, for Rapid Point-of-Care Measurement of Salivary Potassium	10.3390/bios9020073
177	Guided Mode Resonance Sensors with Optimized Figure of Merit	10.3390/nano9060837
178	Modeling and Analysis of SOI Gratings-Based Opto-Fluidic Biosensor for Lab-on-a-Chip Applications	10.3390/photonics6020071
179	A Tunable High-Sensitivity Refractive Index of Analyte Biosensor Based on Metal-Nanoscale Covered Photonic Crystal Fiber With Surface Plasmon Resonance	10.1109/JPHOT.2019.2915235

Nº	TITLE_SCIENTIFIC PUBLICATION	DIGITAL OBJECT IDENTIFIER (DOI)
180	Analysis of Dual-Core Photonic Crystal Fiber Based on Surface Plasmon Resonance Sensor with Segmented Silver Film	10.1007/s11468-018-0846-8
181	Highly sensitive refractive index sensor based on D-shaped PCF with gold-graphene layers on the polished surface	10.1007/s00339-019-2731-5
182	Planar optical waveguide for refractive index determining with high sensitivity and dual-band characteristic for Nano-sensor application	10.1007/s11082-019-1905-x
183	? Gold Nanoparticle-Functionalized Surface Plasmon Resonance Optical Fiber Biosensor: In Situ Detection of Thrombin With 1 n.M Detection Limit	10.1109/JLT.2018.2822827
184	Label-Free Detection of DNA Hybridization Utilizing Dual S-Tapered Thin-Core Fiber Interferometer	10.1109/JLT.2018.2864798
185	In-Situ Detection of Small Biomolecule Interactions Using a Plasmonic Tilted Fiber Grating Sensor	10.1109/JLT.2018.2870337
186	Tricore photonic crystal fibre based refractive index sensor for glucose detection	10.1049/iet-opt.2018.5079
187	A non-spectroscopic optical biosensor for the detection of pathogenic Salmonella Typhimurium based on a stem-loop DNA probe and retro-reflective signaling	10.1186/s40580-019-0186-1
188	Effect of plasmonic materials on photonic crystal fiber based surface plasmon resonance sensors	10.1142/S0217984919501574
189	Detection of Interaction Between Peach-Gum Polysaccharides and Galectin-3 via Surface Plasmon Resonance Imaging	10.3788/LOP56.09.2402
190	Two-dimensional transition metal dichalcogenides assisted biofunctionalized optical fiber SPR biosensor for efficient and rapid detection of bovine serum albumin	10.1038/s41598-019-43531-w
191	Tapered Optical Fibre Sensors: Current Trends and Future Perspectives	10.3390/s19102294
192	Fiber optic plasmonic sensors: Providing sensitive biosensor platforms with minimal lab equipment	10.1016/j.bios.2019.03.020
193	Microscale and Nanoscale Electrophotonic Diagnostic Devices	10.1101/cshperspect.a034249
194	Optical enzymatic biosensor membrane for rapid in situ detection of organohalide in water samples	10.1016/j.microc.2018.12.052
195	U-shaped photonic quasi-crystal fiber sensor with high sensitivity based on surface plasmon resonance	10.7567/1882-0786/ab13bc
196	Low-cost vertical taper for highly efficient light in-coupling in bimodal nanointerferometric waveguide biosensors	10.1088/2515-7647/aafebb/pdf
197	High-Sensitive Fiber Anemometer Based on Surface Plasmon Resonance Effect in Photonic Crystal Fiber	10.1109/JSEN.2019.2895265
198	A novel dual-color total internal reflection fluorescence detecting platform using compact optical structure and silicon-based photodetector	10.1016/j.talanta.2018.12.039
199	Enhanced Sensitivity of Subwavelength Multibox Waveguide Microring Resonator Label-Free Biosensors	10.1109/JSTQE.2018.2821842
200	Carbon-Based, Ultraelastic, Hierarchically Coated Fiber Strain Sensors with Crack-Controllable Beads	10.1021/acsami.9b03204

№	TITLE_SCIENTIFIC PUBLICATION	DIGITAL OBJECT IDENTIFIER (DOI)
201	Waveguide-based chemo- and biosensors: complex emulsions for the detection of caffeine and proteins	10.1039/c9lc00070d
202	Investigation of a low cost tapered plastic fiber optic biosensor based on manipulation of colloidal gold nanoparticles	10.1016/j.optcom.2018.12.088
203	Intensity-modulated nanoplasmonic interferometric sensor for MMP-9 detection	10.1039/C8LC01391H
204	Design approach of solid-core photonic crystal fiber sensor with sensing ring for blood component detection	10.1117/1.JNP.13.026011
205	Construction of efficient bioelectrochemical devices: Improved electricity production from cyanobacteria (Leptolyngbia sp.) based on pi-conjugated conducting polymer/gold nanoparticle composite interfaces	10.1002/bit.26885
206	SPR Label-Free Biosensor with Oxide-Metal-Oxide-Coated D-Typed Optical Fiber: a Theoretical Study	10.1007/s11468-018-0824-1
207	Towards Portable Nanophotonic Sensors	10.3390/s19071715
208	Microfluidics Integrated Lithography-Free Nanophotonic Biosensor for the Detection of Small Molecules	10.1002/adom.201801313
209	A Fiber Optic Biosensor Based on Hydrogel-Immobilized Enzyme Complex for Continuous Determination of Cholesterol and Glucose	10.1007/s12010-018-2897-x
210	Optical Biomarker-based Biosensors for Cancer/Infectious Disease Medical Diagnoses	10.1097/PAI.0000000000000586
211	Ultraflexible Nanowire Array for Label- and Distortion-Free Cellular Force Tracking	10.1021/acs.nanolett.8b02568
212	A theoretical study of a plasmonic sensor comprising a gold nano-disk array on gold film with a SiO2 spacer	10.1088/1674-1056/28/4/044201
213	An effective triple-band enhanced-infrared-absorption detection by honeycomb-shaped metamaterial-plasmonic absorber	10.1016/j.sna.2019.02.006
214	A Compact and Broadband Photonic Crystal Fiber Polarization Filter Based on a Plasmonic Resonant Thin Gold Film	10.1109/JPHOT.2019.2899117
215	Highly Flexible and Voltage Based Wavelength Tunable Biosensor	10.1002/pssa.201800633
216	Plasmonic biosensors for bacterial endotoxin detection on biomimetic C-18 supported fiber optic probes	10.1016/j.bios.2018.12.045
217	Real-time monitoring of skin ethanol gas by a high-sensitivity gas phase biosensor (bio-sniffer) for the non-invasive evaluation of volatile compounds	10.1016/j.bios.2018.09.070
218	Separation and Detection of Escherichia coli and Saccharomyces cerevisiae Using a Microfluidic Device Integrated with an Optical Fibre	10.3390/bios9010040
219	Waveguiding and SERS Simplified Raman Spectroscopy on Biological Samples	10.3390/bios9010037
220	Effects of dispersion, absorption and interface fluctuation on the reflection spectra of porous silicon microcavity devices	10.1007/s11801-019-8148-8
221	Numerical modeling of graphene-coated fiber optic surface plasmon resonance biosensor for BRCA1 and BRCA2 genetic breast cancer detection	10.1117/1.OE.58.3.037104
222	Modeling of a fiber-optic surface plasmon resonance biosensor employing phosphorene for sensing applications	10.1117/1.OE.58.3.037103
223	Shape monitoring of morphing wing using micro optical sensors with different embedded depth	10.1016/j.yofte.2018.12.025
224	A dual-channel surface plasmon resonance biosensor based on a photonic crystal fiber for multianalyte sensing	10.1007/s10825-019-01305-7

№	TITLE_SCIENTIFIC PUBLICATION	DIGITAL OBJECT IDENTIFIER (DOI)
225	Label-free biosensing using a microring resonator integrated with poly-(dimethylsiloxane) microfluidic channels	10.1063/1.5074134
226	Polymer-Templated Gold Nanoparticles on Optical Fibers for Enhanced-Sensitivity Localized Surface Plasmon Resonance Biosensors	10.1021/acssensors.8b01372
227	Core-clad phosphate glass fibers for biosensing	10.1016/j.msec.2018.11.038
228	High-Q Slot-Mode Photonic Crystal Nanobeam Cavity Biosensor With Optomechanically Enhanced Sensitivity	10.1109/JSTQE.2018.2880592
229	Design and Analysis of Surface-Plasmon-Resonance-Based Photonic Quasi-Crystal Fiber Biosensor for High-Refractive-Index Liquid Analytes	10.1109/JSTQE.2018.2873481
230	Optimized Bent Part Coupling SiON Racetrack Resonators for Biological Sensing	10.1109/JSEN.2018.2879784
231	Functionalized gold electroless-plated optical fiber gratings for reliable surface biosensing	10.1016/j.snb.2018.09.115
232	Photonic integrated circuits for Department of Defense-relevant chemical and biological sensing applications: state-of-the-art and future outlooks	10.1117/1.OE.58.2.020901
233	Analysis of highly sensitive biosensor for glucose based on a one-dimensional photonic crystal nanocavity	10.1117/1.OE.58.2.027102
234	Disk-based one-dimensional photonic crystal slabs for label-free immunosensing	10.1016/j.bios.2018.11.005
235	The terahertz electromagnetically induced transparency-like metamaterials for sensitive biosensors in the detection of cancer cells	10.1016/j.bios.2018.11.014
236	Rapid detection of Escherichia coli using fiber optic surface plasmon resonance immunosensor based on biofunctionalized Molybdenum disulfide (MoS2) nanosheets	10.1016/j.bios.2018.11.006
237	Microfluidic chip coupled with optical biosensors for simultaneous detection of multiple analytes: A review	10.1016/j.bios.2018.11.032
238	Development of dual-color total internal reflection fluorescence biosensor for simultaneous quantitation of two small molecules and their affinity constants with antibodies	10.1016/j.bios.2018.12.010
239	Hybrid Metasurface-Based Mid-Infrared Biosensor for Simultaneous Quantification and Identification of Monolayer Protein	10.1021/acsphotonics.8b01470
240	Highly sensitive SPR PCF biosensors based on Ag/TiN and Ag/ZrN configurations	10.1007/s11082-019-1764-5
241	Propagation Controlled Photonic Crystal Fiber-Based Plasmonic Sensor via Scaled-Down Approach	10.1109/JSEN.2018.2880161
242	A sensitive biosensor based on optical bistability in a semiconductor quantum dot-DNA nanohybrid	10.1088/1361-6463/aaec48
243	Optical investigations of electrochemical processes using a long-period fiber grating functionalized by indium tin oxide	10.1016/j.snb.2018.10.001
244	Improvement of Resolution of Liquid Refractive Index Measurement Using Metallic Grating	10.2528/PIERM19060304
245	Cascaded-Microrings Biosensors Fabricated on a Polymer Platform	10.3390/s19010181
246	Development of Electrochemical Methods to Enzymatically Detect Lactate and Glucose Using Imaginary Impedance for Enhanced Management of Glycemic Compromised Patients	10.1615/CritRevBiomedEng.2019026533
247	Probing Lead Ion Contamination in Aqueous Solution Through Bio-Inspired Surface Modification of Gold Nanoparticles on D-Shaped Fiber	10.1109/TNANO.2019.2929058
248	A Novel Fiber-Based Symmetrical Long-Range Surface Plasmon Resonance Biosensor With High Quality Factor and Temperature Self-Reference	10.1109/TNANO.2019.2947697

№	TITLE_SCIENTIFIC PUBLICATION	DIGITAL OBJECT IDENTIFIER (DOI)
249	A simulation analysis for dimensioning of an amorphous silicon planar waveguide structure suitable to be used as a surface plasmon resonance biosensor	10.1117/12.2526414
250	200-mm Silicon Photonics Technology Development	10.1117/12.2537254
251	Extrinsic Plasmonic Optical Fiber Sensors based on POFs and Bacterial Cellulose slab waveguides	10.1117/12.2539335
252	Graphene Oxide-functionalized Long Period Grating for biosensing applications	10.1016/j.bios.2017.03.004
253	2D stiffness mapping for localizing osteoarthritic degenerated cartilage by using a fast indentation system based on fiber Bragg gratings	10.1117/12.2539252
254	Fiber-optics: a new route towards ultra-low detection limit label-free biosensing	10.1117/12.2540008
255	Advances in celiac disease testing	10.1016/bs.acc.2019.03.001
256	Plasmonic nanolasers based on graphene-insulator-metal platform	10.23919/MOC46630.2019.8982904
257	Janus particles: recent advances in the biomedical applications	10.2147/IJN.S169030
258	Microring resonator biosensing platform for sensitive detection of thrombin	10.1117/12.2508782
259	Breaking the trade-off between Q-factor and sensitivity for high-Q slot mode photonic crystal nanobeam cavity biosensors with optomechanical feedback	10.1117/12.2509659
260	Do you need a tunable laser for resonant cavity optical sensors?	10.1117/12.2522352
261	Laser biosensor based on micromechanical oscillator	10.1117/12.2519607
262	Influence of saline background on microstructured optical fibers optical properties	10.1117/12.2523335
263	Fully Integrated Liquid-Core Waveguide Fluorescence Lifetime Detection Microsystem for DNA Biosensing	10.1109/ACCESS.2019.2934764
264	Thin Film Coating of Copper Nanoparticles with DC Magnetron Sputtering via Physical Vapor Deposition	10.1063/1.5118128
265	Effect of anisotropy on the spectral characteristics of one-dimensional porous silicon photonic crystal microcavity for optical sensing applications	10.1117/1.JNP.13.016012
266	Wavelength dispersion phenomena observed for emitted optical radiation from a p plus nn plus silicon avalanche mode light-emitting device in a radio frequency bipolar-integrated circuitry	10.1117/1.OE.58.1.017104
267	A Hi-Bi Ultra-Sensitive Surface Plasmon Resonance Fiber Sensor	10.1109/ACCESS.2019.2922663
268	Nano-plate biosensor array using ultrafast heat transport through proteins	10.1016/j.snb.2018.09.030
269	Fiber-enhanced Raman spectroscopy as a tool for an early detection of Alzheimer's disease biomarkers	10.1117/12.2516545
270	. Mach-Zehnder interferometer based on core-offset splicing technique for sensing applications	10.1117/12.2516545
271	Real-time detection of BSA concentration based on a Mach-Zehnder interferometric biosensor	10.1117/12.2522635
272	Label-Free DNA Hybridization Monitoring through mFBG Biosensor with Self-Assembly Technique	10.1117/12.2521352
273	Integrated photonics for NASA applications	10.1117/12.2509808
274	Multi-functional plasmonic biosensor based on alcohol filled PCF	10.1117/12.2509417

№	TITLE_SCIENTIFIC PUBLICATION	DIGITAL OBJECT IDENTIFIER (DOI)
275	Bimetallic Surface Plasmon Resonance Photonic Crystal Fiber Biosensor Using Refractory Plasmonic Material	10.1117/12.2509436
276	Porous silicon sensors: From on-chip to mobile diagnostics	10.1117/12.2508685
277	Design and optimization of compact silicon photonic sensors	10.1117/12.2502509
278	Realizing Micro and Nano Optical Bio Sensors on Chip	10.1117/12.2502491
279	Continuous wave laser induced nonlinear optical response of nitrogen doped graphene oxide	10.1117/12.2502491
280	Nucleic acid functionalized fiber optic probes for sensing in evanescent wave: optimization and application	10.1039/c8ra10125f
281	Integrated multichannel all-fiber optofluidic biosensing platform for sensitive and simultaneous detection of trace analytes	10.1016/j.aca.2018.07.067
282	Development of novel portable and reusable fiber optical chemiluminescent biosensor and its application for sensitive detection of microcystin-LR	10.1016/j.bios.2018.08.062
283	Localized surface plasmon resonance based U-shaped optical fiber probe for the detection of Pb2+ in aqueous medium	10.1016/j.snb.2018.08.086
284	Polymeric biomaterials for biophotonic applications	10.1016/j.snb.2018.08.065
285	MXene-Based Nonlinear Optical Information Converter for All-Optical Modulator and Switcher	10.1002/lpor.201800215
286	Monolithically integrated InGaAsP multiple quantum well tunable laser diode for integrated optic surface plasmon resonance sensing	10.1117/1.OE.57.12.120503
287	Surface plasmon resonance biosensor based on graphene oxide/silver coated polymer cladding silica fiber	10.1016/j.snb.2018.08.065
288	Three-core photonic crystal fiber surface plasmon resonance sensor	10.1016/j.yofte.2018.11.014
289	High sensitivity photonic crystal fiber-based refractive index microbiosensor	10.1016/j.yofte.2018.09.016
290	High Sensitivity Photonic Crystal Fiber Refractive Index Sensor with Gold Coated Externally Based on Surface Plasmon Resonance	10.3390/mi9120640
291	Etched Fiber Bragg Grating Biosensor Functionalized with Aptamers for Detection of Thrombin	10.3390/s18124298
292	Fano Resonance in Waveguide Coupled Surface Exciton Polaritons: Theory and Application in Biosensor	10.3390/s18124437
293	Enhanced sensitivity of hemoglobin sensor using dual-core photonic crystal fiber	10.1007/s11082-018-1710-y
294	Multichannel Long-Range Surface Plasmon Waveguides for Parallel Biosensing	10.1109/JLT.2018.2875953
295	Design and numerical analysis of a novel dual-polarized refractive index sensor based on D-shaped photonic crystal fiber	10.1088/1681-7575/aae757
296	Ultrasensitive biosensor based on long period grating coated with polycarbonate-graphene oxide multilayer	10.1016/j.snb.2018.08.002
297	Rapid and real-time diagnosis of hypoalbuminemia using an extraordinary optical transmission biosensor	10.1016/j.snb.2018.07.119
298	⍰ Photochemical deposition fabricated highly sensitive localized surface plasmon resonance based optical fiber sensor	10.1016/j.optcom.2018.06.075

№	TITLE_SCIENTIFIC PUBLICATION	DIGITAL OBJECT IDENTIFIER (DOI)
299	Silicon plasmonic integrated interferometer sensor for lab on chip applications	10.1016/j.optcom.2018.02.053
300	Highly sensitive detection of exosomes by 3D plasmonic photonic crystal biosensor	10.1039/C8NR07051B
301	? Real-time detection of prostate-specific antigens using a highly reliable fiber-optic localized surface plasmon resonance sensor combined with micro fluidic channel	10.1016/j.snb.2018.07.007
302	Nano-electric field sensor based on Two Dimensional Photonic Crystal resonator	10.1016/j.optmat.2018.09.016
303	In-situ ultrasensitive label-free DNA hybridization detection using optical fiber specklegram	10.1016/j.snb.2018.05.099
304	Sensitivity enhanced SPR immunosensor based on graphene oxide and SPA co-modified photonic crystal fiber	10.1016/j.optlastec.2018.05.006
305	A Fluidic Biosensor Based on a Phase-Sensitive Low-Coherence Spectral-Domain Interferometer	10.3390/s18113757
306	Selective Uropathogenic E-coli Detection Using Crossed Surface-Relief Gratings	10.3390/s18113634
307	Robust Optimization of Nanoslit Array Sensor Based on Extraordinary Optical Transmission	10.1109/JSEN.2018.2870300
308	Surface Plasmon Resonance Sensor Based on Modified D-Shaped Photonic Crystal Fiber for Wider Range of Refractive Index Detection	10.1109/JSEN.2018.2865514
309	Directed self-assembly of a high-chi block copolymer for the fabrication of optical nanoresonators	10.1039/C8NR05831H
310	Multilayered Nanoplasmonic Arrays for Self-Referenced Biosensing	10.1021/acsami.8b12604
311	Theoretical evaluation of a fiber-optic SPR biosensor based on a gold layer treated with thiol acid	10.1051/epjap/2018180059
312	Plasmonic Biosensor in NIR with Chalcogenide Glass Material: On the Role of Probe Geometry, Wavelength, and 2D Material	10.1007/s11220-018-0220-0
313	Rapid detection of cocaine using aptamer-based biosensor on an evanescent wave fibre platform	10.1098/rsos.180821
314	Photonic crystal enhanced fluorescence immunoassay on diatom biosilica	10.1002/jbio.201800009
315	Optical Micro/Nanofiber-Based Localized Surface Plasmon Resonance Biosensors: Fiber Diameter Dependence	10.3390/s18103295
316	Silicon Photonic Biosensors Using Label-Free Detection	10.3390/s18103519
317	Research on dual-core photonic crystal fiber based on local surface plasmon resonance sensor with silver nanowires	10.1117/1.JNP.12.046015
318	A Plasmonic Fiber Based Glucometer and Its Temperature Dependence	10.3390/mi9100506
319	Design of a surface plasmon resonance biosensor based on photonic crystal fiber with elliptical holes	10.1007/s10043-018-0447-y
320	Highly sensitive nano-scale plasmonic biosensor utilizing Fano resonance metasurface in THz range: Numerical study	10.1016/j.physe.2018.07.039
321	Optical conductivity-based ultrasensitive mid-infrared biosensing on a hybrid metasurface	10.1038/s41377-018-0066-1
322	Aluminum plasmonic waveguides co-integrated with Si3N4 photonics using CMOS processes	10.1038/s41598-018-31736-4
323	Optical Nitrite Biosensor based on Hemoglobin-Immobilized Polyacrylate Microspheres	10.17576/jsm-2018-4709-10

№	TITLE_SCIENTIFIC PUBLICATION	DIGITAL OBJECT IDENTIFIER (DOI)
324	Thin-core fiber-optic biosensor for DNA hybridization detection	10.1007/s11801-018-8054-5
325	Improved Magneto-Optic Surface Plasmon Resonance Biosensors	10.3390/photonics5030015
326	Femtosecond-Pulsed Laser Written and Etched Fiber Bragg Gratings for Fiber-Optical Biosensing	10.3390/s18092844
327	Selective and sensitive Escherichia coli detection based on a T4 bacteriophage-immobilized multimode microfiber	10.1002/jbio.201800012
328	Highly sensitive PCF-SPR biosensor for hyperthermia temperature monitoring	10.1007/s12596-018-0468-8
329	Plastic optical fiber immunosensor for fast detection of sulfate-reducing bacteria	10.1016/j.measurement.2018.04.088
330	Label-free ferrule-top optical fiber micro-cantilever biosensor	10.1016/j.sna.2018.07.014
331	LED-Based Portable Optical Biosensor for Measurement of Serum Urea Levels Using Urease Immobilized Agarose-Guar Gum Composite Film	10.1109/JSEN.2018.2850909
332	A wavelength-modulated localized surface plasmon resonance (LSPR) optical fiber sensor for sensitive detection of mercury(II) ion by gold nanoparticles-DNA conjugates	10.1016/j.bios.2018.05.004
333	Dual-core all-fiber integrated immunosensor for detection of protein antigens	10.1016/j.bios.2018.05.008
334	Autler-Townes splitting biosensing based on a nonuniform photonic crystal waveguide with feedback loop	10.1364/AO.57.006976
335	Buried Rib SiO2 Multimode Interference Waveguides for Optofluidic Multiplexing	10.1109/LPT.2018.2858258
336	A Highly Sensitive Metal-Insulator-Metal Ring Resonator-Based Nanophotonic Structure for Biosensing Applications	10.1109/JSEN.2018.2849825
337	Amplification-free, sequence-specific 16S rRNA detection at 1 aM	10.1039/C8LC00452H
338	Optical bio-chemical sensors based on whispering gallery mode resonators	10.1039/C8NR03709D
339	3D-Printed Biosensor Arrays for Medical Diagnostics	10.3390/mi9080394
340	Photonic crystal microcavity as a highly sensitive platform for RI detection	10.1016/j.cjph.2018.05.010
341	A Highly Sensitive SPR Sensors Based on Two Parallel PCFs for Low Refractive Index Detection	10.1109/JPHOT.2018.2856273
342	A High-Performance Plasmonic Nanosensor Based on an Elliptical Nanorod in an MIM Configuration	10.1109/JSEN.2018.2847760
343	Gold-nanoparticle-based Fiber Optic Sensor for Sensing the Refractive Index of Environmental Solutions	—
344	Resonant Wavelength Shift Detection System Based on a Gradient Grating Period Guided-Mode Resonance	10.1109/JPHOT.2018.2857505
345	Optimization of porous silicon waveguide design for micro-ring resonator sensing applications	10.1088/2040-8986/aad01b
346	Twin Core Photonic Crystal Fiber Plasmonic Refractive Index Sensor	10.1109/JSEN.2018.2841035
347	Analysis and Improvement of a Dual-Core Photonic Crystal Fiber Sensor	10.3390/s18072051
348	Hand-held optical sensor using denatured antibody coated electro-active polymer for ultra-trace detection of copper in blood serum and environmental samples	10.1016/j.bios.2018.03.040
349	Photonic crystal resonances for sensing and imaging	10.1088/2040-8986/aac75b
350	A Review of advancements (2007-2017) in plasmonics-based optical fiber sensors	10.1016/j.yofte.2018.03.008

№	TITLE_SCIENTIFIC PUBLICATION	DIGITAL OBJECT IDENTIFIER (DOI)
351	Smallest microhouse in the world, assembled on the facet of an optical fiber by origami and welded in the mu Robotex nanofactory	10.1116/1.5020128
352	Theoretical Design of an Integrated Optical Sensor for a Standard Immunoassay	10.1109/JSEN.2018.2824852
353	Colorimetric determination of Cu2+ ions in water and milk by apo-tyrosinase disc	10.1016/j.snb.2018.02.160
354	An optofluidic metasurface for lateral flow-through detection of breast cancer biomarker	10.1016/j.bios.2018.02.038
355	Design of turn around point long period fiber grating sensor with Au-nanoparticle self monolayer	10.1016/j.optlastec.2017.12.025
356	Tunable Surface Plasmon Resonance Sensor Based on Photonic Crystal Fiber Filled with Gold Nanoshells	10.1007/s11468-017-0570-9
357	Ultrasensitive label-free optical microfiber coupler biosensor for detection of cardiac troponin I based on interference turning point effect	10.1016/j.bios.2018.01.061
358	A Smartphone-Based Red-Green Dual Color Fiber Optic Surface Plasmon Resonance Sensor	10.1109/LPT.2017.2788560
359	Graphene oxide functionalized long period fiber grating for highly sensitive hemoglobin detection	10.1016/j.snb.2018.01.117
360	Highly sensitive colorimetric paper sensor for methyl isothiocyanate (MITC): Using its toxicological reaction	10.1016/j.snb.2018.01.086
361	Silicon Plasmonics On-Chip Mid-IR Gas Sensor	10.1109/LPT.2018.2799208
362	Fabrication of hexagonal star-shaped and ring-shaped patterns arrays by Mie resonance sphere-lens-lithography	10.1016/j.apsusc.2017.12.200
363	Optical fiber tips for biological applications: From light confinement, biosensing to bioparticles manipulation	10.1016/j.bbagen.2018.02.008
364	Microstructure and magneto-optical surface plasmon resonance of Co/Au multilayers	10.1088/2399-6528/aac0e0
365	A simple approach for fabrication of optical affinity-based bioanalytical microsystem on polymeric PEN foils	10.1016/j.colsurfb.2018.01.048
366	Facile Fabrication of Novel Sensing System for Size Detection of Nanoparticles	10.1109/TNANO.2018.2806306
367	Robust immunosensing system based on biotin-streptavidin coupling for spatially localized femtogram mL(-1) level detection of interleukin-6	10.1016/j.bios.2017.11.023
368	An optical fiber-based LSPR aptasensor for simple and rapid in-situ detection of ochratoxin A	10.1016/j.bios.2017.11.062
369	Alternative SNP detection platforms, HRM and biosensors, for varietal identification in Vitis vinifera L. using F3H and LDOX genes	10.1038/s41598-018-24158-9
370	Hydrogen peroxide and glucose concentration measurement using optical fiber grating sensors with corrodible plasmonic nanocoatings	10.1364/BOE.9.001735
371	Graphene/Au-Enhanced Plastic Clad Silica Fiber Optic Surface Plasmon Resonance Sensor	10.1007/s11468-017-0534-0
372	On-Chip Oval-Shaped Nanocavity Photonic Crystal Waveguide Biosensor for Detection of Foodborne Pathogens	10.1007/s11468-017-0529-x
373	Glucose Sensor Using U-Shaped Optical Fiber Probe with Gold Nanoparticles and Glucose Oxidase	10.3390/s18041217
374	High-Sensitivity Refractive Index Sensing Based on Fano Resonances in a Photonic Crystal Cavity-Coupled Microring Resonator	10.1109/JPHOT.2018.2815622
375	Mid-Infrared Sensor Based on a Suspended Microracetrack Resonator With Lateral Subwavelength-Grating Metamaterial Cladding	10.1109/JPHOT.2018.2809662.
376	Urinary p-cresol diagnosis using nanocomposite of ZnO/MoS2 and molecular imprinted polymer on optical fiber based lossy mode resonance sensor	10.1016/j.bios.2017.10.029

№	TITLE_SCIENTIFIC PUBLICATION	DIGITAL OBJECT IDENTIFIER (DOI)
377	Giant enhancement of emission efficiency and light directivity by using hyperbolic metacavity on deep-ultraviolet AlGaN emitter	10.1016/j.nanoen.2018.01.020
378	Highly sensitive photonic crystal fiber biosensor based on titanium nitride	10.1007/s11082-018-1397-0
379	A high-sensitivity photonic crystal fiber (PCF) based on the surface plasmon resonance (SPR) biosensor for detection of density alteration in non-physiological cells (DANCE)	10.1016/j.opelre.2018.01.001
380	Miniaturized Sample Preparation and Rapid Detection of Arsenite in Contaminated Soil Using a Smartphone	10.3390/s18030777
381	Integrated Optical Mach-Zehnder Interferometer Based on Organic-Inorganic Hybrids for Photonics-on-a-Chip Biosensing Applications	10.3390/s18030840
382	Geometrical comparison of photonic crystal fiber-based surface plasmon resonance sensors	10.1117/1.OE.57.3.030801
383	Photoinducible silane diazirine as an effective crosslinker in the construction of a chemiluminescent immunosensor targeting a model E. coli analyte	10.1016/j.snb.2017.10.058
384	Uric acid sensing using tapered silica optical fiber coated with zinc oxide nanorods	10.1002/mop.31032
385	Surface plasmon resonance fiber optic biosensor-based graphene and photonic crystal	10.1142/S0217984918500720
386	Phase-sensitive plasmonic biosensor using a portable and large field-of-view interferometric microarray imager	10.1038/lsa.2017.152
387	A label-free cardiac biomarker immunosensor based on phase-shifted microfiber Bragg grating	10.1016/j.bios.2017.08.061
388	A novel immunosensor based on excessively tilted fiber grating coated with gold nanospheres improves the detection limit of Newcastle disease virus	10.1016/j.bios.2017.08.064
389	Functionalized Long Period Grating-Plasmonic Fiber Sensor Applied to the Detection of Glyphosate in Water	10.1109/JLT.2017.2739243
390	Plasmonic Refractive Index Sensor Employing Niobium Nanofilm on Photonic Crystal Fiber	10.1109/LPT.2017.2786475
391	Colorimetric and Fiber Optic Sensing of Cysteine Using Green Synthesized Gold Nanoparticles	10.1007/s11468-017-0517-1
392	Surface Plasmon Resonance and Bending Loss-Based U-Shaped Plastic Optical Fiber Biosensors	10.3390/s18020648
393	Carbohydrate-protein interactions characterized by dual polarization hybrid plasmonic waveguide	10.1515/ntrev-2017-0165
394	Fibre Optic SPR Sensor Using Functionalized CNTs for the Detection of SMX: Comparison with Enzymatic Approach	10.1007/s11468-017-0499-z
395	. Spectral Optical Readout of Rectangular-Miniature Hollow Glass Tubing for Refractive Index Sensing	10.3390/s18020603
396	A whole cell bio-optode based on immobilized nitrite-degrading microorganism on the acrylic microspheres for visual quantitation of nitrite ion	10.1016/j.snb.2017.09.102
397	Immobilized optical fiber microprobe for selective and high sensitive glucose detection	10.1016/j.snb.2017.09.123
398	Label free detection for DNA hybridization using surface plasmon photonic crystal fiber biosensor	10.1007/s11082-017-1302-2
399	Optical fiber sensor for dual sensing of H2O2 and DO based on CdSe/ZnS QDs and Ru(dpp)3(2+) embedded in EC matrix	10.1016/j.snb.2017.08.071
400	LSPR- and SPR-Based Fiber-Optic Cholesterol Sensor Using Immobilization of Cholesterol Oxidase Over Silver Nanoparticles Coated Graphene Oxide Nanosheets	10.1109/JSEN.2017.2779519
401	Highly Sensitive Terahertz Gas Sensor Based on Surface Plasmon Resonance With Graphene	10.1109/JPHOT.2017.2778245
402	DNA origami nanorobot fiber optic genosensor to TMV	10.1016/j.bios.2017.07.051

№	TITLE_SCIENTIFIC PUBLICATION	DIGITAL OBJECT IDENTIFIER (DOI)
403	Ultrahigh Sensitivity Plasmonic Refractive-Index Sensor for Aqueous Environment	10.1109/LPT.2017.2779142
404	Nonlinear Properties Of Water-soluble Ag2S And PbS Quantum Dots Under Picosecond Laser Pulses	10.1088/1755-1315/186/4/012076
405	Plasmonic Interferometer Array Biochip as a New Mobile Medical Device for Cancer Detection	10.1109/JSTQE.2018.2865418
406	Enhancement of plasma resonance in a Hi-Bi D-shaped photonic crystal fiber SPR sensor	10.1117/12.2511830
407	Wavelength-Division-Multiplexed Fiber Bragg Grating sensor integrated with Surface Plasmon Resonance with the capability of multipoint, real-time remote sensing	10.1143/JJAP.45.4588
408	Resonant coupling from photonic crystal surfaces to plasmonic nanoantennas: Principles, detection instruments, and applications in digital resolution biosensing	10.1117/12.2285828
409	Lab-on-a-chip photonic biosensor for detection of antigens	10.1117/12.2321128
410	Ultracompact High-sensitivity Biosensing Based on Regenerable Laminated Bio-conjugation in Integrated Photonic Circuits	10.1117/12.2321211
411	Detection of various Thrombin concentrations using etched fiber Bragg gratings functionalized with DNA aptamer	10.1117/12.2502457
412	Fabrication and Performance of Ge-on-Si PIN Photodetectors	10.1117/12.2501106
413	High-resolution magnetic field biosensor based on optical resonators	10.1117/12.2307587
414	Highly Sensitive SPR based PCF for Biological Substance Sensing: Design and Analysis	10.1117/12.2307061
415	Integrated sensor biopsy device for real time tissue metabolism analysis	10.1117/12.2290323
416	Bragg grating in micro-scaled optical fiber for cardiac biomarker sensing	10.1117/12.2284649
417	Analysis of Highly Sensitive Surface Plasmon Photonic Crystal Fiber Biosensor	10.1117/12.2290998
418	Novel Ultra Low Power Optical Memory using Liquid Crystal	10.1117/12.2306461
419	Cartilage microindentation using cylindrical and spherical optical fiber indenters with integrated Bragg gratings as force sensors	10.1117/12.2289493
420	Fundamentals and biomedical applications of photonic crystals: An overview	10.1117/12.2282019
421	Design optimization of a single-mode microring resonator for label-free detection of biomarkers within a tunable spectral range of 2 nm	10.1117/12.2318934
422	Highly Sensitive Photonic Crystal Fiber Biosensor Based on Alternative Plasmonic Material	10.1117/12.2306300
423	Development towards Compact Nitrocellulose Interferometric Biochips for Dry Eye diagnosis based on MMP9, S100A6 and CST4 biomarkers using a Point-of-Care device	10.1117/12.2288257
424	D-Shaped photonic crystal fiber biosensor based on silver-graphene	10.1016/j.ijleo.2018.04.119
425	Resonant wavelength shift detection system based on a gradient grating period guided-mode resonance filter	10.1117/12.2320414
426	Sensitivity enhancement of surface plasmon resonance biosensor with graphene sandwiched between two metal films	10.1117/12.2320025
427	Intermixed ingaasp MQW Tunable laser diode suitable for probing surface plasmon resonance optical sensor	10.1117/12.2503553
428	Fish-bone subwavelength grating waveguide photonic integrated circuit sensor array	10.1117/12.2305506
429	Application of Fiber Optic Biosensor in Detection of Sports Analeptic	10.7546/ijba.2018.22.4.301-314

№	TITLE_SCIENTIFIC PUBLICATION	DIGITAL OBJECT IDENTIFIER (DOI)
430	Computational Study for Optimization of a Plasmon FET as a Molecular Biosensor	10.1117/12.2288041
431	Ultrasensitive colocalization detection based on plasmonic nanolithography with molecular-overlapped optical near-fields	10.1117/12.2291449
432	Silicon photonic resonator for label-free bio-sensing application	10.1117/12.2300961
433	Silicon-photonic interferometric biosensor using active phase demodulation	10.1117/12.2287803
434	Investigation of ultrahigh sensitivity in GaInAsP nanolaser biosensor	10.1117/12.2289408
435	A highly sensitive Bezier polygonal hollow core photonic crystal fiber biosensor based on surface plasmon resonance	10.1016/j.ijleo.2018.06.039
436	Innovative 2D-nanomaterial integrated fiber optic sensors for biochemical applications	10.1117/12.2307115
437	Photonic crystal fiber-based plasmonic biosensor with external sensing approach	10.1117/1.JNP.12.012503
438	A highly sensitive surface plasmon resonance biosensor using photonic crystal fiber filled with gold nanowire encircled by silicon lining	10.1016/j.ijleo.2017.10.157
439	Square array photonic crystal fiber-based surface plasmon resonance refractive index sensor	10.1142/S0217984917503523
440	Surface plasmon resonance sensor based on photonic crystal fiber filled with gold-silica-gold multilayer nanoshells	10.1016/j.optcom.2017.08.042
441	Zinc(II) phthalocyanine fused in peripheral positions octa-substituted with alkyl linked carbazole: Synthesis, electropolymerization and its electro-optic and biosensor applications	10.1016/j.bios.2017.06.028
442	A label-free fiber optic SPR biosensor for specific detection of C-reactive protein	10.1038/s41598-017-17276-3
443	A Low-Cost and Portable Dual-Channel Fiber Optic Surface Plasmon Resonance System.	10.3390/s17122797
444	Sensitivity Analysis of Different Shapes of a Plastic Optical Fiber-Based Immunosensor for Escherichia coli: Simulation and Experimental Results	10.3390/s17122944
445	Two-Channel SPR Sensor Combined Application of Polymer- and Vitreous-Clad Optic Fibers	10.3390/s17122862
446	A Highly Sensitive Dual-Core Photonic Crystal Fiber Based on a Surface Plasmon Resonance Biosensor with Silver-Graphene Layer	10.1007/s11468-016-0453-5
447	Design, fabrication and characterisation of silica-titania thin film coated over coupled long period fibre gratings: Towards bio-sensing applications	10.1016/j.snb.2017.06.139
448	Split-ring resonator-based sensors on flexible substrates for glaucoma monitoring	10.1016/j.sna.2017.10.054
449	Graphene plasmonic nanogratings for biomolecular sensing in liquid	10.1007/s00339-017-1380-9
450	Fiber-Based Helical Channels Refractive Index Sensor Available for Microfluidic Chip	10.1109/LPT.2017.2765314
451	Indium Tin Oxide Coated Two-Mode Fiber for Enhanced SPR Sensor in Near-Infrared Region	10.1109/JPHOT.2017.2757513
452	Compact plasmonic optical biosensors based on nanostructured gradient index lenses integrated into microfluidic cells	10.1039/C7NR04097K

№	TITLE_SCIENTIFIC PUBLICATION	DIGITAL OBJECT IDENTIFIER (DOI)
453	Graphene Effect on the Improvement of the Response of Optical Fiber SPR Sensor	10.1109/JSEN.2017.2758258
454	Coagulation measurement from whole blood using vibrating optical fiber in a disposable cartridge	10.1117/1.JBO.22.11.117001
455	Square-microfiber-integrated biosensor for label-free DNA hybridization detection	10.1016/j.snb.2017.07.168
456	U-bent fiber optic SPR sensor based on graphene/AgNPs	10.1016/j.snb.2017.05.045
457	Development of a chemiluminescent DNA fibre optic genosensor to Hepatitis A Virus (HAV)	10.1016/j.talanta.2017.06.036
458	Multimode smartphone biosensing: the transmission, reflection, and intensity spectral (TRI)-analyzer	10.1039/c7lc00633k
459	Localized surface plasmon resonance-based fiber-optic sensor for the detection of triacylglycerides using silver nanoparticles	10.1117/1.JBO.22.10.107001
460	A Novel Fiber Optic Surface Plasmon Resonance Biosensors with Special Boronic Acid Derivative to Detect Glycoprotein	10.3390/s17102259
461	Modulation of Visible and Near-Infrared Surface Plasmon Resonance of Au Nanoparticles Based on Highly Doped Graphene	10.1007/s11468-016-0389-9
462	Picomole Dopamine Detection Using Optical Chips	10.1007/s11468-016-0412-1
463	Surface plasmon excitation using a Fourier-transform infrared spectrometer: Live cell and bacteria sensing	10.1063/1.4997388
464	Specific Detection of Antibiotics by Silicon-on-Chip Photonic Crystal Biosensor Arrays	10.1109/JSEN.2017.2734885
465	Repetitive Immunosensor with a Fiber-Optic Device and Antibody-Coated Magnetic Beads for Semi-Continuous Monitoring of Escherichia coli O157:H7	10.3390/s17092145
466	Manufacturing and Spectral Features of Different Types of Long Period Fiber Gratings: Phase-Shifted, Turn-Around Point, Internally Tilted, and Pseudo-Random	10.3390/fib5030029
467	A Study on Refractive Index Sensors Based on Optical Micro-Ring Resonators	10.1007/s13320-017-0418-0
468	Fully Integrated Fluorescence Biosensors On-Chip Employing Multi-Functional Nanoplasmonic Optical Structures in CMOS	10.1109/JSSC.2017.2712612
469	Demonstration of low-cost and compact SPR optical transducer through edge light coupling	10.1049/mnl.2017.0048
470	Acetone Biosensor Based on Fluorometry of Reduced Nicotinamide Adenine Dinucleotide Consumption in Reversible Reaction by Secondary Alcohol Dehydrogenase	10.1109/JSEN.2017.2721964
471	An automated optofluidic biosensor platform combining interferometric sensors and injection moulded microfluidics	10.1039/C7LC00524E
472	Immunodetection of salivary biomarkers by an optical microfluidic biosensor with polyethylenimine-modified polythiophene-C-70 organic photodetectors	10.1016/j.bios.2017.03.005
473	Label-Free Detection of DNA Hybridization Using a Reflective Microfiber Bragg Grating Biosensor With Self-Assembly Technique	10.1109/JLT.2017.2659778
474	Specific Detection of Aquaporin-2 Using Plasmonic Tilted Fiber Grating Sensors	10.1109/JLT.2016.2645233
475	Optical Fiber Sensors for Label-Free DNA Detection	10.1109/JLT.2016.2607024
476	Graphene-MoS2 Hybrid Structure Enhanced Fiber Optic Surface Plasmon Resonance Sensor	10.1007/s11468-016-0377-0
477	Ultracompact On-Chip Multiplexed Sensor Array Based on Dense Integration of Flexible 1-D Photonic Crystal Nanobeam Cavity With Large Free Spectral Range and High Q-Factor	10.1109/JPHOT.2017.2710136
478	Design and numerical analysis of highly sensitive Au-MoS2-graphene based hybrid surface plasmon resonance biosensor	10.1016/j.optcom.2017.03.035

№	TITLE_SCIENTIFIC PUBLICATION	DIGITAL OBJECT IDENTIFIER (DOI)
479	High sensitive and selective C-reactive protein detection by means of lossy mode resonance based optical fiber devices	10.1016/j.bios.2016.09.020
480	Design and optimization of silicon concentric dual-microring resonators for refractive index sensing	10.1016/j.optcom.2016.05.052
481	Plasmonic nanohole array biosensor for label-free and real-time analysis of live cell secretion	10.1039/C7LC00277G
482	Substrate Oxide Layer Thickness Optimization for a Dual-Width Plasmonic Grating for Surface-Enhanced Raman Spectroscopy (SERS) Biosensor Applications	10.3390/s17071530
483	Microcantilever array instrument based on optical fiber and performance analysis	10.1063/1.4994736
484	A novel method of SPR based SnO2: GNP nano-hybrid decorated optical fiber platform for hexachlorobenzene sensing	10.1016/j.snb.2017.02.123
485	Towards a Uniform Metrological Assessment of Grating-Based Optical Fiber Sensors: From Refractometers to Biosensors.	10.3390/bios7020023
486	Cancer biomarker sensing using packaged plasmonic optical fiber gratings: Towards in vivo diagnosis	10.1016/j.bios.2016.10.081
487	Optimal Design of Plasmonic Nanoslit Array Sensor Using Kriging Model	10.1109/LPT.2017.2702583
488	A SERS nano-tag-based fiber-optic strategy for in situ immunoassay in unprocessed whole blood	10.1016/j.bios.2016.10.070
489	Label-free detection of DNA hybridization with a compact LSPR-based fiber-optic sensor	10.1039/C7AN00249A
490	Simulation of the Sensing Performance of a Plasmonic Biosensor Based on Birefringent Solid-Core Microstructured Optical Fiber	10.1007/s11468-016-0342-y
491	Towards a Uniform Metrological Assessment of Grating-Based Optical Fiber Sensors: From Refractometers to Biosensors	10.3390/bios7020023
492	Optimization of Surface Plasmon Resonance Biosensor with Ag/Au Multilayer Structure and Fiber-Optic Miniaturization	10.1007/s11468-016-0312-4
493	Plasmonic Interference-Based Refractive Index Sensor Designed with Spectral Analysis and Structure Optimization	10.1007/s11468-016-0343-x
494	Theoretical analysis of microring resonator-based biosensor with high resolution and free of temperature influence	10.1117/1.OE.56.6.067103
495	Terahertz plasmon-induced transparency based on asymmetric dual-disk resonators coupled to a semiconductor InSb waveguide and its biosensor application	10.1117/1.OE.56.6.067109
496	Ultrasensitive and label -free detection of gamma-aminobutyric acid using fiber-optic interferometric sensors functionalized with size -selective molecular sieve arrays	___
497	Polymer Integrated Waveguide Optical Biosensor by Using Spectral Splitting Effect	10.1007/s13320-017-0395-3
498	Sensing with ultra-short Fabry-Perot cavities written into optical micro-fibers	10.1016/j.snb.2017.01.081
499	High-resolution and temperature-compensational HER2 antigen detection based on microwave photonic interrogation	10.1016/j.snb.2017.01.085
500	Mid-infrared T-shaped photonic crystal waveguide for optical refractive index sensing	10.1016/j.snb.2017.01.081
501	Nanoscale patterning of gold-coated optical fibers for improved plasmonic sensing	10.1088/1361-6528/aa6b53
502	Bio-sniffer (gas-phase biosensor) with secondary alcohol dehydrogenase (S-ADH) for determination of isopropanol in exhaled air as a potential volatile biomarker	10.1016/j.bios.2016.12.050

№	TITLE_SCIENTIFIC PUBLICATION	DIGITAL OBJECT IDENTIFIER (DOI)
503	High-sensitivity four-layer polymer fiber-optic evanescent wave sensor	10.1016/j.bios.2017.01.019
504	Recognition-mediated particle detection under microfluidic flow with waveguide-coupled 2D photonic crystals: towards integrated photonic virus detectors	10.1039/C7LC00221A
505	Development towards Compact Nitrocellulose-Based Interferometric Biochips for Dry Eye MMP9 Label-Free In-Situ Diagnosis	10.3390/s17051158
506	Stokes-Mueller matrix polarimetry system for glucose sensing	10.1016/j.optlaseng.2016.08.017
507	A wide-range temperature immune refractive-index sensor using concatenated long-period-fiber-gratings	10.1016/j.snb.2016.12.012
508	A novel U-bent plastic optical fibre local surface plasmon resonance sensor based on a graphene and silver nanoparticle hybrid structure	10.1088/1361-6463/aa628c
509	Sensing metabolites for the monitoring of tissue engineered construct cellularity in perfusion bioreactors	10.1016/j.bios.2016.09.094
510	Determination of High-affinity Antibody-antigen Binding Kinetics Using Four Biosensor Platforms	10.3791/55659
511	Highly sensitive optical biosensor based on silicon-microring-resonator-loaded Mach-Zehnder interferometer	10.7567/JJAP.56.04CH08
512	A Gelated Colloidal Crystal Attached Lens for Noninvasive Continuous Monitoring of Tear Glucose	10.3390/polym9040125
513	Integration of a microfluidic polymerase chain reaction device and surface plasmon resonance fiber sensor into an inline all-in-one platform for pathogenic bacteria detection	10.1016/j.snb.2016.10.137
514	A highly sensitive and distinctly selective D-sorbitol biosensor using SDH enzyme entrapped Ta2O5 nanoflowers assembly coupled with fiber optic SPR	10.1016/j.snb.2016.09.178
515	Simultaneous measurement of refractive index and temperature using a SPR-based fiber optic sensor	/10.1016/j.snb.2016.09.164
516	Interferometric detection of microRNAs using a capillary optofluidic sensor	10.1016/j.snb.2016.09.153
517	High Sensitivity Refractive Index Sensor Based on Multicoating Photonic Crystal Fiber With Surface Plasmon Resonance at Near-Infrared Wavelength	10.1109/JPHOT.2017.2687121
518	Thin cylindrical slot in an optical microdisk cavity for sensing biomaterials	10.1007/s00339-016-0745-9
519	Label-free protein sensing by employing blue phase liquid crystal	10.1364/BOE.8.001712
520	Cascaded ring resonator and Mach-Zehnder interferometer with a Sagnac loop for Vernier-effect refractive index sensing	10.1016/j.snb.2016.08.095
521	An array fluorescent biosensor based on planar waveguide for multi-analyte determination in water samples	10.1016/j.snb.2016.08.118
522	Label free ultrasensitive optical sensor decorated with polyaniline nanofibers: Characterization and immunosensing application	10.1016/j.snb.2016.08.103
523	Recent Progress in Polymer Optical Fiber Light Sources and Fiber Bragg Gratings	10.1109/JSTQE.2016.2616451
524	Aptamer- and Fab'- Functionalized Microring Resonators for Aflatoxin M1 Detection	10.1109/JSTQE.2016.2609100
525	Detection of extremely low concentration waterborne pathogen using a multiplexing self-referencing SERS microfluidic biosensor	10.1186/s13036-017-0051-x
526	Hybrid Graphene/Gold Plasmonic Fiber-Optic Biosensor	10.1002/admt.201600185
527	Functional magneto-plasmonic biosensors transducers: Modelling and nanoscale analysis	10.1016/j.snb.2016.07.128
528	One-port ring refractive index sensor with attached sub-ring	10.1007/s10043-016-0283-x
529	Multi-functional optical sensor based on plasmonic photonic liquid crystal fibers	10.1007/s11082-016-0849-7

№	TITLE_SCIENTIFIC PUBLICATION	DIGITAL OBJECT IDENTIFIER (DOI)
530	A novel nitrite biosensor based on the direct electrochemistry of horseradish peroxidase immobilized on porous Co3O4 nanosheets and reduced graphene oxide composite modified electrode	10.1016/j.snb.2016.07.073
531	One-Dimensional Silicon Nitride Grating Refractive Index Sensor Suitable for Integration With CMOS Detectors	10.1109/JPHOT.2016.2644962
532	Competitive inhibition assay for the detection of progesterone in dairy milk using a fiber optic SPR biosensor	10.1016/j.aca.2016.11.005
533	High Sensitivity Refractive Index Sensor Based on Highly Overcoupled Tapered Fiber-Optic Couplers	10.1109/JSEN.2016.2629281
534	Localized Surface Plasmon Resonance (LSPR)-Coupled Fiber-Optic Nanoprobe for the Detection of Protein Biomarkers	10.1007/978-1-4939-6848-0_1
535	D-glucose sensor using photonic crystal fiber	10.1016/j.ijleo.2017.08.039
536	The Optical Properties of Quantum Dots Integrated in a Hollow Core Photon Crystal Fiber	10.1117/12.2269378
537	Label-Free Biosensors Based on Bimodal Waveguide (BiMW) Interferometers	10.1007/978-1-4939-6848-0_11
538	Measuring Spatial and Temporal Oxygen Flux Near Plant Tissues Using a Self-Referencing Optrode	10.1007/978-1-4939-7292-0_23
539	SPR Based Hybrid Electro-Optic Biosensor for beta-Lactam Antibiotics Determination in Water	10.1117/12.2273318
540	Improved Sensitivity of Microring Resonator-Loaded Mach-Zehnder Interferometer Biosensor	10.18494/SAM.2017.1587
541	Precise sample preconcentration based on plasmon-assisted optical manipulation for a bead-based Raman biosensor	10.1117/12.2295661
542	Hopfield Neural Network and Optical Fiber Sensor as Intelligent Heart Rate Monitor	10.1117/12.2283012
543	The entangled photons generation in third order nonlinearity of spontaneous parametric down conversion by whispering gallery mode resonator	10.1117/12.2285365
544	Double-resonance long period fiber grating for detection of E. coli in trace concentration by choosing a proper bacteriophage	10.1117/12.2265950
545	Polyaniline deposition on tilted fiber Bragg grating for pH sensing	10.1117/12.2267524
546	Handheld highly selective plasmonic chem/biosensor using engineered binding proteins for extreme conformational changes	10.1117/12.2273207
547	High sensitive and selective Escherichia coli detection using immobilized optical fiber microprobe	10.1117/12.2263456
548	Trends in Fibre-Optic Uses for Personal Healthcare and Clinical Diagnostics	10.1007/978-3-319-42625-9_6
549	Sensitive Protein Detection and Quantification in Paper-Based Microfluidics for the Point of Care	10.1016/bs.mie.2017.01.018
550	Fabrication of Fiber Bragg Grating Coating with TiO2 Nanostructured Metal Oxide for Refractive Index Sensor	10.1155/2017/2791282
551	U-Bent Plastic Optical Fiber based Plasmonic Biosensor for Nucleic Acid Detection	10.1117/12.2265597
552	Evaluation of a Novel Label-Free Photonic-Crystal Biosensor Imaging System for the Detection of Prostate Cancer Cells	10.1117/12.2249630
553	High sensitive photonic crystal multiplexed biosensor array using H0 sandwiched cavities	10.1051/epjconf/201713900003
554	Monolithic integration of a plasmonic sensor with CMOS technology	10.1117/12.2250504
555	Modeling of a highly sensitive MoS2-Graphene hybrid based fiber optic SPR biosensor for sensing DNA hybridization	10.1016/j.ijleo.2017.05.001
556	Integration of Curved D-Type Optical Fiber Sensor with Microfluidic Chip	10.3390/s17010063
557	Wavelength-Scanning SPR Imaging Sensors Based on an Acousto-Optic Tunable Filter and a White Light Laser	10.3390/s17010090

№	TITLE_SCIENTIFIC PUBLICATION	DIGITAL OBJECT IDENTIFIER (DOI)
558	Fabricating 90 nm Resolution Structures in Sol-Gel Silica Optical Waveguides for Biosensor Applications	10.1155/2017/4198485
559	Disposable cartridge biosensor platform for portable diagnostics	10.1117/12.2254729
560	Silver-decorated silicon nanowires array as surface-enhanced Raman scattering (SERS) substrate	10.1117/12.2251332
561	Silicon nitride directional coupler interferometer for surface sensing	10.1117/1.OE.56.1.017101
562	Detection of Aeromonas hydrophila Using Fiber Optic Microchannel Sensor	10.1155/2017/8365189
563	Fiber optofluidic biosensor for the label-free detection of DNA hybridization and methylation based on an in-line tunable mode coupler	10.1016/j.bios.2016.06.060
564	Continuous Sensing Photonic Lab-on-a-Chip Platform Based on Cross-Linked Enzyme Crystals	10.1021/acs.analchem.6b03793
565	Temperature controlling fiber optic glucose sensor based on hydrogel-immobilized GOD complex	10.1016/j.snb.2016.06.062
566	Lattice plasmons in dielectric nanoparticle arrays arranged on metal film	10.1088/2040-8978/18/12/125002
567	An aptamer based method for small molecules detection through monitoring salt-induced AuNPs aggregation and surface plasmon resonance (SPR) detection	10.1016/j.snb.2016.06.035
568	Silicon-on-nitride slot waveguide: A promising platform as mid-IR trace gas sensor	10.1016/j.snb.2016.06.060
569	Full-Range Detection in Cascaded Microring Sensors Using Thermooptical Tuning	10.1109/JLT.2016.2608979
570	Optical weak measurement system with common path implementation for label-free biomolecule sensing	10.1364/OL.41.005409
571	Label-free bimodal waveguide immunosensor for rapid diagnosis of bacterial infections in cirrhotic patients	10.1016/j.bios.2016.04.095
572	Fluorescence based fiber optic and planar waveguide biosensors. A review	10.1016/j.aca.2016.08.049
573	A miniaturized oxygen sensor integrated on fiber surface based on evanescent-wave induced fluorescence quenching	10.1016/j.jlumin.2016.08.005
574	Surface plasmon resonance sensor based on photonic crystal fiber filled with core-shell Ag-Au nanocomposite materials	10.1117/1.OE.55.11.117104
575	Temperature Self-Compensated Optical Waveguide Biosensor Based on Cascade of Ring Resonator and Arrayed Waveguide Grating Spectrometer	10.1109/JLT.2016.2603338
576	Plasmonic spectrum on 1D and 2D periodic arrays of rod-shape metal nanoparticle pairs with different core patterns for biosensor and solar cell applications	10.1088/2040-8978/18/11/115003
577	Long-Range Surface Plasmon-Polariton Waveguide Biosensors for Disease Detection	opg.optica.org/jlt/abstract.cfm?URI=jlt-34-20-4673
578	Biosensing With Asymmetric High Refractive Index Contrast Gratings	10.1109/JSEN.2016.2599433
579	Biosensor for label-free DNA quantification based on functionalized LPGs	10.1016/j.bios.2015.10.001
580	Localized Surface Plasmon Resonance-Based Micro-Capillary Biosensor	10.1109/LPT.2016.2582202
581	Extraordinary Optical Transmission in a Hybrid Plasmonic Waveguide	10.1109/JPHOT.2016.2613599
582	Design of highly sensitive multichannel bimetallic photonic crystal fiber biosensor	10.1117/1.JNP.10.046016
583	Analysis and Design of Refractive Index Biosensors Based on Single Silicon Nanobeam Cavity	10.1109/JPHOT.2016.2614501
584	SPR-based plastic optical fibre biosensor for the detection of C-reactive protein in serum	10.1002/jbio.201500315

№	TITLE_SCIENTIFIC PUBLICATION	DIGITAL OBJECT IDENTIFIER (DOI)
585	Controlled chemical modification of the internal surface of photonic crystal fibers for application as biosensitive elements	10.1016/j.optmat.2016.07.046
586	Recent Advances on Luminescent Enhancement-Based Porous Silicon Biosensors	10.1007/s11095-016-1889-1
587	Investigation of Silicon Carbide Based Optical Fiber Coupled Surface Plasmon Resonance Sensor	10.1007/s12633-015-9394-4
588	Localized Plasmon-Based Optical Fiber Sensing Platform for Operation in Infrared	10.1109/LPT.2016.2573287
589	Gold-reinforced silver nanoprisms on optical fiber tapers-A new base for high precision sensing	10.1063/1.4953671
590	Design of a lithium niobate-on-insulator-based optical microring resonator for biosensing applications	10.1117/1.OE.55.8.087108
591	Carbon dots for naked eye colorimetric ultrasensitive arsenic and glutathione detection	10.1016/j.bios.2016.03.018
592	Label-free in-situ real-time DNA hybridization kinetics detection employing microfiber-assisted Mach-Zehnder interferometer	10.1016/j.bios.2016.02.065
593	Photonic crystal waveguide-based biosensor for detection of diseases	10.1117/1.JNP.10.036011
594	Active layer identification of photonic crystal waveguide biosensor chip for the detection of Escherichia coli	10.1117/1.OE.55.7.077105
	Optical fiber resonance-based pH sensors using gold nanoparticles into polymeric layer-by-layer coatings	10.1007/s00542-016-2857-8
596	Surface plasmon resonance based fiber optic ethanol sensor using layers of silver/silicon/hydrogel entrapped with ADH/NAD	10.1016/j.snb.2016.02.084
597	Dispersion curve-based sensitivity engineering for enhanced surface plasmon resonance detection	10.1016/j.optcom.2016.03.011
598	Long period fiber grating nano-optrode for cancer biomarker detection	10.1016/j.bios.2016.02.021
599	Probing the Localized Surface Plasmon Field of a Gold Nanoparticle-Based Fibre Optic Biosensor	10.1007/s11468-015-0106-0
600	Review of design principles of 2D photonic crystal microcavity biosensors in silicon and their applications	10.1007/s11468-015-0106-0
601	The Repetitive Detection of Toluene with Bioluminescence Bioreporter Pseudomonas putida TVA8 Encapsulated in Silica Hydrogel on an Optical Fiber	10.3390/ma9060467
602	SPR and SPR Imaging: Recent Trends in Developing Nanodevices for Detection and Real-Time Monitoring of Biomolecular Events	10.3390/s16060870
603	Homogeneous Biosensing Based on Magnetic Particle Labels	10.3390/s16060828
604	Self-Calibration Highly Sensitive Photonic Crystal Fiber Biosensor	10.1109/JPHOT.2016.2563319
605	SPR based fiber-optic sensor with enhanced electric field intensity and figure of merit using different single and bimetallic configurations	10.1016/j.optcom.2016.01.014
606	Refractive index change detection based on porous silicon microarray	10.1007/s00340-016-6405-0
607	Optically monitored drug delivery patch based on porous silicon and polymer microneedles	10.1364/BOE.7.001645
608	Evanescent Field Biosensor Using Polymer Slab Waveguide-Based Cartridges for the Optical Detection of Nanoparticles	10.1109/JSTQE.2015.2481080
609	Surface Plasmon Resonance Sensor for In Situ Detection of Xanthan Gum	10.1109/JSTQE.2015.2477054
610	Tracking micro-optical resonances for identifying and sensing novel procaspase-3 protein marker released from cell cultures in response to toxins	10.1088/0957-4484/27/16/164001

№	TITLE_SCIENTIFIC PUBLICATION	DIGITAL OBJECT IDENTIFIER (DOI)
611	Highly sensitive detection of urinary protein variations using tilted fiber grating sensors with plasmonic nanocoatings	10.1016/j.bios.2015.11.047
612	Designing a Biosensor Using a Photonic Quasi-Crystal Fiber	10.1109/JSEN.2016.2514850
613	Narrow band perfect absorber for maximum localized magnetic and electric field enhancement and sensing applications	10.1038/srep24063
614	Visualising apoptosis in live zebrafish using fluorescence lifetime imaging with optical projection tomography to map FRET biosensor activity in space and time	10.1002/jbio.201500258
615	Introduction of an angle interrogated, MEMS-based, optical waveguide grating system for label-free biosensing	10.1016/j.snb.2015.11.072
616	Plasmonic optical sensor for determination of refractive index of human skin tissues	10.1016/j.snb.2015.11.119
617	Cascaded Ring-Resonators for Multi-Channel Optical Sensing With Reduced Temperature Sensitivity	10.1109/LPT.2016.2514318
618	Blu-ray optomagnetic measurement based competitive immunoassay for Salmonella detection	10.1016/j.bios.2015.08.070
619	Developing localized surface plasmon resonance biosensor chips and fiber optics via direct surface modification of PMMA optical waveguides	10.1016/j.colsurfa.2015.11.025
620	Photonic biosensor based on photocorrosion of GaAs/AlGaAs quantum heterostructures for detection of Legionella pneumophila	10.1116/1.4941983
621	Progress in the research and development of photonic structure devices	10.1088/2043-6262/7/1/015003
622	Fiber optic and light scattering sensors: Complimentary approaches to rapid detection of Salmonella enterica in food samples	10.1016/j.foodcont.2015.09.031
623	Evanescent wave fluorescence biosensors: Advances of the last decade	10.1016/j.bios.2015.07.040
624	A Complete Optical Sensor System Based on a POF-SPR Platform and a Thermo-Stabilized Flow Cell for Biochemical Applications	10.3390/s16020196
625	Directional Coupler Biosensor Based on Thermooptic Detection Mechanism in Silica Waveguide	10.1109/JPHOT.2016.2520819
626	A SiON Microring Resonator-Based Platform for Biosensing at 850 nm	opg.optica.org/jlt/abstract.cfm?URI=jlt-34-3-969
627	A Novel Optical Biosensing System Using Mach-Zehnder-Type Optical Waveguide for Influenza Virus Detection	10.1007/s12010-015-1902-x
628	Multichannel photonic crystal fiber surface plasmon resonance based sensor	10.1007/s11082-016-0414-4
629	A Localized Surface Plasmon Resonance-Based Portable Instrument for Quick On-Site Biomolecular Detection	10.1109/TIM.2015.2465691
630	Biosensors and bioelectronics on smartphone for portable biochemical detection	10.1016/j.bios.2015.08.037
631	Temperature-Compensating Fiber-Optic Surface Plasmon Resonance Biosensor	10.1109/LPT.2015.2492603
632	Design optimization of long period waveguide grating devices for refractive index sensing using adaptive particle swarm optimization	10.1016/j.optcom.2015.09.086
633	Evanescent wave absorption based S-shaped fiber-optic biosensor for immunosensing applications	10.1016/j.proeng.2016.11.161
634	Optical biosensors	10.1021/cr068105t
635	Bloch surface wave structures for high sensitivity detection and compact waveguiding	10.1080/14686996.2016.1202082
636	Modular Optofluidic Systems (MOPS)	10.1117/12.2242997
637	Immunogold-silver staining (IGSS) based U-bent fiberoptic sandwich biosensor	10.1117/12.2242980
638	Slotted-core photonic crystal fiber in gas sensing application	10.1117/12.2247753

№	TITLE_SCIENTIFIC PUBLICATION	DIGITAL OBJECT IDENTIFIER (DOI)
639	Micro-capillary-Based Self-Referencing Surface Plasmon Resonance Fiber-Optic Biosensor	10.1117/12.2245948
640	Porous silicon-based two-dimensional photonic crystal for biochemical sensing applications	10.1117/12.2246120
641	Gold nano sphere based fiber optic LSPR probe for biosensing measurement	10.1117/12.2245960
642	Improved Optical Side Coupling Efficiency by Spiral Patterned Zinc Oxide Nanorod Coatings on Large Core Plastic Optical Fiber	10.1117/12.2241640
643	Investigation on optical properties of BSA protein on single-layer graphene using terahertz spectroscopy technology	10.1117/12.2246258
644	Nanohybrids Near-Field Optical Microscopy: From Image Shift to Biosensor Application	10.1155/2016/4089260
645	Optical waveguide materials, structures, and dispersion modulation	10.1117/12.2249446
646	Silicon chip integrated Photonic Sensors for Biological and Chemical Sensing	10.1117/12.2223240
647	Paper-based sensors and assays: a success of the engineering design and the convergence of knowledge areas	10.1039/C6LC00737F
648	Compact surface plasmon resonance biosensor utilizing an injection-molded prism	10.1117/12.2223682
649	Engineering molecularly-active nanoplasmonic surfaces for DNA detection via colorimetry and Raman scattering	10.1117/12.2209108
650	Integrated Plasmonic Refractive Index Sensor based on Grating/Metal Film Resonant Structure	10.1117/12.2218558
651	Novel Highly Sensitive Protein Sensors Based on Tapered Optical Fibres Modified with Au-Based Nanocoatings	10.1155/2016/8129387
652	Enhancement of effective quality-factor using asymmetric Mach-Zehnder interferometer with ring resonator for optical bio and chemical sensor	10.1117/12.2212334
653	Influence of the sensitivity of an optical resonator with a surface layer by its properties	10.1117/12.2208542
654	An embedded microretroreflector-based microfluidic immunoassay platform	10.1039/C6LC00038J
655	Novel localized surface plasmon resonance based optical fiber sensor	10.1117/12.2212652
656	Improved sensitivity of the photonic crystal slab biosensors by using elliptical air holes	10.1016/j.ijleo.2016.03.057
657	Effects from detuning the resonant coupling between fiber gratings and localized surface plasmons	10.1117/12.2234134
658	Study on the effect of nanoparticle bimetallic coreshell Au-Ag for sensitivity enhancement of biosensor based on surface plasmon resonance	10.1088/1742-6596/694/1/012075
659	Recent advances in optical fiber devices for microfluidics integration	10.1002/jbio.201500170
660	Surface plasmon resonance biosensor based on large size square-lattice photonic crystal fiber	10.1080/09500340.2015.1102345
661	High-sensitivity plasmonic sensor based on perfect absorber with metallic nanoring structures	10.1080/09500340.2015.1066459
662	Modulational instability of polarization of light in a periodically poled lithium niobate chip	10.1117/1.OE.55.1.017105
663	Cladding modes in photonic crystal fiber: characteristics and sensitivity to surrounding refractive index	10.1117/1.OE.55.1.017106
664	A reflective mirror microcantilevers-based biosensor for biochemical detection	10.1016/j.ijleo.2015.11.103
665	Integrated Photonic Nanofences: Combining Subwavelength Waveguides with an Enhanced Evanescent Field for Sensing Applications	10.1021/acsnano.5b05864

№	TITLE_SCIENTIFIC PUBLICATION	DIGITAL OBJECT IDENTIFIER (DOI)
666	Organic Semiconductor Laser Biosensor: Design and Performance Discussion	10.1109/JSTQE.2015.2448058
667	Interferometric-type optical biosensor based on exposed core microstructured optical fiber	10.1016/j.snb.2015.06.068
668	A polydopamine-modified optical fiber SPR biosensor using electroless-plated gold films for immunoassays	10.1016/j.bios.2015.06.080
669	Monolithically integrated broad-band Mach-Zehnder interferometers for highly sensitive label-free detection of biomolecules through dual polarization optics	10.1038/srep17600
670	Micronanofabrication for optofluidic sensors	10.3938/jkps.67.1992
671	Label-free Single Molecule Detection Using Microtoroid Optical Resonators	10.1038/lsa.2016.1
672	Highly sensitive color-indicating and quantitative biosensor based on cholesteric liquid crystal	10.1364/BOE.6.005033
673	Immobilization of cholesterol oxidase on magnetic fluorescent core-shell-structured nanoparticles	10.1016/j.msec.2015.07.038
674	Athermal optical waveguide microring biosensor with intensity interrogation	10.1016/j.optcom.2015.07.036
675	Nanoparticle-based lateral flow biosensors	10.1016/j.bios.2015.05.050
676	An acetone bio-sniffer (gas phase biosensor) enabling assessment of lipid metabolism from exhaled breath	10.1016/j.bios.2015.04.023
677	Ultrasensitive and Specific Measurement of Protease Activity Using Functionalized Photonic Crystals	10.1021/acs.analchem.5b02529
678	On the Performance of Graphene-Based D-Shaped Photonic Crystal Fibre Biosensor Using Surface Plasmon Resonance	10.1007/s11468-015-9912-7
679	Graphene Enhances the Sensitivity of Fiber-Optic Surface Plasmon Resonance Biosensor	10.1109/JSEN.2015.2442276
680	PMMA MICROFIBER COATED WITH AL-DOPED ZNO NANOSTRUCTURES FOR DETECTING URIC ACID	10.1016/j.measurement.2016.12.021
681	High-Sensitivity Biochemical Sensor Based on Cylindrical Nano-Metal Particles Array	opg.optica.org/jlt/abstract.cfm?URI=jlt-33-17-3635
682	Sensitivity analysis for improving nanomechanical photonic transducers biosensors	10.1088/0022-3727/48/33/335401
683	A low cost surface plasmon resonance biosensor using a laser line generator	10.1016/j.optcom.2015.03.035
684	Beta-Sheet-Forming, Self-Assembled Peptide Nanomaterials towards Optical, Energy, and Healthcare Applications	10.1002/smll.201500169
685	Surface Plasmon Resonance Biosensor Based on Smart Phone Platforms	10.1038/srep12864
686	PEG Functionalization of Whispering Gallery Mode Optical Microresonator Biosensors to Minimize Non-Specific Adsorption during Targeted, Label-Free Sensing	10.3390/s150818040
687	Selective detection of bacteria in urine with a long-range surface plasmon waveguide biosensor	10.1364/BOE.6.002908
688	Nanoporous silicon microcavity based optical sensor to detect adulteration of petrol by organic solvents	10.1007/s11082-014-0107-9
689	Absorption enhancement and sensing properties of Ag diamond nanoantenna arrays	10.1088/1674-1056/24/7/074206
690	Reusable Bacteriophage Adhesin-Coated Long-Period Grating Sensor for Bacterial Lipopolysaccharide Recognition	opg.optica.org/jlt/abstract.cfm?URI=jlt-33-12-2518
691	Polarimetric Plasmonic Sensing with Bowtie Nanoantenna Arrays	10.1007/s11468-014-9856-3
692	Experimental Validation of the Sensitivity of Waveguide Grating Based Refractometric (Bio) sensors	10.3390/bios5020187
693	An Optical Biosensor from Green Fluorescent Escherichia coli for the Evaluation of Single and Combined Heavy Metal Toxicities	10.3390/s150612668

№	TITLE_SCIENTIFIC PUBLICATION	DIGITAL OBJECT IDENTIFIER (DOI)
694	Label-free biosensing using cascaded double-microring resonators integrated with microfluidic channels	10.1016/j.optcom.2015.01.028
695	Recognition of bacterial lipopolysaccharide using bacteriophage-adhesin-coated long-period gratings	10.1016/j.bios.2014.07.027
696	Picosecond optically reconfigurable filters exploiting full free spectral range tuning of single ring and Vernier effect resonators	10.1364/OE.23.012468
697	Review of plasmonic fiber optic biochemical sensors: improving the limit of detection	10.1007/s00216-014-8411-6
698	Angularly resolved ellipsometric optical biosensing by means of Bloch surface waves	10.1007/s00216-015-8591-8
699	Tube Glass Waveguides Modified With Gold Nanoparticles for Application as a Simple Chemical and Biological Sensor	10.1109/JSEN.2014.2381234
700	A reusable aptamer-based evanescent wave all-fiber biosensor for highly sensitive detection of Ochratoxin A	10.1016/j.bios.2014.10.079
701	Optical Nano Antennas: State of the Art, Scope and Challenges as a Biosensor Along with Human Exposure to Nano-Toxicology	10.3390/s150408787
702	Multiplex Serum Cytokine Immunoassay Using Nanoplasmonic Biosensor Microarrays	10.1021/acsnano.5b00396
703	Tunable Plasmonic Sensor With Metal-Liquid Crystal-Metal Structure	10.1109/JPHOT.2015.2411214
704	Optical biosensor based on a silicon nanowire ridge waveguide for lab on chip applications	10.1088/2040-8978/17/4/045802
705	Design of grating couplers and MMI couplers on the TriPleX platform enabling ultra-compact photonic-based biosensors	10.1016/j.snb.2014.11.098
706	Fabrication of long-range surface plasmon-polariton Bragg gratings with microfluidic channels in Cytop claddings	10.1016/j.mee.2015.03.001
707	Optical surface plasmon resonance biosensors in molecular fishing	10.18097/PBMC20156102231
708	Linear readout of integrated interferometric biosensors using a periodic wavelength modulation	10.1002/lpor.201400216
709	Sensitive detection of 2,4,6-trinitrotoluene by tridimensional monitoring of molecularly imprinted polymer with optical fiber and five-branched gold nanostars	10.1016/j.snb.2014.10.079
710	Numeric Tuning of Surface Plasmon Enhanced Spontaneous Emission Inducted by Nano-Metallic Particle Systems Embedded in GaN-Based LED	10.1109/JDT.2014.2385592
711	Gold Elliptic Nanocavity Array Biosensor With High Refractive Index Sensitivity Based on Two-Photon Nanolithography	10.1109/JPHOT.2014.2366167
712	Efficient optical biochemical sensor with slotted Bragg-grating-based Fabry-Perot resonator structure in silicon-on-insulator platform	10.1007/s11082-014-9908-0
713	On-line biosensor for the detection of putative toxicity in water contaminants	10.1016/j.talanta.2014.09.032
714	Magneto-plasmonic biosensor with enhanced analytical response and stability	10.1016/j.bios.2014.08.004
715	Detection of unamplified genomic DNA by a PNA-based microstructured optical fiber (MOF) Bragg-grating optofluidic system	10.1016/j.bios.2014.07.047
716	МЭМС ДЛЯ ДИАГНОСТИКИ ВОЗБУДИТЕЛЕЙ ЗАБОЛЕВАНИЙ	ISNN:1813-8586
717	Swallowable fluorometric capsule for wireless triage of gastrointestinal bleeding	10.1039/c5lc00770d
718	A Fluorometric Biochemical Gas Sensor (Biosniffer) for Acetaldehyde Vapor Based on Catalytic Reaction of Aldehyde Dehydrogenase	ISSN: 0914-4935
719	A tunable submicro-optofluidic polymer filter based on guided-mode resonance	10.1039/C4NR07233B

№	TITLE_SCIENTIFIC PUBLICATION	DIGITAL OBJECT IDENTIFIER (DOI)
720	A novel single-layered MoS2 nanosheet based microfluidic biosensor for ultrasensitive detection of DNA	10.1039/C4NR07162J
721	3D plasmonic nanoantennas integrated with MEA biosensors	10.1039/C4NR05578K
722	A regenerative label-free fiber optic sensor using surface plasmon resonance for clinical diagnosis of fibrinogen	10.2147/IJN.S88963
723	Detecting single DNA molecule interactions with optical microcavities	10.1117/12.2190649
724	Design and optimization of diamond-shaped biosensor using photonic crystal nano-ring resonator	10.1016/j.ijleo.2015.06.037
725	Electromagnetic resonances and their tunability in planar metamolecules isomer	10.1016/j.ijleo.2015.07.033
726	Surface passivation of a photonic crystal band-edge laser by atomic layer deposition of SiO2 and its application for biosensing	10.1039/C4NR07552H
727	Lab-on-Fiber biosensing for cancer biomarker detection	10.1117/12.2194943
728	Trends in Biosensors for HPV: Identification and Diagnosis	10.1155/2015/913640
729	Fiber-optic triggered release of liposome in vivo: implication of personalized chemotherapy	10.2147/IJN.S85915
730	Optical biosensor technologies for molecular diagnostics at the point-of-care	10.1117/12.2185642
731	Engineering optical near-fields for highly efficient surface-enhanced nanoplasmonics	10.1117/12.2189754
732	Enhancing enzymatic efficiency by attachment to semiconductor nanoparticles for biosensor applications	10.1117/12.2180645
733	Modeling and performance analysis of chalcogenide prism based plasm onic biosensor comprising of gold nanoparticle film	10.1117/12.2178849
734	Efficient nanoplasmonic antennas for fabricating single protein molecule detector	10.1117/12.2182874
735	Optical fiber pH sensor based on gold nanoparticles into polymeric coatings	10.1117/12.2179970
736	Cancer-cells on a chip for label-free optic detection of secreted molecules	10.1117/12.2179883
737	Photoluminescence characterization of ZnO nanowires functionalization	10.1117/12.2178593
738	Influence of the permeable layer number of porous silicon microcavity on reflection spectrum	10.1016/j.ijleo.2015.01.026
739	A reusable biosensor chip for SERS-fluorescence dual mode immunoassay	10.1117/12.2180410
740	A Multiplexed Label Free Plasmonic Nano-Device for Near Infrared Applications	10.1063/1.4908581
741	Low-cost high performance readout system for fiber-optic biosensors	10.1117/12.2080209
742	Theoretical study on sensing performance of hydrogen annealed silicon waveguides	10.1080/09500340.2014.975846
743	Fiber-optic sensors for high throughput screening of pathogens	10.1016/B978-0-85709-801-6.00010-1
744	Analyzing the biosensor signal in flows: Studies with glucose optrodes	10.1016/j.talanta.2014.07.061
