# Supplementary material for: A review of Optical Point-of-Care devices to Estimate the Technology Transfer of These Cutting-Edge Technologies
Source: Biosensors (Basel). 2022 Nov 29;12(12):1091. doi: 10.3390/bios12121091 (PMC9776401; doi:10.3390/bios12121091)
Supplement: Supplementary file 1 [file biosensors-12-01091-s001.zip › Table S2 Suppl_File.pdf]

| APPENDIX B_ LIST OF EXCLUDED ARTICLES | | |
|---|---|---|
| **Nº** | **TITLE_SCIENTIFIC PUBLICATION** | **DIGITAL OBJECT IDENTIFIER (DOI)** |
| 1 | Real-time interrogation of fiber optic biosensor using TiO2 coated etched long-period grating | 10.1063/5.0020571 |
| 2 | Recent Advances in the Fabrication and Application of Graphene Microfluidic Sensors | 10.3390/mi11121059 |
| 3 | Design and analysis of electro-optic modulators based on high contrast gratings in AlGaN/GaN heterostructures | 10.1088/1361-6641/abbc8d |
| 4 | Modelling and simulation of novel liquid-infiltrated PCF biosensor in Terahertz frequencies | 10.1049/iet-opt.2020.0069 |
| 5 | Etched multicore fiber sensor using copper oxide and gold nanoparticles decorated graphene oxide structure for cancer cells detection | 10.1016/j.bios.2020.112557 |
| 6 | A switchable split ring resonator nanoantenna design with organic material composite as a refractive index sensor | 10.1016/j.optcom.2020.126211 |
| 7 | Sensitivity enhancement of WS2-coated SPR-based optical fiber biosensor for detecting glucose concentration* | 10.1117/12.2026061 |
| 8 | Detection of cardiac troponin-I by optic biosensors with immobilized anti-cardiac troponin-I monoclonal antibody | 10.1016/j.talanta.2020.121259 |
| 9 | Recent advance in biosensing applications based on two-dimensional transition metal oxide nanomaterials | 10.1016/j.talanta.2020.121308 |
| 10 | Label-free biosensor array comprised of Vernier microring resonator and 3x3 optical coupler | 10.1140/epjp/s13360-020-00888-6 |
| 11 | Numerical analysis of Bragg grating-based slot-micro-ring coupling resonator system for electromagnetically-induced transparency-like effect | 10.1007/s11082-020-02555-7 |
| 12 | The Measurement of Nanoparticle Concentrations by the Method of Microcavity Mode Broadening Rate | 10.3390/s20205950 |
| 13 | All fiber, highly sensitive sensor based on gold nanoparticle-coated macrobent single mode fiber for human temperature monitoring | 10.1117/1.JNP.14.046013 |
| 14 | Design of D-Shaped PCF-SPR sensor with dual coating of ITO and ZnO conducting metal oxide | 10.1016/j.ijleo.2020.165135 |
| 15 | CMOS-Compatible Silicon Photonic Sensor for Refractive Index Sensing Using Local Back-Side Release | 10.1109/LPT.2020.3019114 |
| 16 | Plasmonic Sensors for Monitoring Biological and Chemical Threat Agents | 10.3390/bios10100142 |
| 17 | Detection of L-Cysteine Using Silver Nanoparticles and Graphene Oxide Immobilized Tapered SMS Optical Fiber Structure | 10.1109/JSEN.2020.2997690 |
| 18 | Reflector-less nanoparticles doped optical fiber biosensor for the detection of Case thrombin | 10.1016/j.bios.2020.112365 |
| 19 | Numerical analysis of gold coating based quasi D-shape dual core PCF SPR sensor | 10.1007/s11082-020-02555-7 |
| 20 | Nanomaterial-Enhanced Fiber Optofluidic Laser Biosensor for Sensitive Enzyme Detection | 10.1109/JLT.2020.2997993 |
| 21 | Behaviour of Poynting vector for dielectric-metal-dielectric optical waveguides and applications | 10.1007/s11082-020-02519-x |
| 22 | Optically Tunable Triple-Band Perfect Absorber for Nonlinear Optical Liquids Sensing | 10.1109/JSEN.2020.2989742 |
| 23 | All-Dielectric Metasurface Fluorescence Biosensors for High-Sensitivity Antibody/Antigen Detection | 10.1021/acsnano.0c07722 |
| 24 | Fiber optic particle plasmon resonance immunosensor for rapid and sensitive detection of methamphetamine based on competitive inhibition | 10.1016/j.microc.2020.105026 |
| 25 | Theoretical Analysis of a Simultaneous Graphene-Based Circular Plasmonic Refractive Index and Thickness Bio-Sensor | 10.1109/JSEN.2020.2987696 |

| № | TITLE_SCIENTIFIC PUBLICATION | DIGITAL OBJECT IDENTIFIER (DOI) |
|---|---|---|
| 26 | Ultrasensitive optical biosensors based on microresonators with bent waveguides | 10.1016/j.ijleo.2020.164906 |
| 27 | An Integrated, Optofluidic System With Aligned Optical Waveguides, Microlenses, and Coupling Prisms for Fluorescence Sensing | 10.1109/JMEMS.2020.3004374 |
| 28 | Two-photon fluorescence-assisted laser ablation of non-planar metal surfaces: fabrication of optical apertures on tapered fibers for optical neural interfaces | 10.1364/OE.395187 |
| 29 | Polarization Selectivity of the Thin-Metal-Film Plasmon-Assisted Fiber-Optic Polarizer | 10.1021/acsami.0c08274 |
| 30 | Numerical analysis of effective refractive index bio-sensor based on graphene-embedded slot-based dual-micro-ring resonator | 10.1142/S0217979220501453 |
| 31 | Hollow-core graded index optical fiber refractive index sensor based on surface plasmon resonance | 10.1007/s11082-020-02461-y |
| 32 | Hollow core photonic crystal fiber-assisted Raman spectroscopy as a tool for the detection of Alzheimer's disease biomarkers | 10.1117/1.JBO.25.7.077001 |
| 33 | Design and Realization of a Novel Poly-Silicon Light-Emitting Device Based on Standard CMOS Technology | 10.3788/CJL202047.0701027 |
| 34 | Highly sensitive fiber optic surface plasmon resonance sensor employing 2D nanomaterials | 10.1007/s00339-020-03712-1 |
| 35 | Enhancement of ultrathin localized surface plasmon resonance sensitivity using sequential temperature treatment for liquids sensing | 10.1016/j.sna.2020.112006 |
| 36 | An Analysis of a Compact Label-Free Guiding-Wave Biosensor Based on a Semiconductor-Clad Dielectric Strip Waveguide | 10.3390/s20123368 |
| 37 | Two Modes Excited SPR Sensor Employing Gold-Coated Photonic Crystal Fiber Based on Three-Layers Air-Holes | 10.1109/JSEN.2020.2972031 |
| 38 | Recent advances in fiber-optic evanescent wave sensors for monitoring organic and inorganic pollutants in water | 10.1016/j.trac.2020.115892 |
| 39 | A novel surface plasmon based photonic crystal fiber sensor | 10.1007/s11082-020-02403-8 |
| 40 | Biophotonic sensor for rapid detection of brain lesions using 1D photonic crystal | 10.1007/s11082-020-02409-2 |
| 41 | Critical assessment of relevant methods in the field of biosensors with direct optical detection based on fibers and waveguides using plasmonic, resonance, and interference effects | 10.1109/JLT.2020.2969016 |
| 42 | Designing a dual steering wheel microstructured blood components sensor in terahertz wave band | 10.1117/1.OE.59.4.047104 |
| 43 | Thermally Stable Optical Filtering Using Silicon-Based Comb-Like Asymmetric Grating for Sensing Applications | 10.1109/JSEN.2019.2960604 |
| 44 | Advancements in SPR biosensing technology: An overview of recent trends in smart layers design, multiplexing concepts, continuous monitoring and in vivo sensing | 10.1016/j.aca.2019.12.067 |
| 45 | Coupling between surface plasmon polariton and planar waveguide modes in the biosensor based on metal-insulator-metal/planar waveguide structure | 10.1016/j.optcom.2019.124928 |
| 46 | The Antibody-Free Recognition of Cancer Cells Using Plasmonic Biosensor Platforms with the Anisotropic Resonant Metasurfaces | 10.1016/j.optlastec.2019.105922 |
| 47 | Highly sensitive sensor based on D-shaped microstructure fiber with hollow core | 10.1016/j.optlastec.2019.105922 |
| 48 | A high figure of merit refractive index sensor based on Fano resonance in all-dielectric metasurface | 10.1016/j.rinp.2019.102833 |
| 49 | Low-Cost and Highly Sensitive Liquid Refractive Index Sensor Based on Polymer Horizontal Slot Waveguide | 10.1007/s13320-019-0560-y |

| № | TITLE_SCIENTIFIC PUBLICATION | DIGITAL OBJECT IDENTIFIER (DOI) |
|---|---|---|
| 50 | An Integrated Biological Analysis and Flow Rate Sensing for the Real-Time Detection of Carcinogen in Water Based on Co2+-Doped Optical Fibers | 10.1109/JSEN.2019.2948964 |
| 51 | Study on the sensing characteristics of Fano resonance based on a coupled streamlined resonance cavity | 10.1364/OSAC.377612 |
| 52 | Introducing waveguide loss: Another way to realize a high-sensitivity microring biosensor | 10.1016/j.ijleo.2019.163339 |
| 53 | Multi-Channel Photonic Crystal Fiber Based Surface Plasmon Resonance Sensor for Multi-Analyte Sensing | 10.1109/JPHOT.2019.2961110 |
| 54 | Diphenylalanine Nanotube Coated Fiber Bragg Grating for Methanol Vapor Detection | 10.1109/JSEN.2019.2946123 |
| 55 | ? Dual-polarized optical sensing of microstructure fiber with pentagonal-lattice based on surface plasmon resonance in the near-IR spectrum | 10.1016/j.ijleo.2019.163671 |
| 56 | ? Optical properties of fluidic defect states in one-dimensional graphene-based photonic crystal biosensors: visible and infrared Hall regime sensing | 10.1140/epjp/s13360-019-00056-5 |
| 57 | General formulae of designing novel photonic digital sensors with arbitrary levels of directional couplers for testing liquids | 10.1007/s11082-019-2177-1 |
| 58 | The interaction mechanism between bovine serum albumin and single-walled carbon nanotubes depending on their diameter and concentration in solid bionanocomposites | 10.1117/12.2554935 |
| 59 | Fiber Optic Biosensors: Types, Optical Parameters, Applications and Future Scope | 10.1063/5.0001791 |
| 60 | Recent Advancements In Fiber-Optics Biosensors | 10.1063/5.0001245 |
| 61 | Self-assembled glass-based Fano resonant metasurfaces | 10.1117/12.2546112 |
| 62 | ? Synthesis of TiO2 nanoparticles decorated with gold nanoclusters using pulsed laser ablation in liquid | 10.1070/QEL17318 |
| 63 | Optimally designed narrowband guided-mode resonance transmittance filters for label-free optical biosensor | 10.1117/12.2545941 |
| 64 | Versatile Bio-Organism Detection using Microspheres for Future Biodegradation and Bioremediation Studies | 10.1117/12.2543800 |
| 65 | Fabrication of multichannel Bloch long range surface plasmon biosensors | 10.1117/12.2545106 |
| 66 | Low-cost glucose biosensor fabricated by a photosensitive resin that features nanoparticles | 10.1117/12.2546938 |
| 67 | Bioengineered Polymer/Composites as Advanced 'Biological Detection Sorbitol: An Application in Healthcare Sector | 10.2174/1568026620666200306131416 |
| 68 | Sensitivity Enhancement in Long Period Gratings by Mode Transition in Uncoated Double Cladding Fibers | 10.1016/j.sna.2013.01.012 |
| 69 | Modulation of localized surface plasmon resonance of the titanium nitride nanoparticle array based on graphene | 10.1016/j.ijleo.2019.163420 |
| 70 | Quantitative remote and on-site Hg2+ detection using the handheld smartphone based optical fiber fluorescence sensor (SOFFS) | 10.1016/j.snb.2019.127168 |

| № | TITLE_SCIENTIFIC PUBLICATION | DIGITAL OBJECT IDENTIFIER (DOI) |
|---|---|---|
| 71 | ? Dual-polarized ultrahigh sensitive gold/MoS2/graphene based D-shaped PCF refractive index sensor in visible to near-IR region | 10.1007/s11082-019-2122-3 |
| 72 | Design and investigation of PCF-based blood components sensor in terahertz regime | 10.1007/s00339-019-3164-x |
| 73 | Ultra-sensitive photonic crystal cancer cells sensor with a high-quality factor | 10.1016/j.cryogenics.2019.102991 |
| 74 | Ultrasensitive biosensor based on magnetic microspheres enhanced microfiber interferometer | /10.1016/j.bios.2019.111563 |
| 75 | Ultrasensitive biosensor based on magnetic microspheres enhanced microfiber interferometer | 10.1016/j.bios.2019.111563 |
| 76 | Size dependent plasmonic properties of Ga@Ag & Cs@Ag liquid-metal nanospheres | 10.1016/j.optcom.2019.07.049 |
| 77 | Design of a High-Resolution Metal-Insulator-Metal Plasmonic Refractive Index Sensor Based on a Ring-Shaped Si Resonator | 10.1007/s11468-019-00926-9 |
| 78 | Design and Performance of a Multipurpose 2-D Photonic Crystal Device Based on Y Couplers | 10.1007/s11468-019-00934-9 |
| 79 | Circular-Pattern Photonic Crystal Fiber for Different Liquids with High Effective Area and Sensitivity | 10.1007/s11468-019-00977-y |
| 80 | Metal Nanowire Assisted Hollow Core Fiber Sensor for an Efficient Detection of Small Refractive Index Change of Measurand Liquid | 10.1007/s11468-019-00969-y |
| 81 | Development of an enzyme-based fiber optic biosensor for detection of haloalkanes | 10.1108/SR-01-2019-0001] |
| 82 | Demonstration of an optical biosensor for the detection of faecal indicator bacteria in freshwater and coastal bathing areas | 10.1007/s00216-019-02182-6 |
| 83 | Surface-enhanced Raman scattering-active photonic crystal fiber probe: Towards next generation liquid biopsy sensor with ultra high sensitivity | 10.1002/jbio.201900027 |
| 84 | A Review: Evolution and Diversity of Optical Fibre Plasmonic Sensors | 10.3390/s19224874 |
| 85 | Optical biosensors based on refractometric sensing schemes: A review | 10.1016/j.bios.2019.111693 |
| 86 | The application of porous Si photonic crystals for metal-resonance enhanced fluorescence | 10.1007/s11801-019-9035-z |
| 87 | Vertical silicon nanowire-based racetrack resonator optical sensor | 10.1007/s00339-019-2942-9 |
| 88 | An Analytical Approach to Predict Maximal Sensitivity of Microring Resonators for Absorption Spectroscopy | 10.1109/JLT.2019.2938040 |
| 89 | Slow light enhanced bio sensing properties of silicon sensors | 10.1007/s11082-019-2067-6 |
| 90 | Biosensors for early diagnosis of pancreatic cancer: a review | 10.1016/j.trsl.2019.08.002 |
| 91 | Plasmonic-3D photonic crystals microchip for surface enhanced Raman spectroscopy | 10.1016/j.bios.2019.111596 |
| 92 | Polariton nanophotonics using phase-change materials | 10.1038/s41467-019-12439-4 |

| № | TITLE_SCIENTIFIC PUBLICATION | DIGITAL OBJECT IDENTIFIER (DOI) |
|---|---|---|
| 93 | High-Performance Label-Free Near-Infrared SPR Sensor for Wide Range of Gases and Biomolecules Based on Graphene-Gold Grating | 10.1007/s11468-019-00906-z |
| 94 | Tetra-core surface plasmon resonance based biosensor for alcohol sensing | 10.1016/j.physb.2019.05.047 |
| 95 | Enhancing the evanescent field in TiO2/Au hybrid thin films creates a highly sensitive room-temperature formaldehyde gas biosensor | 10.1016/j.colsurfb.2019.06.033 |
| 96 | Wearable flexible sweat sensors for healthcare monitoring: a review | 10.1098/rsif.2019.0217 |
| 97 | Surface plasmon resonance (SPR) biosensors for food allergen detection in food matrices | 10.1016/j.bios.2019.111449 |
| 98 | Photonic crystal slab biosensors fabricated with helium ion lithography (HIL) | 10.1016/j.sna.2019.07.017 |
| 99 | Optical Interrogation Techniques for Nanophotonic Biochemical Sensors | 10.3390/s19194287 |
| 100 | Current status of optical fiber biosensor based on surface plasmon resonance | 10.1016/j.bios.2019.111505 |
| 101 | Graphene Nanoribbon Assisted Refractometer Based Biosensor for Mid-Infrared Label-Free Analysis | 10.1007/s11468-019-00909-w |
| 102 | Highly Sensitive Dual-Core PCF Based Plasmonic Refractive Index Sensor for Low Refractive Index Detection | 10.1109/JPHOT.2019.2931713 |
| 103 | Water-Induced Fused Silica Glass Surface Alterations Monitored Using Long-Period Fiber Gratings | 10.1109/JLT.2019.2909947 |
| 104 | Sensitivity comparison of surface plasmon resonance (SPR) and magneto-optic SPR biosensors | 10.1140/epjp/i2019-12819-3 |
| 105 | Refractive index sensitivity enhancement of optical fiber SPR sensor utilizing layer of MWCNT/PtNPs composite | 10.1016/j.yofte.2019.05.007 |
| 106 | Fast fluorometric enumeration of E. coli using passive chip | 10.1016/j.mimet.2019.105680 |
| 107 | A Bimetallic-Coated, Low Propagation Loss, Photonic Crystal Fiber Based Plasmonic Refractive Index Sensor | 10.3390/s19173794 |
| 108 | Optimizing the Limit of Detection of Waveguide-Based Interferometric Biosensor Devices | 10.3390/s19173671 |
| 109 | Bio-Recognition in Spectroscopy-Based Biosensors for *Heavy Metals-Water and Waterborne Contamination Analysis | 10.3390/bios9030096 |
| 110 | Suspended 3D AgNPs/CNT nanohybrids for the SERS application | 10.1016/j.apsusc.2019.05.179 |
| 111 | Fiber optic surface plasmon resonance biosensor for detection of PDGF-BB in serum based on self-assembled aptamer and antifouling peptide monolayer | 10.1016/j.bios.2019.111350 |
| 112 | Plasmonic Multiplexing Extraordinary Optical Transmission Sensor With Enhanced Sensitivity Through Grooves in Metal Film | 10.1109/JSEN.2019.2914962 |
| 113 | Graphene-based nanocomposites for sensitivity enhancement of surface plasmon resonance sensor for biological and chemical sensing: A review | 10.1016/j.bios.2019.111324 |
| 114 | MyoRobot 2.0: An advanced biomechatronics platform for automated, environmentally controlled skeletal muscle single fiber biomechanics assessment employing inbuilt real-time optical imaging | 10.1016/j.bios.2019.04.052 |

| № | TITLE_SCIENTIFIC PUBLICATION | DIGITAL OBJECT IDENTIFIER (DOI) |
|---|---|---|
| 115 | Optical Slot Waveguide With Grating-Loaded Cladding of Silicon and Titanium Dioxide for Label-Free Bio-Sensing | 10.1109/JSEN.2019.2910278 |
| 116 | Lighting Up Biosensors: Now and the Decade To Come | 10.1021/acs.analchem.9b00793 |
| 117 | Cu/ITO-Coated Uncladded Fiber-Optic Biosensor Based on Surface Plasmon Resonance | 10.1109/LPT.2019.2908288 |
| 118 | Fiber Optic Sensor Based on Vernier Microwave Frequency Comb | 10.1109/JLT.2019.2917397 |
| 119 | An Optical Sensor Design Using Surface Modes of Low-Symmetric Photonic Crystals | 10.1109/JSEN.2019.2907508 |
| 120 | Electrical Impedance Characterization of Erythrocyte Response to Cyclic Hypoxia in Sickle Cell Disease | 10.1021/acssensors.9b00263 |
| 121 | Diaphragm-based optical fiber sensor for pulse wave monitoring and cardiovascular diseases diagnosis | 10.1002/jbio.201900084 |
| 122 | Simple and Low-Cost Plasmonic Fiber-Optic Probe as SERS and Biosensing Platform | 10.1002/adom.201900337 |
| 123 | Surface plasmon resonance photonic crystal fiber biosensor based on gold-graphene layers | 10.1016/j.yofte.2019.03.018 |
| 124 | Surface plasmon resonance refractive index sensor based on photonic crystal fiber covering nano-ring gold film | 10.1016/j.yofte.2019.03.018 |
| 125 | Performance Improvement of Refractometric Sensors Through Hybrid Plasmonic-Fano Resonances | 10.1109/JLT.2019.2906933 |
| 126 | Iontronic control of GaInAsP photonic crystal nanolaser | 10.1063/1.5098119 |
| 127 | Improved stability of gold nanoparticles on the optical fiber and their application to refractive index sensor based on localized surface plasmon resonance | 10.1016/j.optlastec.2019.02.002 |
| 128 | Guided Mode Resonance Sensors with Optimized Figure of Merit | 10.3390/nano9060837 |
| 129 | Modeling and Analysis of SOI Gratings-Based Opto-Fluidic Biosensor for Lab-on-a-Chip Applications | 10.3390/photonics6020071 |
| 130 | A Tunable High-Sensitivity Refractive Index of Analyte Biosensor Based on Metal-Nanoscale Covered Photonic Crystal Fiber With Surface Plasmon Resonance | 10.1109/JPHOT.2019.2915235 |
| 131 | Analysis of Dual-Core Photonic Crystal Fiber Based on Surface Plasmon Resonance Sensor with Segmented Silver Film | 10.1007/s11468-018-0846-8 |
| 132 | Highly sensitive refractive index sensor based on D-shaped PCF with gold-graphene layers on the polished surface | 10.1007/s00339-019-2731-5 |
| 133 | Planar optical waveguide for refractive index determining with high sensitivity and dual-band characteristic for Nano-sensor application | 10.1007/s11082-019-1905-x |
| 134 | A non-spectroscopic optical biosensor for the detection of pathogenic Salmonella Typhimurium based on a stem-loop DNA probe and retro-reflective signaling | 10.1186/s40580-019-0186-1 |
| 135 | Effect of plasmonic materials on photonic crystal fiber based surface plasmon resonance sensors | 10.1142/S0217984919501574 |

| № | TITLE_SCIENTIFIC PUBLICATION | DIGITAL OBJECT IDENTIFIER (DOI) |
|---|---|---|
| 136 | Detection of Interaction Between Peach-Gum Polysaccharides and Galectin-3 via Surface Plasmon Resonance Imaging | 10.3788/LOP56.09.2402 |
| 137 | Tapered Optical Fibre Sensors: Current Trends and Future Perspectives | 10.3390/s19102294 |
| 138 | Fiber optic plasmonic sensors: Providing sensitive biosensor platforms with minimal lab equipment | 10.1016/j.bios.2019.03.020 |
| 139 | Microscale and Nanoscale Electrophotonic Diagnostic Devices | 10.1101/cshperspect.a034249 |
| 140 | Optical enzymatic biosensor membrane for rapid in situ detection of organohalide in water samples | 10.1016/j.microc.2018.12.052 |
| 141 | U-shaped photonic quasi-crystal fiber sensor with high sensitivity based on surface plasmon resonance | 10.7567/1882-0786/ab13bc |
| 142 | Low-cost vertical taper for highly efficient light in-coupling in bimodal nanointerferometric waveguide biosensors | 10.1088/2515-7647/aafebb/pdf |
| 143 | High-Sensitive Fiber Anemometer Based on Surface Plasmon Resonance Effect in Photonic Crystal Fiber | 10.1109/JSEN.2019.2895265 |
| 144 | A novel dual-color total internal reflection fluorescence detecting platform using compact optical structure and silicon-based photodetector | 10.1016/j.talanta.2018.12.039 |
| 145 | Enhanced Sensitivity of Subwavelength Multibox Waveguide Microring Resonator Label-Free Biosensors | 10.1109/JSTQE.2018.2821842 |
| 146 | Carbon-Based, Ultraelastic, Hierarchically Coated Fiber Strain Sensors with Crack-Controllable Beads | 10.1021/acsami.9b03204 |
| 147 | Intensity-modulated nanoplasmonic interferometric sensor for MMP-9 detection | 10.1039/C8LC01391H |
| 148 | Design approach of solid-core photonic crystal fiber sensor with sensing ring for blood component detection | 10.1117/1.JNP.13.026011 |
| 149 | Construction of efficient bioelectrochemical devices: Improved electricity production from cyanobacteria (Leptolyngbia sp.) based on pi-conjugated conducting polymer/gold nanoparticle composite interfaces | 10.1002/bit.26885 |
| 150 | SPR Label-Free Biosensor with Oxide-Metal-Oxide-Coated D-Typed Optical Fiber: a Theoretical Study | 10.1007/s11468-018-0824-1 |
| 151 | Towards Portable Nanophotonic Sensors | 10.3390/s19071715 |
| 152 | Optical Biomarker-based Biosensors for Cancer/Infectious Disease Medical Diagnoses | 10.1097/PAI.0000000000000586 |
| 153 | Ultraflexible Nanowire Array for Label- and Distortion-Free Cellular Force Tracking | 10.1021/acs.nanolett.8b02568 |
| 154 | A theoretical study of a plasmonic sensor comprising a gold nano-disk array on gold film with a SiO2 spacer | 10.1088/1674-1056/28/4/044201 |
| 155 | An effective triple-band enhanced-infrared-absorption detection by honeycomb-shaped metamaterial-plasmonic absorber | 10.1016/j.sna.2019.02.006 |
| 156 | A Compact and Broadband Photonic Crystal Fiber Polarization Filter Based on a Plasmonic Resonant Thin Gold Film | 10.1109/JPHOT.2019.2899117 |
| 157 | Highly Flexible and Voltage Based Wavelength Tunable Biosensor | 10.1002/pssa.201800633 |
| 158 | Real-time monitoring of skin ethanol gas by a high-sensitivity gas phase biosensor (bio-sniffer) for the non-invasive evaluation of volatile compounds | 10.1016/j.bios.2018.09.070 |
| 159 | Waveguiding and SERS Simplified Raman Spectroscopy on Biological Samples | 10.3390/bios9010037 |

| № | TITLE_SCIENTIFIC PUBLICATION | DIGITAL OBJECT IDENTIFIER (DOI) |
|---|---|---|
| 160 | Effects of dispersion, absorption and interface fluctuation on the reflection spectra of porous silicon microcavity devices | 10.1007/s11801-019-8148-8 |
| 161 | Numerical modeling of graphene-coated fiber optic surface plasmon resonance biosensor for BRCA1 and BRCA2 genetic breast cancer detection | 10.1117/1.OE.58.3.037104 |
| 162 | Modeling of a fiber-optic surface plasmon resonance biosensor employing phosphorene for sensing applications | 10.1117/1.OE.58.3.037103 |
| 163 | Shape monitoring of morphing wing using micro optical sensors with different embedded depth | 10.1016/j.yofte.2018.12.025 |
| 164 | A dual-channel surface plasmon resonance biosensor based on a photonic crystal fiber for multianalyte sensing | 10.1007/s10825-019-01305-7 |
| 165 | Label-free biosensing using a microring resonator integrated with poly-(dimethylsiloxane) microfluidic channels | 10.1063/1.5074134 |
| 166 | Polymer-Templated Gold Nanoparticles on Optical Fibers for Enhanced-Sensitivity Localized Surface Plasmon Resonance Biosensors | 10.1021/acssensors.8b01372 |
| 167 | Core-clad phosphate glass fibers for biosensing | 10.1016/j.msec.2018.11.038 |
| 168 | High-Q Slot-Mode Photonic Crystal Nanobeam Cavity Biosensor With Optomechanically Enhanced Sensitivity | 10.1109/JSTQE.2018.2880592 |
| 169 | Design and Analysis of Surface-Plasmon-Resonance-Based Photonic Quasi-Crystal Fiber Biosensor for High-Refractive-Index Liquid Analytes | 10.1109/JSTQE.2018.2873481 |
| 170 | Optimized Bent Part Coupling SiON Racetrack Resonators for Biological Sensing | 10.1109/JSEN.2018.2879784 |
| 171 | Functionalized gold electroless-plated optical fiber gratings for reliable surface biosensing | 10.1016/j.snb.2018.09.115 |
| 172 | Photonic integrated circuits for Department of Defense-relevant chemical and biological sensing applications: state-of-the-art and future outlooks | 10.1117/1.OE.58.2.020901 |
| 173 | Analysis of highly sensitive biosensor for glucose based on a one-dimensional photonic crystal nanocavity | 10.1117/1.OE.58.2.027102 |
| 174 | The terahertz electromagnetically induced transparency-like metamaterials for sensitive biosensors in the detection of cancer cells | 10.1016/j.bios.2018.11.014 |
| 175 | Microfluidic chip coupled with optical biosensors for simultaneous detection of multiple analytes: A review | 10.1016/j.bios.2018.11.032 |
| 176 | Highly sensitive SPR PCF biosensors based on Ag/TiN and Ag/ZrN configurations | 10.1007/s11082-019-1764-5 |
| 177 | Propagation Controlled Photonic Crystal Fiber-Based Plasmonic Sensor via Scaled-Down Approach | 10.1109/JSEN.2018.2880161 |
| 178 | A sensitive biosensor based on optical bistability in a semiconductor quantum dot-DNA nanohybrid | 10.1088/1361-6463/aaec48 |
| 179 | Optical investigations of electrochemical processes using a long-period fiber grating functionalized by indium tin oxide | 10.1016/j.snb.2018.10.001 |
| 180 | Improvement of Resolution of Liquid Refractive Index Measurement Using Metallic Grating | 10.2528/PIERM19060304 |
| 181 | Cascaded-Microrings Biosensors Fabricated on a Polymer Platform | 10.3390/s19010181 |
| 182 | Development of Electrochemical Methods to Enzymatically Detect Lactate and Glucose Using Imaginary Impedance for Enhanced Management of Glycemic Compromised Patients | 10.1615/CritRevBiomedEng.2019026533 |
| 183 | Probing Lead Ion Contamination in Aqueous Solution Through Bio-Inspired Surface Modification of Gold Nanoparticles on D-Shaped Fiber | 10.1109/TNANO.2019.2929058 |
| 184 | A Novel Fiber-Based Symmetrical Long-Range Surface Plasmon Resonance Biosensor With High Quality Factor and Temperature Self-Reference | 10.1109/TNANO.2019.2947697 |

| № | TITLE_SCIENTIFIC PUBLICATION | DIGITAL OBJECT IDENTIFIER (DOI) |
|---|---|---|
| 185 | A simulation analysis for dimensioning of an amorphous silicon planar waveguide structure suitable to be used as a surface plasmon resonance biosensor | 10.1117/12.2526414 |
| 186 | 200-mm Silicon Photonics Technology Development | 10.1117/12.2537254 |
| 187 | Extrinsic Plasmonic Optical Fiber Sensors based on POFs and Bacterial Cellulose slab waveguides | 10.1117/12.2539335 |
| 188 | Graphene Oxide-functionalized Long Period Grating for biosensing applications | 10.1016/j.bios.2017.03.004 |
| 189 | 2D stiffness mapping for localizing osteoarthritic degenerated cartilage by using a fast indentation system based on fiber Bragg gratings | 10.1117/12.2539252 |
| 190 | Fiber-optics: a new route towards ultra-low detection limit label-free biosensing | 10.1117/12.2540008 |
| 191 | Advances in celiac disease testing | 10.1016/bs.acc.2019.03.001 |
| 192 | Plasmonic nanolasers based on graphene-insulator-metal platform | 10.23919/MOC46630.2019.8982904 |
| 193 | Janus particles: recent advances in the biomedical applications | 10.2147/IJN.S169030 |
| 194 | Microring resonator biosensing platform for sensitive detection of thrombin | 10.1117/12.2508782 |
| 195 | Breaking the trade-off between Q-factor and sensitivity for high-Q slot mode photonic crystal nanobeam cavity biosensors with optomechanical feedback | 10.1117/12.2509659 |
| 196 | Do you need a tunable laser for resonant cavity optical sensors? | 10.1117/12.2522352 |
| 197 | Laser biosensor based on micromechanical oscillator | 10.1117/12.2519607 |
| 198 | Influence of saline background on microstructured optical fibers optical properties | 10.1117/12.2523335 |
| 199 | Fully Integrated Liquid-Core Waveguide Fluorescence Lifetime Detection Microsystem for DNA Biosensing | 10.1109/ACCESS.2019.2934764 |
| 200 | Thin Film Coating of Copper Nanoparticles with DC Magnetron Sputtering via Physical Vapor Deposition | 10.1063/1.5118128 |
| 201 | Effect of anisotropy on the spectral characteristics of one-dimensional porous silicon photonic crystal microcavity for optical sensing applications | 10.1117/1.JNP.13.016012 |
| 202 | Wavelength dispersion phenomena observed for emitted optical radiation from a p plus nn plus silicon avalanche mode light-emitting device in a radio frequency bipolar-integrated circuitry | 10.1117/1.OE.58.1.017104 |
| 203 | A Hi-Bi Ultra-Sensitive Surface Plasmon Resonance Fiber Sensor | 10.1109/ACCESS.2019.2922663 |
| 204 | Nano-plate biosensor array using ultrafast heat transport through proteins | 10.1016/j.snb.2018.09.030 |
| 205 | Fiber-enhanced Raman spectroscopy as a tool for an early detection of Alzheimer's disease biomarkers | 10.1117/12.2516545 |
| 206 | . Mach-Zehnder interferometer based on core-offset splicing technique for sensing applications | 10.1117/12.2516545 |
| 207 | Real-time detection of BSA concentration based on a Mach-Zehnder interferometric biosensor | 10.1117/12.2522635 |
| 208 | Label-Free DNA Hybridization Monitoring through mFBG Biosensor with Self-Assembly Technique | 10.1117/12.2521352 |
| 209 | Integrated photonics for NASA applications | 10.1117/12.2509808 |
| 210 | Multi-functional plasmonic biosensor based on alcohol filled PCF | 10.1117/12.2509417 |

| № | TITLE_SCIENTIFIC PUBLICATION | DIGITAL OBJECT IDENTIFIER (DOI) |
|---|---|---|
| 211 | Bimetallic Surface Plasmon Resonance Photonic Crystal Fiber Biosensor Using Refractory Plasmonic Material | 10.1117/12.2509436 |
| 212 | Porous silicon sensors: From on-chip to mobile diagnostics | 10.1117/12.2508685 |
| 213 | Design and optimization of compact silicon photonic sensors | 10.1117/12.2502509 |
| 214 | Realizing Micro and Nano Optical Bio Sensors on Chip | 10.1117/12.2502491 |
| 215 | Continuous wave laser induced nonlinear optical response of nitrogen doped graphene oxide | 10.1117/12.2502491 |
| 216 | Integrated multichannel all-fiber optofluidic biosensing platform for sensitive and simultaneous detection of trace analytes | 10.1016/j.aca.2018.07.067 |
| 217 | Localized surface plasmon resonance based U-shaped optical fiber probe for the detection of Pb2+ in aqueous medium | 10.1016/j.snb.2018.08.086 |
| 218 | Polymeric biomaterials for biophotonic applications | 10.1016/j.snb.2018.08.065 |
| 219 | MXene-Based Nonlinear Optical Information Converter for All-Optical Modulator and Switcher | 10.1002/lpor.201800215 |
| 220 | Monolithically integrated InGaAsP multiple quantum well tunable laser diode for integrated optic surface plasmon resonance sensing | 10.1117/1.OE.57.12.120503 |
| 221 | Three-core photonic crystal fiber surface plasmon resonance sensor | 10.1016/j.yofte.2018.11.014 |
| 222 | High sensitivity photonic crystal fiber-based refractive index microbiosensor | 10.1016/j.yofte.2018.09.016 |
| 223 | High Sensitivity Photonic Crystal Fiber Refractive Index Sensor with Gold Coated Externally Based on Surface Plasmon Resonance | 10.3390/mi9120640 |
| 224 | Fano Resonance in Waveguide Coupled Surface Exciton Polaritons: Theory and Application in Biosensor | 10.3390/s18124437 |
| 225 | Multichannel Long-Range Surface Plasmon Waveguides for Parallel Biosensing | 10.1109/JLT.2018.2875953 |
| 226 | Design and numerical analysis of a novel dual-polarized refractive index sensor based on D-shaped photonic crystal fiber | 10.1088/1681-7575/aae757 |
| 227 | ? Photochemical deposition fabricated highly sensitive localized surface plasmon resonance based optical fiber sensor | 10.1016/j.optcom.2018.06.075 |
| 228 | Silicon plasmonic integrated interferometer sensor for lab on chip applications | 10.1016/j.optcom.2018.02.053 |
| 229 | Highly sensitive detection of exosomes by 3D plasmonic photonic crystal biosensor | 10.1039/C8NR07051B |
| 230 | Nano-electric field sensor based on Two Dimensional Photonic Crystal resonator | 10.1016/j.optmat.2018.09.016 |
| 231 | Sensitivity enhanced SPR immunosensor based on graphene oxide and SPA co-modified photonic crystal fiber | 10.1016/j.optlastec.2018.05.006 |
| 232 | Selective Uropathogenic E-coli Detection Using Crossed Surface-Relief Gratings | 10.3390/s18113634 |
| 233 | Robust Optimization of Nanoslit Array Sensor Based on Extraordinary Optical Transmission | 10.1109/JSEN.2018.2870300 |
| 234 | Surface Plasmon Resonance Sensor Based on Modified D-Shaped Photonic Crystal Fiber for Wider Range of Refractive Index Detection | 10.1109/JSEN.2018.2865514 |
| 235 | Directed self-assembly of a high-chi block copolymer for the fabrication of optical nanoresonators | 10.1039/C8NR05831H |
| 236 | Multilayered Nanoplasmonic Arrays for Self-Referenced Biosensing | 10.1021/acsami.8b12604 |

| № | TITLE_SCIENTIFIC PUBLICATION | DIGITAL OBJECT IDENTIFIER (DOI) |
|---|---|---|
| 237 | Theoretical evaluation of a fiber-optic SPR biosensor based on a gold layer treated with thiol acid | 10.1051/epjap/2018180059 |
| 238 | Plasmonic Biosensor in NIR with Chalcogenide Glass Material: On the Role of Probe Geometry, Wavelength, and 2D Material | 10.1007/s11220-018-0220-0 |
| 239 | Photonic crystal enhanced fluorescence immunoassay on diatom biosilica | 10.1002/jbio.201800009 |
| 240 | Silicon Photonic Biosensors Using Label-Free Detection | 10.3390/s18103519 |
| 241 | Research on dual-core photonic crystal fiber based on local surface plasmon resonance sensor with silver nanowires | 10.1117/1.JNP.12.046015 |
| 242 | Design of a surface plasmon resonance biosensor based on photonic crystal fiber with elliptical holes | 10.1007/s10043-018-0447-y |
| 243 | Highly sensitive nano-scale plasmonic biosensor utilizing Fano resonance metasurface in THz range: Numerical study | 10.1016/j.physe.2018.07.039 |
| 244 | Aluminum plasmonic waveguides co-integrated with Si3N4 photonics using CMOS processes | 10.1038/s41598-018-31736-4 |
| 245 | Optical Nitrite Biosensor based on Hemoglobin-Immobilized Polyacrylate Microspheres | 10.17576/jsm-2018-4709-10 |
| 246 | Improved Magneto-Optic Surface Plasmon Resonance Biosensors | 10.3390/photonics5030015 |
| 247 | Femtosecond-Pulsed Laser Written and Etched Fiber Bragg Gratings for Fiber-Optical Biosensing | 10.3390/s18092844 |
| 248 | Selective and sensitive Escherichia coli detection based on a T4 bacteriophage-immobilized multimode microfiber | 10.1002/jbio.201800012 |
| 249 | Highly sensitive PCF-SPR biosensor for hyperthermia temperature monitoring | 10.1007/s12596-018-0468-8 |
| 250 | Plastic optical fiber immunosensor for fast detection of sulfate-reducing bacteria | 10.1016/j.measurement.2018.04.088 |
| 251 | Label-free ferrule-top optical fiber micro-cantilever biosensor | 10.1016/j.sna.2018.07.014 |
| 252 | A wavelength-modulated localized surface plasmon resonance (LSPR) optical fiber sensor for sensitive detection of mercury(II) ion by gold nanoparticles-DNA conjugates | 10.1016/j.bios.2018.05.004 |
| 253 | Dual-core all-fiber integrated immunosensor for detection of protein antigens | 10.1016/j.bios.2018.05.008 |
| 254 | Autler-Townes splitting biosensing based on a nonuniform photonic crystal waveguide with feedback loop | 10.1364/AO.57.006976 |
| 255 | Buried Rib SiO2 Multimode Interference Waveguides for Optofluidic Multiplexing | 10.1109/LPT.2018.2858258 |
| 256 | A Highly Sensitive Metal-Insulator-Metal Ring Resonator-Based Nanophotonic Structure for Biosensing Applications | 10.1109/JSEN.2018.2849825 |
| 257 | Amplification-free, sequence-specific 16S rRNA detection at 1 aM | 10.1039/C8LC00452H |
| 258 | Optical bio-chemical sensors based on whispering gallery mode resonators | 10.1039/C8NR03709D |
| 259 | 3D-Printed Biosensor Arrays for Medical Diagnostics | 10.3390/mi9080394 |
| 260 | Photonic crystal microcavity as a highly sensitive platform for RI detection | 10.1016/j.cjph.2018.05.010 |
| 261 | A Highly Sensitive SPR Sensors Based on Two Parallel PCFs for Low Refractive Index Detection | 10.1109/JPHOT.2018.2856273 |
| 262 | A High-Performance Plasmonic Nanosensor Based on an Elliptical Nanorod in an MIM Configuration | 10.1109/JSEN.2018.2847760 |
| 263 | Gold-nanoparticle-based Fiber Optic Sensor for Sensing the Refractive Index of Environmental Solutions | — |
| 264 | Resonant Wavelength Shift Detection System Based on a Gradient Grating Period Guided-Mode Resonance | 10.1109/JPHOT.2018.2857505 |
| 265 | Optimization of porous silicon waveguide design for micro-ring resonator sensing applications | 10.1088/2040-8986/aad01b |

| № | TITLE_SCIENTIFIC PUBLICATION | DIGITAL OBJECT IDENTIFIER (DOI) |
|---|---|---|
| 266 | Twin Core Photonic Crystal Fiber Plasmonic Refractive Index Sensor | 10.1109/JSEN.2018.2841035 |
| 267 | Analysis and Improvement of a Dual-Core Photonic Crystal Fiber Sensor | 10.3390/s18072051 |
| 268 | Hand-held optical sensor using denatured antibody coated electro-active polymer for ultra-trace detection of copper in blood serum and environmental samples | 10.1016/j.bios.2018.03.040 |
| 269 | Photonic crystal resonances for sensing and imaging | 10.1088/2040-8986/aac75b |
| 270 | A Review of advancements (2007-2017) in plasmonics-based optical fiber sensors | 10.1016/j.yofte.2018.03.008 |
| 271 | Smallest microhouse in the world, assembled on the facet of an optical fiber by origami and welded in the mu Robotex nanofactory | 10.1116/1.5020128 |
| 272 | Theoretical Design of an Integrated Optical Sensor for a Standard Immunoassay | 10.1109/JSEN.2018.2824852 |
| 273 | Colorimetric determination of Cu2+ ions in water and milk by apo-tyrosinase disc | 10.1016/j.snb.2018.02.160 |
| 274 | An optofluidic metasurface for lateral flow-through detection of breast cancer biomarker | 10.1016/j.bios.2018.02.038 |
| 275 | Design of turn around point long period fiber grating sensor with Au-nanoparticle self monolayer | 10.1016/j.optlastec.2017.12.025 |
| 276 | Tunable Surface Plasmon Resonance Sensor Based on Photonic Crystal Fiber Filled with Gold Nanoshells | 10.1007/s11468-017-0570-9 |
| 277 | Ultrasensitive label-free optical microfiber coupler biosensor for detection of cardiac troponin I based on interference turning point effect | 10.1016/j.bios.2018.01.061 |
| 278 | A Smartphone-Based Red-Green Dual Color Fiber Optic Surface Plasmon Resonance Sensor | 10.1109/LPT.2017.2788560 |
| 279 | Graphene oxide functionalized long period fiber grating for highly sensitive hemoglobin detection | 10.1016/j.snb.2018.01.117 |
| 280 | Silicon Plasmonics On-Chip Mid-IR Gas Sensor | 10.1109/LPT.2018.2799208 |
| 281 | Fabrication of hexagonal star-shaped and ring-shaped patterns arrays by Mie resonance sphere-lens-lithography | 10.1016/j.apsusc.2017.12.200 |
| 282 | Optical fiber tips for biological applications: From light confinement, biosensing to bioparticles manipulation | 10.1016/j.bbagen.2018.02.008 |
| 283 | Microstructure and magneto-optical surface plasmon resonance of Co/Au multilayers | 10.1088/2399-6528/aac0e0 |
| 284 | Facile Fabrication of Novel Sensing System for Size Detection of Nanoparticles | 10.1109/TNANO.2018.2806306 |
| 285 | Robust immunosensing system based on biotin-streptavidin coupling for spatially localized femtogram mL(-1) level detection of interleukin-6 | 10.1016/j.bios.2017.11.023 |
| 286 | An optical fiber-based LSPR aptasensor for simple and rapid in-situ detection of ochratoxin A | 10.1016/j.bios.2017.11.062 |
| 287 | Alternative SNP detection platforms, HRM and biosensors, for varietal identification in Vitis vinifera L. using F3H and LDOX genes | 10.1038/s41598-018-24158-9 |
| 288 | On-Chip Oval-Shaped Nanocavity Photonic Crystal Waveguide Biosensor for Detection of Foodborne Pathogens | 10.1007/s11468-017-0529-x |
| 289 | High-Sensitivity Refractive Index Sensing Based on Fano Resonances in a Photonic Crystal Cavity-Coupled Microring Resonator | 10.1109/JPHOT.2018.2815622 |
| 290 | Mid-Infrared Sensor Based on a Suspended Microracetrack Resonator With Lateral Subwavelength-Grating Metamaterial Cladding | 10.1109/JPHOT.2018.2809662. |
| 291 | Giant enhancement of emission efficiency and light directivity by using hyperbolic metacavity on deep-ultraviolet AlGaN emitter | 10.1016/j.nanoen.2018.01.020 |

| № | TITLE_SCIENTIFIC PUBLICATION | DIGITAL OBJECT IDENTIFIER (DOI) |
|---|---|---|
| 292 | Highly sensitive photonic crystal fiber biosensor based on titanium nitride | 10.1007/s11082-018-1397-0 |
| 293 | A high-sensitivity photonic crystal fiber (PCF) based on the surface plasmon resonance (SPR) biosensor for detection of density alteration in non-physiological cells (DANCE) | 10.1016/j.opelre.2018.01.001 |
| 294 | Miniaturized Sample Preparation and Rapid Detection of Arsenite in Contaminated Soil Using a Smartphone | 10.3390/s18030777 |
| 295 | Integrated Optical Mach-Zehnder Interferometer Based on Organic-Inorganic Hybrids for Photonics-on-a-Chip Biosensing Applications | 10.3390/s18030840 |
| 296 | Geometrical comparison of photonic crystal fiber-based surface plasmon resonance sensors | 10.1117/1.OE.57.3.030801 |
| 297 | Photoinducible silane diazirine as an effective crosslinker in the construction of a chemiluminescent immunosensor targeting a model E. coli analyte | 10.1016/j.snb.2017.10.058 |
| 298 | Surface plasmon resonance fiber optic biosensor-based graphene and photonic crystal | 10.1142/S0217984918500720 |
| 299 | A label-free cardiac biomarker immunosensor based on phase-shifted microfiber Bragg grating | 10.1016/j.bios.2017.08.061 |
| 300 | A novel immunosensor based on excessively tilted fiber grating coated with gold nanospheres improves the detection limit of Newcastle disease virus | 10.1016/j.bios.2017.08.064 |
| 301 | Functionalized Long Period Grating-Plasmonic Fiber Sensor Applied to the Detection of Glyphosate in Water | 10.1109/JLT.2017.2739243 |
| 302 | Plasmonic Refractive Index Sensor Employing Niobium Nanofilm on Photonic Crystal Fiber | 10.1109/LPT.2017.2786475 |
| 303 | Colorimetric and Fiber Optic Sensing of Cysteine Using Green Synthesized Gold Nanoparticles | 10.1007/s11468-017-0517-1 |
| 304 | Surface Plasmon Resonance and Bending Loss-Based U-Shaped Plastic Optical Fiber Biosensors | 10.3390/s18020648 |
| 305 | Carbohydrate-protein interactions characterized by dual polarization hybrid plasmonic waveguide | 10.1515/ntrev-2017-0165 |
| 306 | Fibre Optic SPR Sensor Using Functionalized CNTs for the Detection of SMX: Comparison with Enzymatic Approach | 10.1007/s11468-017-0499-z |
| 307 | . Spectral Optical Readout of Rectangular-Miniature Hollow Glass Tubing for Refractive Index Sensing | 10.3390/s18020603 |
| 308 | A whole cell bio-optode based on immobilized nitrite-degrading microorganism on the acrylic microspheres for visual quantitation of nitrite ion | 10.1016/j.snb.2017.09.102 |
| 309 | Immobilized optical fiber microprobe for selective and high sensitive glucose detection | 10.1016/j.snb.2017.09.123 |
| 310 | Label free detection for DNA hybridization using surface plasmon photonic crystal fiber biosensor | 10.1007/s11082-017-1302-2 |
| 311 | Optical fiber sensor for dual sensing of H2O2 and DO based on CdSe/ZnS QDs and Ru(dpp)3(2+) embedded in EC matrix | 10.1016/j.snb.2017.08.071 |
| 312 | Highly Sensitive Terahertz Gas Sensor Based on Surface Plasmon Resonance With Graphene | 10.1109/JPHOT.2017.2778245 |
| 313 | Ultrahigh Sensitivity Plasmonic Refractive-Index Sensor for Aqueous Environment | 10.1109/LPT.2017.2779142 |
| 314 | Nonlinear Properties Of Water-soluble Ag2S And PbS Quantum Dots Under Picosecond Laser Pulses | 10.1088/1755-1315/186/4/012076 |
| 315 | Plasmonic Interferometer Array Biochip as a New Mobile Medical Device for Cancer Detection | 10.1109/JSTQE.2018.2865418 |
| 316 | Enhancement of plasma resonance in a Hi-Bi D-shaped photonic crystal fiber SPR sensor | 10.1117/12.2511830 |

| № | TITLE_SCIENTIFIC PUBLICATION | DIGITAL OBJECT IDENTIFIER (DOI) |
|---|---|---|
| 317 | Wavelength-Division-Multiplexed Fiber Bragg Grating sensor integrated with Surface Plasmon Resonance with the capability of multipoint, real-time remote sensing | 10.1143/JJAP.45.4588 |
| 318 | Resonant coupling from photonic crystal surfaces to plasmonic nanoantennas: Principles, detection instruments, and applications in digital resolution biosensing | 10.1117/12.2285828 |
| 319 | Lab-on-a-chip photonic biosensor for detection of antigens | 10.1117/12.2321128 |
| 320 | Ultracompact High-sensitivity Biosensing Based on Regenerable Laminated Bio-conjugation in Integrated Photonic Circuits | 10.1117/12.2321211 |
| 321 | Detection of various Thrombin concentrations using etched fiber Bragg gratings functionalized with DNA aptamer | 10.1117/12.2502457 |
| 322 | Fabrication and Performance of Ge-on-Si PIN Photodetectors | 10.1117/12.2501106 |
| 323 | High-resolution magnetic field biosensor based on optical resonators | 10.1117/12.2307587 |
| 324 | Highly Sensitive SPR based PCF for Biological Substance Sensing: Design and Analysis | 10.1117/12.2307061 |
| 325 | Integrated sensor biopsy device for real time tissue metabolism analysis | 10.1117/12.2290323 |
| 326 | Bragg grating in micro-scaled optical fiber for cardiac biomarker sensing | 10.1117/12.2284649 |
| 327 | Analysis of Highly Sensitive Surface Plasmon Photonic Crystal Fiber Biosensor | 10.1117/12.2290998 |
| 328 | Novel Ultra Low Power Optical Memory using Liquid Crystal | 10.1117/12.2306461 |
| 329 | Cartilage microindentation using cylindrical and spherical optical fiber indenters with integrated Bragg gratings as force sensors | 10.1117/12.2289493 |
| 330 | Fundamentals and biomedical applications of photonic crystals: An overview | 10.1117/12.2282019 |
| 331 | Design optimization of a single-mode microring resonator for label-free detection of biomarkers within a tunable spectral range of 2 nm | 10.1117/12.2318934 |
| 332 | Highly Sensitive Photonic Crystal Fiber Biosensor Based on Alternative Plasmonic Material | 10.1117/12.2306300 |
| 333 | Development towards Compact Nitrocellulose Interferometric Biochips for Dry Eye diagnosis based on MMP9, S100A6 and CST4 biomarkers using a Point-of-Care device | 10.1117/12.2288257 |
| 334 | D-Shaped photonic crystal fiber biosensor based on silver-graphene | 10.1016/j.ijleo.2018.04.119 |
| 335 | Resonant wavelength shift detection system based on a gradient grating period guided-mode resonance filter | 10.1117/12.2320414 |
| 336 | Sensitivity enhancement of surface plasmon resonance biosensor with graphene sandwiched between two metal films | 10.1117/12.2320025 |
| 337 | Intermixed ingaasp MQW Tunable laser diode suitable for probing surface plasmon resonance optical sensor | 10.1117/12.2503553 |
| 338 | Fish-bone subwavelength grating waveguide photonic integrated circuit sensor array | 10.1117/12.2305506 |
| 339 | Computational Study for Optimization of a Plasmon FET as a Molecular Biosensor | 10.1117/12.2288041 |
| 340 | Ultrasensitive colocalization detection based on plasmonic nanolithography with molecular-overlapped optical near-fields | 10.1117/12.2291449 |
| 341 | Silicon photonic resonator for label-free bio-sensing application | 10.1117/12.2300961 |
| 342 | Silicon-photonic interferometric biosensor using active phase demodulation | 10.1117/12.2287803 |

| № | TITLE_SCIENTIFIC PUBLICATION | DIGITAL OBJECT IDENTIFIER (DOI) |
|---|---|---|
| 343 | Investigation of ultrahigh sensitivity in GaInAsP nanolaser biosensor | 10.1117/12.2289408 |
| 344 | A highly sensitive Bezier polygonal hollow core photonic crystal fiber biosensor based on surface plasmon resonance | 10.1016/j.ijleo.2018.06.039 |
| 345 | Innovative 2D-nanomaterial integrated fiber optic sensors for biochemical applications | 10.1117/12.2307115 |
| 346 | Photonic crystal fiber-based plasmonic biosensor with external sensing approach | 10.1117/1.JNP.12.012503 |
| 347 | ▯ A highly sensitive surface plasmon resonance biosensor using photonic crystal fiber filled with gold nanowire encircled by silicon lining | 10.1016/j.ijleo.2017.10.157 |
| 348 | Square array photonic crystal fiber-based surface plasmon resonance refractive index sensor | 10.1142/S0217984917503523 |
| 349 | Surface plasmon resonance sensor based on photonic crystal fiber filled with gold-silica-gold multilayer nanoshells | 10.1016/j.optcom.2017.08.042 |
| 350 | Zinc(II) phthalocyanine fused in peripheral positions octa-substituted with alkyl linked carbazole: Synthesis, electropolymerization and its electro-optic and biosensor applications | 10.1016/j.bios.2017.06.028 |
| 351 | Sensitivity Analysis of Different Shapes of a Plastic Optical Fiber-Based Immunosensor for Escherichia coli: Simulation and Experimental Results | 10.3390/s17122944 |
| 352 | Two-Channel SPR Sensor Combined Application of Polymer- and Vitreous-Clad Optic Fibers | 10.3390/s17122862 |
| 353 | A Highly Sensitive Dual-Core Photonic Crystal Fiber Based on a Surface Plasmon Resonance Biosensor with Silver-Graphene Layer | 10.1007/s11468-016-0453-5 |
| 354 | Design, fabrication and characterisation of silica-titania thin film coated over coupled long period fibre gratings: Towards bio-sensing applications | 10.1016/j.snb.2017.06.139 |
| 355 | Split-ring resonator-based sensors on flexible substrates for glaucoma monitoring | 10.1016/j.sna.2017.10.054 |
| 356 | Graphene plasmonic nanogratings for biomolecular sensing in liquid | 10.1007/s00339-017-1380-9 |
| 357 | Fiber-Based Helical Channels Refractive Index Sensor Available for Microfluidic Chip | 10.1109/LPT.2017.2765314 |
| 358 | Indium Tin Oxide Coated Two-Mode Fiber for Enhanced SPR Sensor in Near-Infrared Region | 10.1109/JPHOT.2017.2757513 |
| 359 | Compact plasmonic optical biosensors based on nanostructured gradient index lenses integrated into microfluidic cells | 10.1039/C7NR04097K |
| 360 | Graphene Effect on the Improvement of the Response of Optical Fiber SPR Sensor | 10.1109/JSEN.2017.2758258 |
| 361 | Coagulation measurement from whole blood using vibrating optical fiber in a disposable cartridge | 10.1117/1.JBO.22.11.117001 |
| 362 | Development of a chemiluminescent DNA fibre optic genosensor to Hepatitis A Virus (HAV) | 10.1016/j.talanta.2017.06.036 |
| 363 | Localized surface plasmon resonance-based fiber-optic sensor for the detection of triacylglycerides using silver nanoparticles | 10.1117/1.JBO.22.10.107001 |
| 364 | Modulation of Visible and Near-Infrared Surface Plasmon Resonance of Au Nanoparticles Based on Highly Doped Graphene | 10.1007/s11468-016-0389-9 |
| 365 | Surface plasmon excitation using a Fourier-transform infrared spectrometer: Live cell and bacteria sensing | 10.1063/1.4997388 |

| № | TITLE_SCIENTIFIC PUBLICATION | DIGITAL OBJECT IDENTIFIER (DOI) |
|---|---|---|
| 366 | Repetitive Immunosensor with a Fiber-Optic Device and Antibody-Coated Magnetic Beads for Semi-Continuous Monitoring of Escherichia coli O157:H7 | 10.3390/s17092145 |
| 367 | Manufacturing and Spectral Features of Different Types of Long Period Fiber Gratings: Phase-Shifted, Turn-Around Point, Internally Tilted, and Pseudo-Random | 10.3390/fib5030029 |
| 368 | A Study on Refractive Index Sensors Based on Optical Micro-Ring Resonators | 10.1007/s13320-017-0418-0 |
| 369 | Fully Integrated Fluorescence Biosensors On-Chip Employing Multi-Functional Nanoplasmonic Optical Structures in CMOS | 10.1109/JSSC.2017.2712612 |
| 370 | Demonstration of low-cost and compact SPR optical transducer through edge light coupling | 10.1049/mnl.2017.0048 |
| 371 | An automated optofluidic biosensor platform combining interferometric sensors and injection moulded microfluidics | 10.1039/C7LC00524E |
| 372 | Optical Fiber Sensors for Label-Free DNA Detection | 10.1109/JLT.2016.2607024 |
| 373 | Graphene-MoS2 Hybrid Structure Enhanced Fiber Optic Surface Plasmon Resonance Sensor | 10.1007/s11468-016-0377-0 |
| 374 | Ultracompact On-Chip Multiplexed Sensor Array Based on Dense Integration of Flexible 1-D Photonic Crystal Nanobeam Cavity With Large Free Spectral Range and High Q-Factor | 10.1109/JPHOT.2017.2710136 |
| 375 | Design and numerical analysis of highly sensitive Au-MoS2-graphene based hybrid surface plasmon resonance biosensor | 10.1016/j.optcom.2017.03.035 |
| 376 | High sensitive and selective C-reactive protein detection by means of lossy mode resonance based optical fiber devices | 10.1016/j.bios.2016.09.020 |
| 377 | Design and optimization of silicon concentric dual-microring resonators for refractive index sensing | 10.1016/j.optcom.2016.05.052 |
| 378 | Plasmonic nanohole array biosensor for label-free and real-time analysis of live cell secretion | 10.1039/C7LC00277G |
| 379 | Substrate Oxide Layer Thickness Optimization for a Dual-Width Plasmonic Grating for Surface-Enhanced Raman Spectroscopy (SERS) Biosensor Applications | 10.3390/s17071530 |
| 380 | Microcantilever array instrument based on optical fiber and performance analysis | 10.1063/1.4994736 |
| 381 | A novel method of SPR based SnO2: GNP nano-hybrid decorated optical fiber platform for hexachlorobenzene sensing | 10.1016/j.snb.2017.02.123 |
| 382 | Towards a Uniform Metrological Assessment of Grating-Based Optical Fiber Sensors: From Refractometers to Biosensors. | 10.3390/bios7020023 |
| 383 | Optimal Design of Plasmonic Nanoslit Array Sensor Using Kriging Model | 10.1109/LPT.2017.2702583 |
| 384 | Simulation of the Sensing Performance of a Plasmonic Biosensor Based on Birefringent Solid-Core Microstructured Optical Fiber | 10.1007/s11468-016-0342-y |
| 385 | Towards a Uniform Metrological Assessment of Grating-Based Optical Fiber Sensors: From Refractometers to Biosensors | 10.3390/bios7020023 |
| 386 | Optimization of Surface Plasmon Resonance Biosensor with Ag/Au Multilayer Structure and Fiber-Optic Miniaturization | 10.1007/s11468-016-0312-4 |
| 387 | Plasmonic Interference-Based Refractive Index Sensor Designed with Spectral Analysis and Structure Optimization | 10.1007/s11468-016-0343-x |
| 388 | Theoretical analysis of microring resonator-based biosensor with high resolution and free of temperature influence | 10.1117/1.OE.56.6.067103 |
| 389 | Terahertz plasmon-induced transparency based on asymmetric dual-disk resonators coupled to a semiconductor InSb waveguide and its biosensor application | 10.1117/1.OE.56.6.067109 |
| 390 | Ultrasensitive and label -free detection of gamma-aminobutyric acid using fiber-optic interferometric sensors functionalized with size -selective molecular sieve arrays | ___ |

| № | TITLE_SCIENTIFIC PUBLICATION | DIGITAL OBJECT IDENTIFIER (DOI) |
|---|---|---|
| 391 | Polymer Integrated Waveguide Optical Biosensor by Using Spectral Splitting Effect | 10.1007/s13320-017-0395-3 |
| 392 | Sensing with ultra-short Fabry-Perot cavities written into optical micro-fibers | 10.1016/j.snb.2017.01.081 |
| 393 | Mid-infrared T-shaped photonic crystal waveguide for optical refractive index sensing | 10.1016/j.snb.2017.01.081 |
| 394 | Nanoscale patterning of gold-coated optical fibers for improved plasmonic sensing | 10.1088/1361-6528/aa6b53 |
| 395 | Bio-sniffer (gas-phase biosensor) with secondary alcohol dehydrogenase (S-ADH) for determination of isopropanol in exhaled air as a potential volatile biomarker | 10.1016/j.bios.2016.12.050 |
| 396 | High-sensitivity four-layer polymer fiber-optic evanescent wave sensor | 10.1016/j.bios.2017.01.019 |
| 397 | Recognition-mediated particle detection under microfluidic flow with waveguide-coupled 2D photonic crystals: towards integrated photonic virus detectors | 10.1039/C7LC00221A |
| 398 | A wide-range temperature immune refractive-index sensor using concatenated long-period-fiber-gratings | 10.1016/j.snb.2016.12.012 |
| 399 | Sensing metabolites for the monitoring of tissue engineered construct cellularity in perfusion bioreactors | 10.1016/j.bios.2016.09.094 |
| 400 | Determination of High-affinity Antibody-antigen Binding Kinetics Using Four Biosensor Platforms | 10.3791/55659 |
| 401 | Integration of a microfluidic polymerase chain reaction device and surface plasmon resonance fiber sensor into an inline all-in-one platform for pathogenic bacteria detection | 10.1016/j.snb.2016.10.137 |
| 402 | A highly sensitive and distinctly selective D-sorbitol biosensor using SDH enzyme entrapped Ta2O5 nanoflowers assembly coupled with fiber optic SPR | 10.1016/j.snb.2016.09.178 |
| 403 | Simultaneous measurement of refractive index and temperature using a SPR-based fiber optic sensor | /10.1016/j.snb.2016.09.164 |
| 404 | High Sensitivity Refractive Index Sensor Based on Multicoating Photonic Crystal Fiber With Surface Plasmon Resonance at Near-Infrared Wavelength | 10.1109/JPHOT.2017.2687121 |
| 405 | Thin cylindrical slot in an optical microdisk cavity for sensing biomaterials | 10.1007/s00339-016-0745-9 |
| 406 | Label-free protein sensing by employing blue phase liquid crystal | 10.1364/BOE.8.001712 |
| 407 | Cascaded ring resonator and Mach-Zehnder interferometer with a Sagnac loop for Vernier-effect refractive index sensing | 10.1016/j.snb.2016.08.095 |
| 408 | An array fluorescent biosensor based on planar waveguide for multi-analyte determination in water samples | 10.1016/j.snb.2016.08.118 |
| 409 | Recent Progress in Polymer Optical Fiber Light Sources and Fiber Bragg Gratings | 10.1109/JSTQE.2016.2616451 |
| 410 | Aptamer- and Fab'- Functionalized Microring Resonators for Aflatoxin M1 Detection | 10.1109/JSTQE.2016.2609100 |
| 411 | Detection of extremely low concentration waterborne pathogen using a multiplexing self-referencing SERS microfluidic biosensor | 10.1186/s13036-017-0051-x |
| 412 | Hybrid Graphene/Gold Plasmonic Fiber-Optic Biosensor | 10.1002/admt.201600185 |
| 413 | Functional magneto-plasmonic biosensors transducers: Modelling and nanoscale analysis | 10.1016/j.snb.2016.07.128 |
| 414 | One-port ring refractive index sensor with attached sub-ring | 10.1007/s10043-016-0283-x |
| 415 | Multi-functional optical sensor based on plasmonic photonic liquid crystal fibers | 10.1007/s11082-016-0849-7 |
| 416 | A novel nitrite biosensor based on the direct electrochemistry of horseradish peroxidase immobilized on porous Co3O4 nanosheets and reduced graphene oxide composite modified electrode | 10.1016/j.snb.2016.07.073 |

| № | TITLE_SCIENTIFIC PUBLICATION | DIGITAL OBJECT IDENTIFIER (DOI) |
|---|---|---|
| 417 | One-Dimensional Silicon Nitride Grating Refractive Index Sensor Suitable for Integration With CMOS Detectors | 10.1109/JPHOT.2016.2644962 |
| 418 | Competitive inhibition assay for the detection of progesterone in dairy milk using a fiber optic SPR biosensor | 10.1016/j.aca.2016.11.005 |
| 419 | High Sensitivity Refractive Index Sensor Based on Highly Overcoupled Tapered Fiber-Optic Couplers | 10.1109/JSEN.2016.2629281 |
| 420 | Localized Surface Plasmon Resonance (LSPR)-Coupled Fiber-Optic Nanoprobe for the Detection of Protein Biomarkers | 10.1007/978-1-4939-6848-0_1 |
| 421 | D-glucose sensor using photonic crystal fiber | 10.1016/j.ijleo.2017.08.039 |
| 422 | The Optical Properties of Quantum Dots Integrated in a Hollow Core Photon Crystal Fiber | 10.1117/12.2269378 |
| 423 | Measuring Spatial and Temporal Oxygen Flux Near Plant Tissues Using a Self-Referencing Optrode | 10.1007/978-1-4939-7292-0_23 |
| 424 | SPR Based Hybrid Electro-Optic Biosensor for beta-Lactam Antibiotics Determination in Water | 10.1117/12.2273318 |
| 425 | Precise sample preconcentration based on plasmon-assisted optical manipulation for a bead-based Raman biosensor | 10.1117/12.2295661 |
| 426 | Hopfield Neural Network and Optical Fiber Sensor as Intelligent Heart Rate Monitor | 10.1117/12.2283012 |
| 427 | The entangled photons generation in third order nonlinearity of spontaneous parametric down conversion by whispering gallery mode resonator | 10.1117/12.2285365 |
| 428 | Double-resonance long period fiber grating for detection of E. coli in trace concentration by choosing a proper bacteriophage | 10.1117/12.2265950 |
| 429 | Polyaniline deposition on tilted fiber Bragg grating for pH sensing | 10.1117/12.2267524 |
| 430 | Handheld highly selective plasmonic chem/biosensor using engineered binding proteins for extreme conformational changes | 10.1117/12.2273207 |
| 431 | High sensitive and selective Escherichia coli detection using immobilized optical fiber microprobe | 10.1117/12.2263456 |
| 432 | Trends in Fibre-Optic Uses for Personal Healthcare and Clinical Diagnostics | 10.1007/978-3-319-42625-9_6 |
| 433 | Sensitive Protein Detection and Quantification in Paper-Based Microfluidics for the Point of Care | 10.1016/bs.mie.2017.01.018 |
| 434 | Fabrication of Fiber Bragg Grating Coating with TiO2 Nanostructured Metal Oxide for Refractive Index Sensor | 10.1155/2017/2791282 |
| 435 | U-Bent Plastic Optical Fiber based Plasmonic Biosensor for Nucleic Acid Detection | 10.1117/12.2265597 |
| 436 | Evaluation of a Novel Label-Free Photonic-Crystal Biosensor Imaging System for the Detection of Prostate Cancer Cells | 10.1117/12.2249630 |
| 437 | High sensitive photonic crystal multiplexed biosensor array using H0 sandwiched cavities | 10.1051/epjconf/201713900003 |
| 438 | Monolithic integration of a plasmonic sensor with CMOS technology | 10.1117/12.2250504 |
| 439 | Modeling of a highly sensitive MoS2-Graphene hybrid based fiber optic SPR biosensor for sensing DNA hybridization | 10.1016/j.ijleo.2017.05.001 |
| 440 | Integration of Curved D-Type Optical Fiber Sensor with Microfluidic Chip | 10.3390/s17010063 |
| 441 | Fabricating 90 nm Resolution Structures in Sol-Gel Silica Optical Waveguides for Biosensor Applications | 10.1155/2017/4198485 |
| 442 | Disposable cartridge biosensor platform for portable diagnostics | 10.1117/12.2254729 |
| 443 | Silver-decorated silicon nanowires array as surface-enhanced Raman scattering (SERS) substrate | 10.1117/12.2251332 |
| 444 | Silicon nitride directional coupler interferometer for surface sensing | 10.1117/1.OE.56.1.017101 |
| 445 | Continuous Sensing Photonic Lab-on-a-Chip Platform Based on Cross-Linked Enzyme Crystals | 10.1021/acs.analchem.6b03793 |

| № | TITLE_SCIENTIFIC PUBLICATION | DIGITAL OBJECT IDENTIFIER (DOI) |
|---|---|---|
| 446 | Lattice plasmons in dielectric nanoparticle arrays arranged on metal film | 10.1088/2040-8978/18/12/125002 |
| 447 | Silicon-on-nitride slot waveguide: A promising platform as mid-IR trace gas sensor | 10.1016/j.snb.2016.06.060 |
| 448 | Full-Range Detection in Cascaded Microring Sensors Using Thermooptical Tuning | 10.1109/JLT.2016.2608979 |
| 449 | Optical weak measurement system with common path implementation for label-free biomolecule sensing | 10.1364/OL.41.005409 |
| 450 | Fluorescence based fiber optic and planar waveguide biosensors. A review | 10.1016/j.aca.2016.08.049 |
| 451 | A miniaturized oxygen sensor integrated on fiber surface based on evanescent-wave induced fluorescence quenching | 10.1016/j.jlumin.2016.08.005 |
| 452 | Surface plasmon resonance sensor based on photonic crystal fiber filled with core-shell Ag-Au nanocomposite materials | 10.1117/1.OE.55.11.117104 |
| 453 | Temperature Self-Compensated Optical Waveguide Biosensor Based on Cascade of Ring Resonator and Arrayed Waveguide Grating Spectrometer | 10.1109/JLT.2016.2603338 |
| 454 | Plasmonic spectrum on 1D and 2D periodic arrays of rod-shape metal nanoparticle pairs with different core patterns for biosensor and solar cell applications | 10.1088/2040-8978/18/11/115003 |
| 455 | Long-Range Surface Plasmon-Polariton Waveguide Biosensors for Disease Detection | opg.optica.org/jlt/abstract.cfm?URI=jlt-34-20-4673 |
| 456 | Biosensing With Asymmetric High Refractive Index Contrast Gratings | 10.1109/JSEN.2016.2599433 |
| 457 | Extraordinary Optical Transmission in a Hybrid Plasmonic Waveguide | 10.1109/JPHOT.2016.2613599 |
| 458 | Design of highly sensitive multichannel bimetallic photonic crystal fiber biosensor | 10.1117/1.JNP.10.046016 |
| 459 | Analysis and Design of Refractive Index Biosensors Based on Single Silicon Nanobeam Cavity | 10.1109/JPHOT.2016.2614501 |
| 460 | SPR-based plastic optical fibre biosensor for the detection of C-reactive protein in serum | 10.1002/jbio.201500315 |
| 461 | Controlled chemical modification of the internal surface of photonic crystal fibers for application as biosensitive elements | 10.1016/j.optmat.2016.07.046 |
| 462 | Recent Advances on Luminescent Enhancement-Based Porous Silicon Biosensors | 10.1007/s11095-016-1889-1 |
| 463 | Investigation of Silicon Carbide Based Optical Fiber Coupled Surface Plasmon Resonance Sensor | 10.1007/s12633-015-9394-4 |
| 464 | Localized Plasmon-Based Optical Fiber Sensing Platform for Operation in Infrared | 10.1109/LPT.2016.2573287 |
| 465 | Gold-reinforced silver nanoprisms on optical fiber tapers-A new base for high precision sensing | 10.1063/1.4953671 |
| 466 | Design of a lithium niobate-on-insulator-based optical microring resonator for biosensing applications | 10.1117/1.OE.55.8.087108 |
| 467 | Carbon dots for naked eye colorimetric ultrasensitive arsenic and glutathione detection | 10.1016/j.bios.2016.03.018 |
| 468 | Photonic crystal waveguide-based biosensor for detection of diseases | 10.1117/1.JNP.10.036011 |
| 469 | Active layer identification of photonic crystal waveguide biosensor chip for the detection of Escherichia coli | 10.1117/1.OE.55.7.077105 |
| 470 | Optical fiber resonance-based pH sensors using gold nanoparticles into polymeric layer-by-layer coatings | 10.1007/s00542-016-2857-8 |
| 471 | Surface plasmon resonance based fiber optic ethanol sensor using layers of silver/silicon/hydrogel entrapped with ADH/NAD | 10.1016/j.snb.2016.02.084 |
| 472 | Dispersion curve-based sensitivity engineering for enhanced surface plasmon resonance detection | 10.1016/j.optcom.2016.03.011 |
| 473 | Long period fiber grating nano-optrode for cancer biomarker detection | 10.1016/j.bios.2016.02.021 |

| № | TITLE_SCIENTIFIC PUBLICATION | DIGITAL OBJECT IDENTIFIER (DOI) |
|---|---|---|
| 474 | Probing the Localized Surface Plasmon Field of a Gold Nanoparticle-Based Fibre Optic Biosensor | 10.1007/s11468-015-0106-0 |
| 475 | Review of design principles of 2D photonic crystal microcavity biosensors in silicon and their applications | 10.1007/s11468-015-0106-0 |
| 476 | The Repetitive Detection of Toluene with Bioluminescence Bioreporter Pseudomonas putida TVA8 Encapsulated in Silica Hydrogel on an Optical Fiber | 10.3390/ma9060467 |
| 477 | SPR and SPR Imaging: Recent Trends in Developing Nanodevices for Detection and Real-Time Monitoring of Biomolecular Events | 10.3390/s16060870 |
| 478 | Homogeneous Biosensing Based on Magnetic Particle Labels | 10.3390/s16060828 |
| 479 | Self-Calibration Highly Sensitive Photonic Crystal Fiber Biosensor | 10.1109/JPHOT.2016.2563319 |
| 480 | SPR based fiber-optic sensor with enhanced electric field intensity and figure of merit using different single and bimetallic configurations | 10.1016/j.optcom.2016.01.014 |
| 481 | Refractive index change detection based on porous silicon microarray | 10.1007/s00340-016-6405-0 |
| 482 | Optically monitored drug delivery patch based on porous silicon and polymer microneedles | 10.1364/BOE.7.001645 |
| 483 | Evanescent Field Biosensor Using Polymer Slab Waveguide-Based Cartridges for the Optical Detection of Nanoparticles | 10.1109/JSTQE.2015.2481080 |
| 484 | Surface Plasmon Resonance Sensor for In Situ Detection of Xanthan Gum | 10.1109/JSTQE.2015.2477054 |
| 485 | Tracking micro-optical resonances for identifying and sensing novel procaspase-3 protein marker released from cell cultures in response to toxins | 10.1088/0957-4484/27/16/164001 |
| 486 | Designing a Biosensor Using a Photonic Quasi-Crystal Fiber | 10.1109/JSEN.2016.2514850 |
| 487 | Narrow band perfect absorber for maximum localized magnetic and electric field enhancement and sensing applications | 10.1038/srep24063 |
| 488 | Visualising apoptosis in live zebrafish using fluorescence lifetime imaging with optical projection tomography to map FRET biosensor activity in space and time | 10.1002/jbio.201500258 |
| 489 | Plasmonic optical sensor for determination of refractive index of human skin tissues | 10.1016/j.snb.2015.11.119 |
| 490 | Cascaded Ring-Resonators for Multi-Channel Optical Sensing With Reduced Temperature Sensitivity | 10.1109/LPT.2016.2514318 |
| 491 | Photonic biosensor based on photocorrosion of GaAs/AlGaAs quantum heterostructures for detection of Legionella pneumophila | 10.1116/1.4941983 |
| 492 | Progress in the research and development of photonic structure devices | 10.1088/2043-6262/7/1/015003 |
| 493 | Fiber optic and light scattering sensors: Complimentary approaches to rapid detection of Salmonella enterica in food samples | 10.1016/j.foodcont.2015.09.031 |
| 494 | Evanescent wave fluorescence biosensors: Advances of the last decade | 10.1016/j.bios.2015.07.040 |
| 495 | A Complete Optical Sensor System Based on a POF-SPR Platform and a Thermo-Stabilized Flow Cell for Biochemical Applications | 10.3390/s16020196 |
| 496 | Directional Coupler Biosensor Based on Thermooptic Detection Mechanism in Silica Waveguide | 10.1109/JPHOT.2016.2520819 |
| 497 | A SiON Microring Resonator-Based Platform for Biosensing at 850 nm | opg.optica.org/jlt/abstract.cfm?URI=jlt-34-3-969 |
| 498 | Multichannel photonic crystal fiber surface plasmon resonance based sensor | 10.1007/s11082-016-0414-4 |

| № | TITLE_SCIENTIFIC PUBLICATION | DIGITAL OBJECT IDENTIFIER (DOI) |
|---|---|---|
| 499 | Biosensors and bioelectronics on smartphone for portable biochemical detection | 10.1016/j.bios.2015.08.037 |
| 500 | Design optimization of long period waveguide grating devices for refractive index sensing using adaptive particle swarm optimization | 10.1016/j.optcom.2015.09.086 |
| 501 | Evanescent wave absorption based S-shaped fiber-optic biosensor for immunosensing applications | 10.1016/j.proeng.2016.11.161 |
| 502 | Optical biosensors | 10.1021/cr068105t |
| 503 | Bloch surface wave structures for high sensitivity detection and compact waveguiding | 10.1080/14686996.2016.1202082 |
| 504 | Modular Optofluidic Systems (MOPS) | 10.1117/12.2242997 |
| 505 | Immunogold-silver staining (IGSS) based U-bent fiberoptic sandwich biosensor | 10.1117/12.2242980 |
| 506 | Slotted-core photonic crystal fiber in gas sensing application | 10.1117/12.2247753 |
| 507 | Micro-capillary-Based Self-Referencing Surface Plasmon Resonance Fiber-Optic Biosensor | 10.1117/12.2245948 |
| 508 | Porous silicon-based two-dimensional photonic crystal for biochemical sensing applications | 10.1117/12.2246120 |
| 509 | Gold nano sphere based fiber optic LSPR probe for biosensing measurement | 10.1117/12.2245960 |
| 510 | Improved Optical Side Coupling Efficiency by Spiral Patterned Zinc Oxide Nanorod Coatings on Large Core Plastic Optical Fiber | 10.1117/12.2241640 |
| 511 | Investigation on optical properties of BSA protein on single-layer graphene using terahertz spectroscopy technology | 10.1117/12.2246258 |
| 512 | Nanohybrids Near-Field Optical Microscopy: From Image Shift to Biosensor Application | 10.1155/2016/4089260 |
| 513 | Optical waveguide materials, structures, and dispersion modulation | 10.1117/12.2249446 |
| 514 | Silicon chip integrated Photonic Sensors for Biological and Chemical Sensing | 10.1117/12.2223240 |
| 515 | Paper-based sensors and assays: a success of the engineering design and the convergence of knowledge areas | 10.1039/C6LC00737F |
| 516 | Compact surface plasmon resonance biosensor utilizing an injection-molded prism | 10.1117/12.2223682 |
| 517 | Engineering molecularly-active nanoplasmonic surfaces for DNA detection via colorimetry and Raman scattering | 10.1117/12.2209108 |
| 518 | Integrated Plasmonic Refractive Index Sensor based on Grating/Metal Film Resonant Structure | 10.1117/12.2218558 |
| 519 | Enhancement of effective quality-factor using asymmetric Mach-Zehnder interferometer with ring resonator for optical bio and chemical sensor | 10.1117/12.2212334 |
| 520 | Influence of the sensitivity of an optical resonator with a surface layer by its properties | 10.1117/12.2208542 |
| 521 | An embedded microretroreflector-based microfluidic immunoassay platform | 10.1039/C6LC00038J |
| 522 | Novel localized surface plasmon resonance based optical fiber sensor | 10.1117/12.2212652 |
| 523 | Improved sensitivity of the photonic crystal slab biosensors by using elliptical air holes | 10.1016/j.ijleo.2016.03.057 |
| 524 | Effects from detuning the resonant coupling between fiber gratings and localized surface plasmons | 10.1117/12.2234134 |
| 525 | Study on the effect of nanoparticle bimetallic coreshell Au-Ag for sensitivity enhancement of biosensor based on surface plasmon resonance | 10.1088/1742-6596/694/1/012075 |
| 526 | Recent advances in optical fiber devices for microfluidics integration | 10.1002/jbio.201500170 |

| № | TITLE_SCIENTIFIC PUBLICATION | DIGITAL OBJECT IDENTIFIER (DOI) |
|---|---|---|
| 527 | Surface plasmon resonance biosensor based on large size square-lattice photonic crystal fiber | 10.1080/09500340.2015.1102345 |
| 528 | High-sensitivity plasmonic sensor based on perfect absorber with metallic nanoring structures | 10.1080/09500340.2015.1066459 |
| 529 | Modulational instability of polarization of light in a periodically poled lithium niobate chip | 10.1117/1.OE.55.1.017105 |
| 530 | Cladding modes in photonic crystal fiber: characteristics and sensitivity to surrounding refractive index | 10.1117/1.OE.55.1.017106 |
| 531 | Integrated Photonic Nanofences: Combining Subwavelength Waveguides with an Enhanced Evanescent Field for Sensing Applications | 10.1021/acsnano.5b05864 |
| 532 | Micronanofabrication for optofluidic sensors | 10.3938/jkps.67.1992 |
| 533 | Highly sensitive color-indicating and quantitative biosensor based on cholesteric liquid crystal | 10.1364/BOE.6.005033 |
| 534 | Immobilization of cholesterol oxidase on magnetic fluorescent core-shell-structured nanoparticles | 10.1016/j.msec.2015.07.038 |
| 535 | Athermal optical waveguide microring biosensor with intensity interrogation | 10.1016/j.optcom.2015.07.036 |
| 536 | Nanoparticle-based lateral flow biosensors | 10.1016/j.bios.2015.05.050 |
| 537 | An acetone bio-sniffer (gas phase biosensor) enabling assessment of lipid metabolism from exhaled breath | 10.1016/j.bios.2015.04.023 |
| 538 | Ultrasensitive and Specific Measurement of Protease Activity Using Functionalized Photonic Crystals | 10.1021/acs.analchem.5b02529 |
| 539 | On the Performance of Graphene-Based D-Shaped Photonic Crystal Fibre Biosensor Using Surface Plasmon Resonance | 10.1007/s11468-015-9912-7 |
| 540 | Graphene Enhances the Sensitivity of Fiber-Optic Surface Plasmon Resonance Biosensor | 10.1109/JSEN.2015.2442276 |
| 541 | High-Sensitivity Biochemical Sensor Based on Cylindrical Nano-Metal Particles Array | opg.optica.org/jlt/abstract.cfm?URI=jlt-33-17-3635 |
| 542 | Sensitivity analysis for improving nanomechanical photonic transducers biosensors | 10.1088/0022-3727/48/33/335401 |
| 543 | A low cost surface plasmon resonance biosensor using a laser line generator | 10.1016/j.optcom.2015.03.035 |
| 544 | Beta-Sheet-Forming, Self-Assembled Peptide Nanomaterials towards Optical, Energy, and Healthcare Applications | 10.1002/smll.201500169 |
| 545 | Selective detection of bacteria in urine with a long-range surface plasmon waveguide biosensor | 10.1364/BOE.6.002908 |
| 546 | Nanoporous silicon microcavity based optical sensor to detect adulteration of petrol by organic solvents | 10.1007/s11082-014-0107-9 |
| 547 | Absorption enhancement and sensing properties of Ag diamond nanoantenna arrays | 10.1088/1674-1056/24/7/074206 |
| 548 | Reusable Bacteriophage Adhesin-Coated Long-Period Grating Sensor for Bacterial Lipopolysaccharide Recognition | opg.optica.org/jlt/abstract.cfm?URI=jlt-33-12-2518 |
| 549 | Polarimetric Plasmonic Sensing with Bowtie Nanoantenna Arrays | 10.1007/s11468-014-9856-3 |
| 550 | Experimental Validation of the Sensitivity of Waveguide Grating Based Refractometric (Bio) sensors | 10.3390/bios5020187 |
| 551 | An Optical Biosensor from Green Fluorescent Escherichia coli for the Evaluation of Single and Combined Heavy Metal Toxicities | 10.3390/s150612668 |
| 552 | Recognition of bacterial lipopolysaccharide using bacteriophage-adhesin-coated long-period gratings | 10.1016/j.bios.2014.07.027 |
| 553 | Picosecond optically reconfigurable filters exploiting full free spectral range tuning of single ring and Vernier effect resonators | 10.1364/OE.23.012468 |
| 554 | Review of plasmonic fiber optic biochemical sensors: improving the limit of detection | 10.1007/s00216-014-8411-6 |
| 555 | Angularly resolved ellipsometric optical biosensing by means of Bloch surface waves | 10.1007/s00216-015-8591-8 |

| № | TITLE_SCIENTIFIC PUBLICATION | DIGITAL OBJECT IDENTIFIER (DOI) |
|---|---|---|
| 556 | Optical Nano Antennas: State of the Art, Scope and Challenges as a Biosensor Along with Human Exposure to Nano-Toxicology | 10.3390/s150408787 |
| 557 | Tunable Plasmonic Sensor With Metal-Liquid Crystal-Metal Structure | 10.1109/JPHOT.2015.2411214 |
| 558 | Optical biosensor based on a silicon nanowire ridge waveguide for lab on chip applications | 10.1088/2040-8978/17/4/045802 |
| 559 | Design of grating couplers and MMI couplers on the TriPleX platform enabling ultra-compact photonic-based biosensors | 10.1016/j.snb.2014.11.098 |
| 560 | Fabrication of long-range surface plasmon-polariton Bragg gratings with microfluidic channels in Cytop claddings | 10.1016/j.mee.2015.03.001 |
| 561 | Optical surface plasmon resonance biosensors in molecular fishing | 10.18097/PBMC20156102231 |
| 562 | Numeric Tuning of Surface Plasmon Enhanced Spontaneous Emission Inducted by Nano-Metallic Particle Systems Embedded in GaN-Based LED | 10.1109/JDT.2014.2385592 |
| 563 | Gold Elliptic Nanocavity Array Biosensor With High Refractive Index Sensitivity Based on Two-Photon Nanolithography | 10.1109/JPHOT.2014.2366167 |
| 564 | Efficient optical biochemical sensor with slotted Bragg-grating-based Fabry-Perot resonator structure in silicon-on-insulator platform | 10.1007/s11082-014-9908-0 |
| 565 | On-line biosensor for the detection of putative toxicity in water contaminants | 10.1016/j.talanta.2014.09.032 |
| 566 | Magneto-plasmonic biosensor with enhanced analytical response and stability | 10.1016/j.bios.2014.08.004 |
| 567 | Detection of unamplified genomic DNA by a PNA-based microstructured optical fiber (MOF) Bragg-grating optofluidic system | 10.1016/j.bios.2014.07.047 |
| 568 | МЭМС ДЛЯ ДИАГНОСТИКИ ВОЗБУДИТЕЛЕЙ ЗАБОЛЕВАНИЙ | ISNN:1813-8586 |
| 569 | A Fluorometric Biochemical Gas Sensor (Biosniffer) for Acetaldehyde Vapor Based on Catalytic Reaction of Aldehyde Dehydrogenase | ISSN: 0914-4935 |
| 570 | A tunable submicro-optofluidic polymer filter based on guided-mode resonance | 10.1039/C4NR07233B |
| 571 | 3D plasmonic nanoantennas integrated with MEA biosensors | 10.1039/C4NR05578K |
| 572 | Detecting single DNA molecule interactions with optical microcavities | 10.1117/12.2190649 |
| 573 | Design and optimization of diamond-shaped biosensor using photonic crystal nano-ring resonator | 10.1016/j.ijleo.2015.06.037 |
| 574 | Electromagnetic resonances and their tunability in planar metamolecules isomer | 10.1016/j.ijleo.2015.07.033 |
| 575 | Surface passivation of a photonic crystal band-edge laser by atomic layer deposition of SiO2 and its application for biosensing | 10.1039/C4NR07552H |
| 576 | Lab-on-Fiber biosensing for cancer biomarker detection | 10.1117/12.2194943 |
| 577 | Trends in Biosensors for HPV: Identification and Diagnosis | 10.1155/2015/913640 |
| 578 | Fiber-optic triggered release of liposome in vivo: implication of personalized chemotherapy | 10.2147/IJN.S85915 |
| 579 | Optical biosensor technologies for molecular diagnostics at the point-of-care | 10.1117/12.2185642 |
| 580 | Engineering optical near-fields for highly efficient surface-enhanced nanoplasmonics | 10.1117/12.2189754 |
| 581 | Enhancing enzymatic efficiency by attachment to semiconductor nanoparticles for biosensor applications | 10.1117/12.2180645 |
| 582 | Modeling and performance analysis of chalcogenide prism based plasm onic biosensor comprising of gold nanoparticle film | 10.1117/12.2178849 |

| № | TITLE_SCIENTIFIC PUBLICATION | DIGITAL OBJECT IDENTIFIER (DOI) |
|---|---|---|
| 583 | Efficient nanoplasmonic antennas for fabricating single protein molecule detector | 10.1117/12.2182874 |
| 584 | Optical fiber pH sensor based on gold nanoparticles into polymeric coatings | 10.1117/12.2179970 |
| 585 | Cancer-cells on a chip for label-free optic detection of secreted molecules | 10.1117/12.2179883 |
| 586 | Photoluminescence characterization of ZnO nanowires functionalization | 10.1117/12.2178593 |
| 587 | Influence of the permeable layer number of porous silicon microcavity on reflection spectrum | 10.1016/j.ijleo.2015.01.026 |
| 588 | A reusable biosensor chip for SERS-fluorescence dual mode immunoassay | 10.1117/12.2180410 |
| 589 | A Multiplexed Label Free Plasmonic Nano-Device for Near Infrared Applications | 10.1063/1.4908581 |
| 590 | Low-cost high performance readout system for fiber-optic biosensors | 10.1117/12.2080209 |
| 591 | Theoretical study on sensing performance of hydrogen annealed silicon waveguides | 10.1080/09500340.2014.975846 |
| 592 | Fiber-optic sensors for high throughput screening of pathogens | 10.1016/B978-0-85709-801-6.00010-1 |
| 593 | Analyzing the biosensor signal in flows: Studies with glucose optrodes | 10.1016/j.talanta.2014.07.061 |
