# Supplementary material for: A review of Optical Point-of-Care devices to Estimate the Technology Transfer of These Cutting-Edge Technologies
Source: Biosensors (Basel). 2022 Nov 29;12(12):1091. doi: 10.3390/bios12121091 (PMC9776401; doi:10.3390/bios12121091)
Supplement: Supplementary file 1 [file biosensors-12-01091-s001.zip › Table S3 Suppl_File.pdf]

APPENDIX C _LIST OF INCLUDED ARTICLES		
**Nº**	**TITLE_SCIENTIFIC PUBLICATION**	**DIGITAL OBJECT IDENTIFIER (DOI)**
1	Optical fiber bio-sensor for phospholipase using liquid crystal	10.1016/j.bios.2020.112547
2	The potential of terahertz sensing for cancer diagnosis	10.1016/j.heliyon.2020.e05623
3	Polymer-Coated Fiber Optic Sensor as a Process Analytical Tool for Biopharmaceutical Impurity Detection	10.1109/TIM.2020.2981982
4	D-type photonic crystal fiber sensor based on metal nanowire array	10.1016/j.ijleo.2020.165010
5	Highly Sensitive D-Shaped Optical Fiber Surface Plasmon Resonance Refractive Index Sensor Based on Ag-alpha-Fe2O3 Grating	10.1109/JSEN.2020.2992854
6	3-D Printed Instrumentation for Point-of-Use Leaky Waveguide Biochemical Sensor	10.1109/TIM.2020.2969036
7	A Plasmonic Nano-Biosensor Based on Two Consecutive Disk Resonators and Unidirectional Reflectionless Propagation Effect	10.1109/JSEN.2020.2987319
8	Urea detection using bio-synthesized gold nanoparticles: an SPR/LSPR based sensing approach realized on optical fiber	10.1007/s11082-020-02405-6
9	A D-Shaped Fiber Long-Range Surface Plasmon Resonance Sensor With High Q-Factor and Temperature Self-Compensation	10.1109/TIM.2019.2920187
10	Microcapillary-Based Integrated LSPR Device for Refractive Index Detection and Biosensing	10.1109/JLT.2020.2969016
11	Simultaneous optical and electrochemical label-free biosensing with ITO-coated lossy-mode resonance sensor	10.1016/j.bios.2020.112050
12	Au nanoparticles as label-free competitive reporters for sensitivity enhanced fiber-optic SPR heparin sensor	10.1016/j.bios.2020.112039
13	Development of Uric Acid Biosensor Using Gold Nanoparticles and Graphene Oxide Functionalized Micro-Ball Fiber Sensor Probe	10.1109/TNB.2019.2958891
14	Measurement of MIPs Responses Deposited on Two SPR-POF Sensors Realized by Different Photoresist Buffer Layers	10.1109/TIM.2020.2967864
15	Fiber optic nanogold-linked immunosorbent assay for rapid detection of procalcitonin at femtomolar concentration level	10.1016/j.bios.2019.111871
16	Performance analysis of graphene-based surface plasmon resonance biosensor for blood glucose and gas detection	10.1007/s00339-020-3328-8
17	Lossy mode resonance sensors based on nanocoated multimode-coreless-multimode fibre	10.1016/j.snb.2019.126955
18	S-shaped long period fiber grating glucose concentration biosensor based on immobilized glucose oxidase	10.1016/j.ijleo.2019.163960

Nº	TITLE_SCIENTIFIC PUBLICATION	DIGITAL OBJECT IDENTIFIER (DOI)
19	Reflectance aptasensor based on metal salphen label for rapid and facile determination of insulin	10.1016/j.talanta.2019.120321
20	Multiplexed Remote SPR Detection of Biological Interactions through Optical Fiber Bundles	10.3390/s20020511
21	MoSe2-Au Based Sensitivity Enhanced Optical Fiber Surface Plasmon Resonance Biosensor for Detection of Goat-Anti-Rabbit IgG	10.1109/ACCESS.2019.2961751
22	A Compact Biosensor for Binding Kinetics Analysis of Protein-Protein Interaction	10.1109/JSEN.2019.2938655
23	Functionalized etched tilted fiber Bragg grating aptasensor for label-free protein detection	10.1016/j.bios.2019.111765
24	A novel optical fiber glucose biosensor based on carbon quantum dots-glucose oxidase/cellulose acetate complex sensitive film	10.1016/j.bios.2019.111760
25	Highly sensitive and selective localized surface plasmon resonance biosensor for detecting glutamate realized on optical fiber substrate using gold nanoparticles	10.1016/j.photonics.2019.100730
26	Plasmonic Diffraction Field Pattern Imaging Could Resolve Ultrasensitive Bioinformation	10.1021/acsphotonics.9b01076
27	A highly selective LSPR biosensor for the detection of taurine realized on optical fiber substrate and gold nanoparticles	10.1016/j.yofte.2019.101962
28	Highly sensitive label-free in vitro detection of aflatoxin B1 in an aptamer assay using optical planar waveguide operating as a polarization interferometer	10.1007/s00216-019-02033-4
29	Sensitivity enhanced D-type large-core fiber SPR sensor based on Gold nanoparticle/Au film co-modification	10.1016/j.optcom.2019.06.026
30	Fiber optic sensor based on ZnO nanowires decorated by Au nanoparticles for improved plasmonic biosensor	10.1038/s41598-019-52056-1
31	Smart-phone, paper-based fluorescent sensor for ultra-low inorganic phosphate detection in environmental samples	10.1038/s41378-019-0096-8
32	Full integration of photonic nanoimmunosensors in portable platforms for on-line monitoring of ocean pollutants	10.1016/j.snb.2019.126758
33	Non-enzymatic D-glucose plasmonic optical fiber grating biosensor	10.1016/j.bios.2019.111506

№	TITLE_SCIENTIFIC PUBLICATION	DIGITAL OBJECT IDENTIFIER (DOI)
34	High-sensitivity ultra-quality factor and remarkable compact blood components biomedical sensor based on nanocavity coupled photonic crystal	10.1016/j.rinp.2019.102478
35	Computational Study of Photonic Crystal Resonator for Biosensor Application	10.1515/freq-2019-0025
36	A New Device Based on Interferometric Optical Detection Method for Label-Free Screening of C-Reactive Protein	10.1109/TIM.2018.2876073
37	LSPR-Based Cholesterol Biosensor Using Hollow Core Fiber Structure	10.1109/JSEN.2019.2916818
38	Highly Sensitive SPR Biosensor Based on Graphene Oxide and Staphylococcal Protein A Co-Modified TFBG for Human IgG Detection	10.1109/TIM.2018.2875961
39	Use of an electro-optical sensor and phage antibodies for immunodetection of Herbaspirillum	10.1016/j.talanta.2019.04.086
40	Fano resonances based on plasmonic square resonator with high figure of merits and its application in glucose concentrations sensing	10.1007/s11082-019-2007-5
41	Preparation of Graphene/ITO Nanorod Metamaterial/U-Bent-Annealing Fiber Sensor and DNA Biomolecule Detection	10.3390/nano9081154
42	Fabrication of a Bare Optical Fiber-Based Biosensor	10.3390/mi10080522
43	Hydrogel optical fibers for continuous glucose monitoring	10.1016/j.bios.2019.05.002
44	Black phosphorus based fiber optic biosensor for ultrasensitive cancer diagnosis	10.1016/j.bios.2019.04.044
45	Development of an optical biosensor for the detection of Trypanosoma evansi and Plasmodium berghei	. 10.1016/j.saa.2019.04.008
46	Adaptive and sensitive fibre-optic fluorimetric transducer for air- and water-borne	10.1016/j.talanta.2019.02.055
47	An ultra-sensitive aptasensor on optical fibre for the direct detection of bisphenol A	10.1016/j.bios.2019.02.043
48	LSPR based optical fiber sensor with chitosan capped gold nanoparticles on BSA for trace detection of Hg (II) in water, soil and food samples	10.1016/j.bios.2019.03.046
49	Acoustofluidic Micromixing Enabled Hybrid Integrated Colorimetric Sensing, for Rapid Point-of-Care Measurement of Salivary Potassium	10.3390/bios9020073

Nº	TITLE_SCIENTIFIC PUBLICATION	DIGITAL OBJECT IDENTIFIER (DOI)
50	Gold Nanoparticle-Functionalized Surface Plasmon Resonance Optical Fiber Biosensor: In Situ Detection of Thrombin With 1 n.M Detection Limit	10.1109/JLT.2018.2822827
51	Label-Free Detection of DNA Hybridization Utilizing Dual S-Tapered Thin-Core Fiber Interferometer	10.1109/JLT.2018.2864798
52	In-Situ Detection of Small Biomolecule Interactions Using a Plasmonic Tilted Fiber Grating Sensor	10.1109/JLT.2018.2870337
53	Tricore photonic crystal fibre based refractive index sensor for glucose detection	10.1049/iet-opt.2018.5079
54	Two-dimensional transition metal dichalcogenides assisted biofunctionalized optical fiber SPR biosensor for efficient and rapid detection of bovine serum albumin	10.1038/s41598-019-43531-w
55	Waveguide-based chemo- and biosensors: complex emulsions for the detection of caffeine and proteins	10.1039/c9lc00070d
56	Investigation of a low cost tapered plastic fiber optic biosensor based on manipulation of colloidal gold nanoparticles	10.1016/j.optcom.2018.12.088
57	Microfluidics Integrated Lithography-Free Nanophotonic Biosensor for the Detection of Small Molecules	10.1002/adom.201801313
58	A Fiber Optic Biosensor Based on Hydrogel-Immobilized Enzyme Complex for Continuous Determination of Cholesterol and Glucose	10.1007/s12010-018-2897-x
59	Plasmonic biosensors for bacterial endotoxin detection on biomimetic C-18 supported fiber optic probes	10.1016/j.bios.2018.12.045
60	Separation and Detection of Escherichia coli and Saccharomyces cerevisiae Using a Microfluidic Device Integrated with an Optical Fibre	10.3390/bios9010040
61	Disk-based one-dimensional photonic crystal slabs for label-free immunosensing	10.1016/j.bios.2018.11.005
62	Rapid detection of Escherichia coli using fiber optic surface plasmon resonance immunosensor based on biofunctionalized Molybdenum disulfide (MoS2) nanosheets	10.1016/j.bios.2018.11.006
63	Development of dual-color total internal reflection fluorescence biosensor for simultaneous quantitation of two small molecules and their affinity constants with antibodies	10.1016/j.bios.2018.12.010

Nº	TITLE_SCIENTIFIC PUBLICATION	DIGITAL OBJECT IDENTIFIER (DOI)
64	? Hybrid Metasurface-Based Mid-Infrared Biosensor for Simultaneous Quantification and Identification of Monolayer Protein	
65	Nucleic acid functionalized fiber optic probes for sensing in evanescent wave: optimization and application	10.1039/c8ra10125f
66	Development of novel portable and reusable fiber optical chemiluminescent biosensor and its application for sensitive detection of microcystin-LR	10.1016/j.bios.2018.08.062
67	Surface plasmon resonance biosensor based on graphene oxide/silver coated polymer cladding silica fiber	10.1016/j.snb.2018.08.065
68	Etched Fiber Bragg Grating Biosensor Functionalized with Aptamers for Detection of Thrombin	10.3390/s18124298
69	Enhanced sensitivity of hemoglobin sensor using dual-core photonic crystal fiber	10.1007/s11082-018-1710-y
70	Ultrasensitive biosensor based on long period grating coated with polycarbonate-graphene oxide multilayer	10.1016/j.snb.2018.08.002
71	Rapid and real-time diagnosis of hypoalbuminemia using an extraordinary optical transmission biosensor	10.1016/j.snb.2018.07.119
72	? Real-time detection of prostate-specific antigens using a highly reliable fiber-optic localized surface plasmon resonance sensor combined with micro fluidic channel	10.1016/j.snb.2018.07.007
73	In-situ ultrasensitive label-free DNA hybridization detection using optical fiber specklegram	10.1016/j.snb.2018.05.099
74	A Fluidic Biosensor Based on a Phase-Sensitive Low-Coherence Spectral-Domain Interferometer	10.3390/s18113757
75	Rapid detection of cocaine using aptamer-based biosensor on an evanescent wave fibre platform	10.1098/rsos.180821
76	Optical Micro/Nanofiber-Based Localized Surface Plasmon Resonance Biosensors: Fiber Diameter Dependence	10.3390/s18103295
77	A Plasmonic Fiber Based Glucometer and Its Temperature Dependence	10.3390/mi9100506
78	Optical conductivity-based ultrasensitive mid-infrared biosensing on a hybrid metasurface	10.1038/s41377-018-0066-1
79	Thin-core fiber-optic biosensor for DNA hybridization detection	10.1007/s11801-018-8054-5

№	TITLE_SCIENTIFIC PUBLICATION	DIGITAL OBJECT IDENTIFIER (DOI)
80	LED-Based Portable Optical Biosensor for Measurement of Serum Urea Levels Using Urease Immobilized Agarose-Guar Gum Composite Film	10.1109/JSEN.2018.2850909
81	Highly sensitive colorimetric paper sensor for methyl isothiocyanate (MITC): Using its toxicological reaction	10.1016/j.snb.2018.01.086
82	A simple approach for fabrication of optical affinity-based bioanalytical microsystem on polymeric PEN foils	10.1016/j.colsurfb.2018.01.048
83	Hydrogen peroxide and glucose concentration measurement using optical fiber grating sensors with corrodible plasmonic nanocoatings	10.1364/BOE.9.001735
84	Graphene/Au-Enhanced Plastic Clad Silica Fiber Optic Surface Plasmon Resonance Sensor	10.1007/s11468-017-0534-0
85	Glucose Sensor Using U-Shaped Optical Fiber Probe with Gold Nanoparticles and Glucose Oxidase	10.3390/s18041217
86	Urinary p-cresol diagnosis using nanocomposite of ZnO/MoS2 and molecular imprinted polymer on optical fiber based lossy mode resonance sensor	10.1016/j.bios.2017.10.029
87	Uric acid sensing using tapered silica optical fiber coated with zinc oxide nanorods	10.1002/mop.31032
88	Phase-sensitive plasmonic biosensor using a portable and large field-of-view interferometric microarray imager	10.1038/lsa.2017.152
89	LSPR- and SPR-Based Fiber-Optic Cholesterol Sensor Using Immobilization of Cholesterol Oxidase Over Silver Nanoparticles Coated Graphene Oxide Nanosheets	10.1109/JSEN.2017.2779519
90	DNA origami nanorobot fiber optic genosensor to TMV	10.1016/j.bios.2017.07.051
91	Application of Fiber Optic Biosensor in Detection of Sports Analeptic	10.7546/ijba.2018.22.4.301-314
92	A label-free fiber optic SPR biosensor for specific detection of C-reactive protein	10.1038/s41598-017-17276-3
93	A Low-Cost and Portable Dual-Channel Fiber Optic Surface Plasmon Resonance System.	10.3390/s17122797
94	Square-microfiber-integrated biosensor for label-free DNA hybridization detection	10.1016/j.snb.2017.07.168
95	U-bent fiber optic SPR sensor based on graphene/AgNPs	10.1016/j.snb.2017.05.045
96	Multimode smartphone biosensing: the transmission, reflection, and intensity spectral (TRI)-analyzer	10.1039/c7lc00633k
97	A Novel Fiber Optic Surface Plasmon Resonance Biosensors with Special Boronic Acid Derivative to Detect Glycoprotein	10.3390/s17102259
98	Picomole Dopamine Detection Using Optical Chips	10.1007/s11468-016-0412-1
99	Specific Detection of Antibiotics by Silicon-on-Chip Photonic Crystal Biosensor Arrays	10.1109/JSEN.2017.2734885

Nº	TITLE_SCIENTIFIC PUBLICATION	DIGITAL OBJECT IDENTIFIER (DOI)
100	Acetone Biosensor Based on Fluorometry of Reduced Nicotinamide Adenine Dinucleotide Consumption in Reversible Reaction by Secondary Alcohol Dehydrogenase	10.1109/JSEN.2017.2721964
101	Immunodetection of salivary biomarkers by an optical microfluidic biosensor with polyethylenimine-modified polythiophene-C-70 organic photodetectors	10.1016/j.bios.2017.03.005
102	Label-Free Detection of DNA Hybridization Using a Reflective Microfiber Bragg Grating Biosensor With Self-Assembly Technique	10.1109/JLT.2017.2659778
103	Specific Detection of Aquaporin-2 Using Plasmonic Tilted Fiber Grating Sensors	10.1109/JLT.2016.2645233
104	Cancer biomarker sensing using packaged plasmonic optical fiber gratings: Towards in vivo diagnosis	10.1016/j.bios.2016.10.081
105	A SERS nano-tag-based fiber-optic strategy for in situ immunoassay in unprocessed whole blood	10.1016/j.bios.2016.10.070
106	Label-free detection of DNA hybridization with a compact LSPR-based fiber-optic sensor	10.1039/C7AN00249A
107	High-resolution and temperature-compensational HER2 antigen detection based on microwave photonic interrogation	10.1016/j.snb.2017.01.085
108	Development towards Compact Nitrocellulose-Based Interferometric Biochips for Dry Eye MMP9 Label-Free In-Situ Diagnosis	10.3390/s17051158
109	Stokes-Mueller matrix polarimetry system for glucose sensing	10.1016/j.optlaseng.2016.08.017
110	A novel U-bent plastic optical fibre local surface plasmon resonance sensor based on a graphene and silver nanoparticle hybrid structure	10.1088/1361-6463/aa628c
111	Highly sensitive optical biosensor based on silicon-microring-resonator-loaded Mach-Zehnder interferometer	10.7567/JJAP.56.04CH08
112	A Gelated Colloidal Crystal Attached Lens for Noninvasive Continuous Monitoring of Tear Glucose	10.3390/polym9040125
113	Interferometric detection of microRNAs using a capillary optofluidic sensor	10.1016/j.snb.2016.09.153
114	Label free ultrasensitive optical sensor decorated with polyaniline nanofibers: Characterization and immunosensing application	10.1016/j.snb.2016.08.103
115	Label-Free Biosensors Based on Bimodal Waveguide (BiMW) Interferometers	10.1007/978-1-4939-6848-0_11
116	Improved Sensitivity of Microring Resonator-Loaded Mach-Zehnder Interferometer Biosensor	10.18494/SAM.2017.1587
117	Wavelength-Scanning SPR Imaging Sensors Based on an Acousto-Optic Tunable Filter and a White Light Laser	10.3390/s17010090

№	TITLE_SCIENTIFIC PUBLICATION	DIGITAL OBJECT IDENTIFIER (DOI)
118	Detection of Aeromonas hydrophila Using Fiber Optic Microchannel Sensor	10.1155/2017/8365189
119	Fiber optofluidic biosensor for the label-free detection of DNA hybridization and methylation based on an in-line tunable mode coupler	10.1016/j.bios.2016.06.060
120	Temperature controlling fiber optic glucose sensor based on hydrogel-immobilized GOD complex	10.1016/j.snb.2016.06.062
121	An aptamer based method for small molecules detection through monitoring salt-induced AuNPs aggregation and surface plasmon resonance (SPR) detection	10.1016/j.snb.2016.06.035
122	Label-free bimodal waveguide immunosensor for rapid diagnosis of bacterial infections in cirrhotic patients	10.1016/j.bios.2016.04.095
123	Biosensor for label-free DNA quantification based on functionalized LPGs	10.1016/j.bios.2015.10.001
124	Localized Surface Plasmon Resonance-Based Micro-Capillary Biosensor	10.1109/LPT.2016.2582202
125	Label-free in-situ real-time DNA hybridization kinetics detection employing microfiber-assisted Mach-Zehnder interferometer	10.1016/j.bios.2016.02.065
126	Highly sensitive detection of urinary protein variations using tilted fiber grating sensors with plasmonic nanocoatings	10.1016/j.bios.2015.11.047
127	Introduction of an angle interrogated, MEMS-based, optical waveguide grating system for label-free biosensing	10.1016/j.snb.2015.11.072
128	Blu-ray optomagnetic measurement based competitive immunoassay for Salmonella detection	10.1016/j.bios.2015.08.070
129	Developing localized surface plasmon resonance biosensor chips and fiber optics via direct surface modification of PMMA optical waveguides	10.1016/j.colsurfa.2015.11.025
130	A Novel Optical Biosensing System Using Mach-Zehnder-Type Optical Waveguide for Influenza Virus Detection	10.1007/s12010-015-1902-x
131	A Localized Surface Plasmon Resonance-Based Portable Instrument for Quick On-Site Biomolecular Detection	10.1109/TIM.2015.2465691
132	Temperature-Compensating Fiber-Optic Surface Plasmon Resonance Biosensor	10.1109/LPT.2015.2492603
133	Novel Highly Sensitive Protein Sensors Based on Tapered Optical Fibres Modified with Au-Based Nanocoatings	10.1155/2016/8129387
134	A reflective mirror microcantilevers-based biosensor for biochemical detection	10.1016/j.ijleo.2015.11.103
135	Organic Semiconductor Laser Biosensor: Design and Performance Discussion	10.1109/JSTQE.2015.2448058
136	Interferometric-type optical biosensor based on exposed core microstructured optical fiber	10.1016/j.snb.2015.06.068

Nº	TITLE_SCIENTIFIC PUBLICATION	DIGITAL OBJECT IDENTIFIER (DOI)
137	A polydopamine-modified optical fiber SPR biosensor using electroless-plated gold films for immunoassays	10.1016/j.bios.2015.06.080
138	Monolithically integrated broad-band Mach-Zehnder interferometers for highly sensitive label-free detection of biomolecules through dual polarization optics	10.1038/srep17600
139	Label-free Single Molecule Detection Using Microtoroid Optical Resonators	10.1038/lsa.2016.1
140	A PMMA MICROFIBER COATED WITH AL-DOPED ZNO NANOSTRUCTURES FOR DETECTING URIC ACID	10.1016/j.measurement.2016.12.021
141	Surface Plasmon Resonance Biosensor Based on Smart Phone Platforms	10.1038/srep12864
142	PEG Functionalization of Whispering Gallery Mode Optical Microresonator Biosensors to Minimize Non-Specific Adsorption during Targeted, Label-Free Sensing	10.3390/s150818040
143	Label-free biosensing using cascaded double-microring resonators integrated with microfluidic channels	10.1016/j.optcom.2015.01.028
144	Tube Glass Waveguides Modified With Gold Nanoparticles for Application as a Simple Chemical and Biological Sensor	10.1109/JSEN.2014.2381234
145	A reusable aptamer-based evanescent wave all-fiber biosensor for highly sensitive detection of Ochratoxin A	10.1016/j.bios.2014.10.079
146	Multiplex Serum Cytokine Immunoassay Using Nanoplasmonic Biosensor Microarrays	10.1021/acsnano.5b00396
147	Linear readout of integrated interferometric biosensors using a periodic wavelength modulation	10.1002/lpor.201400216
148	Sensitive detection of 2,4,6-trinitrotoluene by tridimensional monitoring of molecularly imprinted polymer with optical fiber and five-branched gold nanostars	10.1016/j.snb.2014.10.079
149	Swallowable fluorometric capsule for wireless triage of gastrointestinal bleeding	10.1039/c5lc00770d
150	A novel single-layered MoS2 nanosheet based microfluidic biosensor for ultrasensitive detection of DNA	10.1039/C4NR07162J
151	A regenerative label-free fiber optic sensor using surface plasmon resonance for clinical diagnosis of fibrinogen	10.2147/IJN.S88963
