# Supplementary material for: A review of Optical Point-of-Care devices to Estimate the Technology Transfer of These Cutting-Edge Technologies
Source: Biosensors (Basel). 2022 Nov 29;12(12):1091. doi: 10.3390/bios12121091 (PMC9776401; doi:10.3390/bios12121091)
Supplement: Supplementary file 1 [file biosensors-12-01091-s001.zip › Table S4 Suppl_File.pdf]

| APPENDIX D_LINK SCIENTIFIC PUBLICATION_PATENT | | | | |
|---|---|---|---|---|
| Nº | TITLE_SCIENTIFIC PUBLICATION | DIGITAL OBJECT IDENTIFIER (DOI) | TITLE_PATENT | LINK GOOGLE PATENTS |
| 1 | Polymer-Coated Fiber Optic Sensor as a Process Analytical Tool for Biopharmaceutical Impurity Detection | 10.1109/TIM.2020.2981982 | US8476007B2<br>Optical fiber probe | https://patents.google.com/patent/US8476007B2/en?q=SENSOR&inventor=+Soumyo+Mukherji&type=PATENT. |
| 2 | 3-D Printed Instrumentation for Point-of-Use Leaky Waveguide Biochemical Sensor | 10.1109/tim.2020.2969036 | GB2587024A<br>A sensor | https://patents.google.com/patent/GB2587024A/en?oq=GB2587024A+A+sensor |
| 3 | Fiber optic nanogold-linked immunosorbent assay for rapid detection of procalcitonin at femtomolar concentration level | 10.1016/j.bios.2019.111871 | US20110090506A1<br>Self-referencing fiber-optic localized plasmon resonance sensing device and system thereof | US20110090506A1 - Self-referencing fiber-optic localized plasmon resonance sensing device and system thereof - Google Patents |
| 4 | Lossy mode resonance sensors based on nanocoated multimode-coreless-multimode fibre | 10.1016/j.snb.2019.126955 | ES2363285B2<br>Sensores de fibra optica recubierta basados en resonancia originada por modos con perdidas cercanos a la condicion de corte | Https://Patents.Google.Com/Patent/ES2363. |
| 5 | A New Device Based on Interferometric Optical Detection Method for Label-Free Screening of C-Reactive Protein | 10.1109/TIM.2018.2876073 | ES2334318B2<br>Sistema de deteccion optica para bio-ensayos de alta sensibilidad sinmarcado | ES2334318B2 - Sistema de deteccion optica para bio-ensayos de alta sensibilidad sinmarcado. - Google Patents |
| 6 | Hydrogel optical fibers for continuous glucose monitoring | 10.1016/j.bios.2019.05.002 | US11143815B1<br>Fiber optic integrated-light diffusers for sensing applications | US11143815B1 - Fiber optic integrated-light diffusers for sensing applications - Google Patents |
| 7 | Black phosphorus based fiber optic biosensor for ultrasensitive cancer diagnosis | 10.1016/j.bios.2019.04.044 | US10022322B2<br>Coating method | https://patents.google.com/patent/US10022322B2/en?oq=US10022322B2+Coating+method |
| 8 | Gold Nanoparticle-Functionalized Surface Plasmon Resonance Optical Fiber Biosensor: In Situ Detection of Thrombin With 1 n.M Detection Limit | 10.1109/JLT.2018.2822827 | US2021/0025945 A1<br>Fiber optic sensing apparatus, system, and method for state of charge measurement in energy storage devices | https://patents.google.com/patent/US20210025945A1/en?oq=US20210025945A1+Fiber+optic+sensing+apparatus%2c+system%2c+and+method+for+state+of+charge+measurement+in+energy+storage+devices |
| 9 | In-Situ Detection of Small Biomolecule Interactions Using a Plasmonic Tilted Fiber Grating Sensor | 10.1109/JLT.2018.2870337 | US20210025945A1<br>Fiber optic sensing apparatus, system, and method for state of charge measurement in energy storage devices | https://patents.google.com/patent/US20210025945A1/en?oq=US20210025945A1+Fiber+optic+sensing+apparatus%2c+system%2c+and+method+for+state+of+charge+measurement+in+energy+storage+devices |
| 10 | Microfluidics Integrated Lithography-Free Nanophotonic Biosensor for the Detection of Small Molecules | 10.1002/adom.201801313 | US10533941B2<br>Optical sensor platform employing hyperbolic metamaterials | https://patents.google.com/patent/US10533941B2/en?oq=US10533941B2+-+ |
| 11 | Disk-based one-dimensional photonic crystal slabs for label-free immunosensing | 10.1016/j.bios.2018.11.005 | WO2015118199A1<br>Sensor analítico y método de obtención del mismo | https://patents.google.com/patent/WO2015118199A1/es?oq=WO2015118199A1+Sensor+anal%C3%ADtico+y+m%C3%A9todo+de+obtenci%C3%B3n+del+mismo |
| 12 | Fully Integrated Liquid-Core Waveguide Fluorescence Lifetime Detection Microsystem for DNA Biosensing | 10.1109/ACCESS.2019.2934764 | US10405785B2<br>Determination of a concentration of an analyte in a subject | https://patents.google.com/patent/US10405785B2/en?oq=US10405785B2 |

| Nº | TITLE_SCIENTIFIC PUBLICATION | DIGITAL OBJECT IDENTIFIER (DOI) | TITLE_PATENT | LINK GOOGLE PATENTS |
|---|---|---|---|---|
| 13 | Real-time detection of prostate-specific antigens using a highly reliable fiber-optic localized surface plasmon resonance sensor combined with micro fluidic channel | 10.1016/j.snb.2018.07.007 | IE20170055A1 Surface plasmon resonance sensor device using microfluidic channel and system comprising the same | https://patents.google.com/patent/IE20170055A1/en?oq=IE20170055A1 |
| 14 | Optical conductivity-based ultrasensitive mid-infrared biosensing on a hybrid metasurface | 10.1038/s41377-018-0066-1 | US20200196925A1 Graphene-based nanosensor for identifying target analytes | https://patents.google.com/patent/US20200196925A1/en?oq=US+2020%2f0196925+A1+GRAPHENE+-+BASED+NANOSENSOR+FOR++IDENTIFYING+TARGET+ANALYTES |
| 15 | Highly sensitive colorimetric paper sensor for methyl isothiocyanate (MITC): Using its toxicological reaction | 10.1038/s41377-018-0066-1 | Colorimetric sensors for alkylating agents | https://patents.google.com/patent/WO2014193917A1/en?oq=WO2014193917A1 |
| 16 | Glucose Sensor Using U-Shaped Optical Fiber Probe with Gold Nanoparticles and Glucose Oxidase | 10.1038/s41377-018-0066-1 | US7196318B2 Fiber-optic sensing system | https://patents.google.com/patent/US7196318B2/en?oq=US7196318B2 |
| 17 | Phase-sensitive plasmonic biosensor using a portable and large field-of-view interferometric microarray imager | 10.1038/lsa.2017.152 | US20180136206A1 Reusable optical fiber aptasensor based on photo-thermal effect | US20180136206A1 - Reusable optical fiber aptasensor based on photo-thermal effect - Google Patents |
| 18 | Multimode smartphone biosensing: the transmission, reflection, and intensity spectral (TRI)-analyzer | 10.1039/c7lc00633k | US20180136206A1 Reusable optical fiber aptasensor based on photo-thermal effect | https://patents.google.com/patent/US20180136206A1/en?q=Reusable+optical+fiber+aptasensor+based+on+photo-thermal+effect&oq=Reusable+optical+fiber+aptasensor+based+on+photo-thermal+effect |
| 19 | Specific Detection of Antibiotics by Silicon-on-Chip Photonic Crystal Biosensor Arrays | 10.1109/JSEN.2017.2734885 | US9164026B2 Packaged chip for multiplexing photonic crystal microcavity coupled waveguide and photonic crystal slot waveguide devices for chip-integrated label-free detection and absorption spectroscopy with high throughput, sensitivity, specificity, and wide dynamic range | https://patents.google.com/patent/US9164026B2/en?oq=US9164026B2 |
| 20 | Acetone Biosensor Based on Fluorometry of Reduced Nicotinamide Adenine Dinucleotide Consumption in Reversible Reaction by Secondary Alcohol Dehydrogenase | 10.1109/JSEN.2017.2721964 | US20030164024A1 BIOSENSOR AND METHOD FOR PRODUCTION THEREOF | https://patents.google.com/patent/US20030164024A1/en?oq=US20030164024A1+BIOSENSOR+AND+METHOD+FOR+PRODUCTION+THEREOF |
| 21 | Immunodetection of salivary biomarkers by an optical microfluidic biosensor with polyethylenimine-modified polythiophene-C-70 organic photodetectors | 10.1016/j.bios.2017.03.005 | EP3443345B1 On-diaper body fluid screening device Dispositif de dépistage de fluide corporel dans une couche | https://patents.google.com/patent/EP3443345B1/de?oq=EP3443345B1+On-diaper+body+fluid+screening+device+Dispositif+de+d%C3%A9pistage+de+fluide+corporel+dans+une+couche |
| 22 | High-resolution and temperature-compensational HER2 antigen detection based on microwave photonic interrogation | 10.1016/j.snb.2017.01.085 | EP2228680A1 Photonic tunable filter and corresponding method of filtering electrical signals | https://patents.google.com/patent/EP2228680A1/de?oq=EP2228680A1+Photonic+tunable+filter+and+corresponding+method+of+filtering+electrical+signals |

| Nº | TITLE_SCIENTIFIC PUBLICATION | DIGITAL OBJECT IDENTIFIER (DOI) | TITLE_PATENT | LINK GOOGLE PATENTS |
|---|---|---|---|---|
| 23 | Development towards Compact Nitrocellulose-Based Interferometric Biochips for Dry Eye MMP9 Label-Free In-Situ Diagnosis | 10.3390/s17051158 | Sistema de detección óptica para ensayos biológicos de alta sensibilidad sin marcado | https://patents.google.com/patent/ES2574138T3/es?oq=ES2574138+(T3)++-++Optical+detection+system+for+labelling-free+high-sensitivity+bioassays https://patents.google.com/patent/US20030072519A1/en?oq=US2003072519+(A1)+Photonic+device%2c+o https://patents.google.com/patent/MX2018005253A/es?oq=MX2018005253+(A)+SENSOR%2c+APPARATUS+AND+METHOD+FOR+DETERMINING+A+CONCENTRATION+OF+A+SOLUTE+IN+A+SOLUTION. |
| 24 | Label free ultrasensitive optical sensor decorated with polyaniline nanofibers: Characterization and immunosensing application | 10.1016/j.snb.2016.08.103 | US8703505B2 Optical fiber probe | https://patents.google.com/patent/US8703505B2/en?oq=US8703505B2+Optical+fiber+probe |
| 25 | Wavelength-Scanning SPR Imaging Sensors Based on an Acousto-Optic Tunable Filter and a White Light Laser | 10.3390/s17010090 | US10578554B2<br>Spectrum-scanned SPR imaging detection system | https://patents.google.com/patent/US10578554B2/en?oq=US10578554B2+Spectrum-scanned+SPR+imaging+detection+system |
| 26 | Highly sensitive detection of urinary protein variations using tilted fiber grating sensors with plasmonic nanocoatings | 10.1016/j.bios.2015.11.047 | US9857290B2<br>Tilted grating sensor<br>FR2847989A1<br>Optical fibre with light guide, protective coating and a section structured as an optical filter of the Bragg grating type, has a coating of UV-crosslinked silicone elastomer over the filter section | https://patents.google.com/patent/US9857290B2/en?oq=US9857290B2 https://patents.google.com/patent/FR2847989A1/en?oq=FR2847989A1 |
| 27 | Introduction of an angle interrogated, MEMS-based, optical waveguide grating system for label-free biosensing | 10.1016/j.snb.2015.11.072 | US20160146729A1<br>Measurement method based on an optical waveguide sensor system<br>WO2014198409A1AB16<br>Integrated optical waveguide sensor system | US20160146729A1 - Measurement method based on an optical waveguide sensor system - Google Patents |
| 28 | Blu-ray optomagnetic measurement based competitive immunoassay for Salmonella detection | 10.1016/j.bios.2015.08.070 | EP2195461B1<br>Magnetic detection of small entities | https://patents.google.com/patent/EP2195461B1/de?oq=EP2195461B1+ |
| 29 | Interferometric-type optical biosensor based on exposed core microstructured optical fiber | 10.1016/j.snb.2015.06.068 | ES2579774T3<br>Detección de fluorescencia usando una fibra óptica microestructurada<br>EP 2176645 A1<br>Fluorescence sensing using a microstructured optical fiber | https://patents.google.com/patent/ES2579774T3/es?oq=ES2579774T3+Detecci%C3%B3n+de+fluorescencia+usando+una+fibra+%C3%B3ptica+<br><br>https://patents.google.com/patent/EP2176645A1/de?oq=EP+2176645+A1++Fluorescence+sensing+using+a+microstructured+optical+fiber |
| 30 | Monolithically integrated broad-band Mach-Zehnder interferometers for highly sensitive label-free detection of biomolecules through dual polarization optics | 10.1038/srep17600 | WO2009115847A1<br>Monolithically integrated physical chemical and biological sensor arrays based on broad-band mach-zhender interferometry | WO2009115847A1 - Monolithically integrated physical chemical and biological sensor arrays based on broad-band mach-zhender interferometry - Google Patents |

| Nº | TITLE_SCIENTIFIC PUBLICATION | DIGITAL OBJECT IDENTIFIER (DOI) | TITLE_PATENT | LINK GOOGLE PATENTS |
|---|---|---|---|---|
| 31 | Label-free Single Molecule Detection Using Microtoroid Optical Resonators | 10.1038/lsa.2016.1 | US20170322207A1 Label-free detection of nanoparticles and biological molecules using microtoroid optical resonatiors | https://patents.google.com/patent/US20170322207A1/en?oq=US20170322207A1 |
| 32 | Label-free biosensing using cascaded double-microring resonators integrated with microfluidic channels | 10.1016/j.optcom.2015.01.028 | US9052291B2<br>Optical sensor based on a broadband light source and cascaded waveguide filters | https://patents.google.com/patent/US9052291B2/en?oq=US9052291B2+Optical+sensor+based+on+a+broadband+light+source+an |
| 33 | Multiplex Serum Cytokine Immunoassay Using Nanoplasmonic Biosensor Microarrays | 10.1021/acsnano.5b00396 | CA2976678A1<br>Systems and methods for performing immunoassays | CA2976678A1 - Systems and methods for performing immunoassays - Google Patents |
| 34 | Swallowable fluorometric capsule for wireless triage of gastrointestinal bleeding | 10.1039/c5lc00770d | US20210204833A1<br>System and method for wireless biosensor monitoring | https://patents.google.com/patent/US20210204833A1/en?oq=US20210204833A1+System+and+method+for+wireless+biosensor+monitoring |
